# Early Stimulation and Nutrition: The Impacts of a Scalable Intervention

**DOI:** 10.1093/jeea/jvac005

**Published:** 2022-01-28

**Authors:** Orazio Attanasio, Helen Baker-Henningham, Raquel Bernal, Costas Meghir, Diana Pineda, Marta Rubio-Codina

**Affiliations:** Yale, USA; IFS, UK; and FAIR at NHH, Norway; Bangor University, UK; and University of the West Indies, Jamaica; CEDE, Universidad de los Andes, Colombia; Yale, USA; JPal, USA; and IFS, UK; Fundación Éxito, Colombia; Inter-American Development Bank, USA

## Abstract

Early childhood development is becoming the focus of policy worldwide. However, the evidence on the effectiveness of scalable models is scant, particularly when it comes to infants in developing countries. In this paper, we describe and evaluate with a cluster-Randomized Controlled Trial an intervention designed to improve the quality of child stimulation within the context of an existing parenting program in Colombia, known as FAMI. The intervention improved children’s development by 0.16 of a standard deviation (SD) and children’s nutritional status, as reflected in a reduction of 5.8 percentage points of children whose height-for-age is below -1 SD.

## 1. Introduction

Human capital, important as it is for life outcomes (Becker 1994) and economic development, is undermined by poverty from the very beginning of life. This, in turn, leads to a vicious cycle: The underachievement of individuals from deprived backgrounds contributes to the intergenerational persistence of poverty. It is now widely understood that the early years of brain development, and indeed the first 1,000 days, can be particularly important for adult outcomes, with the experiences during early childhood having a long-lasting impact.^1^

Over the last couple of decades, our understanding of the process of child development and the evidence on the types of interventions that might improve outcomes have advanced significantly (Black et al. 2017; Britto et al. 2017). In particular, the potential of parenting support programs to improve child development, especially in vulnerable contexts, has been amply demonstrated (Neville, Pakulak, and Stevens 2015; Britto et al. 2017).

Given the established knowledge, early years interventions should aim at improving the ability of parents to provide responsive and emotionally supportive environments and ensure developmentally stimulating opportunities for their children (Bradley and Putnick 2012; Singla, Kumbakumba, and Aboud 2015; Black et al. 2017), while at the same time be implementable at realistic cost levels and given the available implementation infrastructure, including personnel. If well-designed and adequately targeted to the appropriate age and population subgroups, then these programs may be crucial in breaking the intergenerational transmission of poverty.

Indeed, governments around the world have recognized the importance of the early years and have started to introduce services to support children from deprived backgrounds. Head Start in the United States and Sure Start in the United Kingdom are prime examples in developed economies, while the Cuna Má s in Peru and the Family, Women, and Childhood program (FAMI for its acronym in Spanish) in Colombia, which is our focus in this work, are similar examples in low- or middle-income countries (LMICs). Indeed, an increasing number of countries now have national early childhood policies Devercelli, Sayre, and Denboba (2016).

Although at-scale early years programs are becoming widespread, evidence on their long-term effectiveness, that is, their ability to improve early childhood development (ECD) outcomes in a manner that translates into improved functioning and well-being later in life, is limited. Long-run impacts will likely vary depending on the details of *what* they actually offer and *how* they actually offer it. Understanding their effectiveness in the context of LMICs is even more important than in high-income countries, as poverty levels are higher, risk factors such as malnutrition are more prevalent, and resources are more limited.

In this paper, we go beyond the standard approach of evaluating an existing program, such as the work on Head Start (Bitler, Hoynes, and Domina 2014; Kline and Walters 2016) and Early Head Start (Love et al. 2005). Instead, we design and evaluate with a clustered Randomized Controlled Trial (c-RCT) a *scalable* intervention aimed at improving an existing parenting program run by the government. The intervention we studied involved the introduction to FAMI, an *existing government program*, of (i) a *structured early stimulation curriculum*, delivered through weekly group sessions with mothers and children, and monthly individual home visits; (ii) *training and coaching of the personnel delivering the intervention*, provided by trained mentors (tutors, henceforth); and (iii) an enhanced *nutritional supplement* for beneficiary children, alongside with nutrition education.^2^ By collaborating with the government and using the existing infrastructure (i.e. program structure and personnel), we place the intervention within an operating institutional setting, which facilitates reaching scale.

The main question we are asking is whether offering early stimulation and appropriate nutrition in poor environments in a manner designed to be scalable by building on a nationwide program implemented by a government agency can still improve children’s human capital and ultimately mitigate the effects of poverty. In our context, the scalability of an intervention depends not only on its cost, but also on the possibility of running the intervention within an institutional framework that can handle it effectively. This is a key policy question, as well as one that adds to the evidence on the importance of early childhood interventions.

FAMI brings together mothers and their infants in a group setting with other mother-child dyads. Sessions are run by a local woman employed by the government, the FAMI mother. We developed a program adapted to these circumstances and inspired by the original Jamaica home visiting intervention (Grantham-McGregor and Smith 2016), now known as *Reach Up* (RU, see Grantham-McGregor and Walker 2015; Walker et al. 2018), and its replication in a scalable fashion in Colombia (Attanasio et al. 2014, 2020; Andrew et al. 2018).

The intervention was randomly allocated to 46 of 87 municipalities located in three of Colombia’s 32 departments and lasted an average of 10.4 months. Mothers in the control communities still had the option of attending the existing program (FAMI). In other words, the counterfactual against which treatment is compared is FAMI running as usual, and not the complete absence of the program (see Kline and Walters 2016).

On an intention to treat basis, our intervention significantly improved children’s cognitive development by 0.16 (*p*-value 0.044) of a standard deviation (SD), with an implied average treatment on the treated (ToT) effect of 0.3 SD–0.4 SD, depending on how we define compliance and intensity of treatment. As our end-line data were collected so that children were exposed to the treatment for at most 10 months, we also perform a dosage analysis, where variations in exposure were due to differences in the timing of the training of facilitators. Our analysis shows that the impact increases with increased intervention exposure. We also find some evidence of heterogenous impacts, with larger impacts for beneficiaries in the poorest households. This is consistent with the findings from another at-scale early years intervention (Bitler, Hoynes, and Domina 2014), although this study focuses on children older than those we consider.

Children’s nutritional status also improved: The fraction of children whose height-for-age is below -1 SD declined by 0.058 (*p*-value 0.10) with a corresponding increase in those with height-for-age between -1 SD and 1 SD (0.074 increase in height-for-age, *p*-value = 0.036).^3^ Results on the long-term effects of nutritional interventions are scarce and generally mixed. While short-term positive impacts were sustained in Guatemala (Hoddinott et al. 2013), in the Jamaica experiment, powdered milk supplementation showed important impacts early on which faded out in the longer term (Walker et al. 2005, 2006, 2011). In both studies children were stunted at baseline. In Attanasio et al. (2014), micronutrient supplementation to a population of children with no specific nutritional deficit had no effect.

In addition to the main impacts, we also explore the mechanisms through which these might have been achieved. After providing evidence that the intervention significantly increased some potential mediators, such as parental investment in children, we show that indeed parental investment can explain most of the observed impact on child development using simple mediation analysis. This finding is confirmed by the results from a structural model that accounts for the endogeneity of parental investment in the estimation of a result consistent with that in Attanasio et al. (2020).

In addition to the impacts of the intervention and its implementability, which we have discussed so far, we need to consider its cost, which is about $322 per year per child, plus $11 per child for annual pre-service training. The cost of the unenhanced FAMI program is itself about $327 per child per year; our intervention, therefore, roughly doubles the cost of the existing program. This might seem high; however, a comparison of the costs and impacts of several early years interventions promoted by the Colombia government in the same period (see Table B.1 in Online Appendix B), suggests that, given cost, interventions improving process quality by introducing a curriculum and improving child rearing practices,^4^ have higher impact than those improving infrastructure quality alone (such as buildings and staffing). In our case, the impact of the intervention, deployed for 10 months, is 0.162 SD compared to a total cost of USD 322. Moreover, as we have suggestive evidence that the impacts increase with additional sessions, the reported impact is likely to be an underestimate of the total impact. Therefore, the evidence presented in this paper suggests that it is possible to gradually improve the quality of nationwide programs at scale in a way that is both impactful and affordable.

Our findings demonstrate the potential for improving human capital in poor settings and therefore form the basis for policy in a broader set of contexts across LMICs and contribute to the limited existing literature on the scalability of ECD interventions. The evidence on the long-term impacts of parenting interventions is mainly from small efficacy trials.^5^ However, the evidence on the short- and medium-term impacts of scalable or at-scale parent support programs, that is, interventions designed to improve outcomes for a large number of children, is scarce and inconclusive both in high-income countries and LMICs.

Relevant studies in high-income countries include Robling et al. (2016) for the evaluation of NFP in the United Kingdom, Cattan et al. (2019) for that of Sure Start in the United Kindom, Love et al. (2005) for Early Head Start in the United States, and Hjort et al. (2017) in Denmark. In LMICs, most of the few existing studies report on short-term impacts’such as the evaluation of the nationwide Cuna Mas Program in Peru (Araujo et al. 2021), the evaluation of a group-based intervention delivered within a nationwide conditional cash transfer (CCT) program in Mexico (Fernald et al. 2017b), program integrations within primary health clinics in the Caribbean (Chang et al. 2015) and Bangladesh (Hamadani et al. 2019), or an evaluation comparing home visits versus group delivery in India (Grantham-McGregor et al. 2020). Two exceptions, which investigate impacts approximately two years after the end of intervention activities, are the studies in Colombia, where early stimulation and supplementation were delivered within the infrastructure of the country’s CCT (Attanasio et al. 2014; Andrew et al. 2018), and in Pakistan, where these were integrated into an existing community-based health service (Yousafzai et al. 2014, 2016). Scalability of effective and sustainable interventions is therefore a major and salient challenge.

The evidence we present also has direct implications for the importance of safety-net programs, such as Food Stamps in the United States (see Hoynes, Schanzenbach, and Almond 2016), for child outcomes. These programs can improve nutrition for children by providing more resources to parents. We show that providing such nutritional supplementation directly (in combination with child stimulation) can be an effective way of improving children’s nutritional status, implying that parents do not appear to crowd out the additional resources provided for the children, even when they are delivered for use at home, as in our case. The absence of (complete) crowding out is a key element for understanding whether such programs can work and the extent to which they do.

The rest of the paper is organized as follows. The next section describes the context, the existing program, and the add-on intervention we evaluate. In Section 3, we discuss the evaluation design and sample. Section 4 presents the empirical strategy, and Section 5 the main evaluation results. Section 6 investigates the mechanisms behind the impacts obtained, and, finally, Section 7 discusses policy implications and concludes.

## 2. Background and Intervention

The intervention that we evaluate consists of improving FAMI, an existing program run by the Colombian Family Welfare Agency (ICBF for its acronym in Spanish), a government institution. The fact that the innovation we are considering is grafted on a pre-existing infrastructure is important both for interpreting the size of its impacts and to provide a genuinely scalable model. In this section, we first describe the existing program and then describe the improvements that we test.

### 2.1. Description of the Existing Parent Support Program, FAMI

The FAMI program is aimed at supporting vulnerable families during pregnancy, childbirth, early childhood with nutrition, health monitoring, and childrearing. Beneficiaries are identified by their score in SISBEN, Colombia’s proxy means test based on household socio-economic characteristics and used for targeting most social policies. For the child stimulation component, the program is delivered through weekly group sessions of one hour each and a monthly home visit of about an hour for parents of children 0–24 months of age. Group meetings take place in community spaces, such as schools and churches, or the FAMI facilitator’s own home. Based on ICBF’s nationwide administrative data from 2013, prior to the beginning of this study, the size of each FAMI unit varies between 10 and 24 beneficiaries with a mean of 13 (SD = 1.4). Approximately 80% of the beneficiaries are parents of children 0–24 months of age, and 20% are pregnant women. Close to 225,000 families were FAMI beneficiaries around 2013, when this study started. FAMI mothers, the program facilitators, are local women and generally have a high school degree but no specific training on ECD. Similarly, the program has no concrete curriculum, other than some general operational guidelines and broad learning standards.^6^ Indeed, during the pilot stage, we observed a rather diverse set of activities and discussions during the group sessions, with little-to-no engagement of the children. The monthly home visits were not designed around stimulation activities for the child but involved general advice for the family. The program also delivers a nutritional supplement that corresponds to 22%–27% of the (monthly) recommended calorie intake of children younger than two and pregnant women. The average cost of the pre-existing FAMI program is $318 US (US dollars or USD) per child per year (Bernal 2013). Further details on the pre-existing program and on the nature of the changes we introduced are provided in Online Appendix A.

### 2.2. Description of the Intervention

The intervention we evaluate aims to enhance the existing program through three complementary elements: (i) a structured early stimulation curriculum to improve child development, accompanied by pedagogical materials such as books, puzzles, and toys; (ii) training and coaching for the FAMI mothers; and (iii) a larger and higher quality nutritional supplement than that previously received by FAMI participants, along with nutrition education during group sessions and home visits, and other materials such as recipe books and cards with age appropriate nutrition messages.

The stimulation curriculum was based on RU (Grantham-McGregor and Walker 2015; Walker et al. 2018), adapted, for the most part, to group meetings. FAMI includes, however, a monthly home visit whose content was, again, adapted from RU. Both group meetings and home visits, last for about an hour, and aim at improving parenting practices and at introducing developmentally appropriate activities for children, in particular, activities that promote language, cognitive, and fine motor development.

Mothers are encouraged to practice stimulation activities on a daily basis. Although most of the program content was delivered through the weekly group sessions, the monthly home visits were used to better tailor the activities to the developmental level of each child and to introduce other, possibly more complex, activities. With respect to RU, the adapted curriculum added group discussions, more language activities, activities for children aged from birth to 6 months, and cards with nutrition information. The program also trained mothers in sensitive and responsive parenting and appropriate behavior management, promoting positive interactions, discouraging child mistreatment, and ultimately promoting child socio-emotional development. The curriculum was designed to be delivered by facilitators without specialized knowledge of child development. For this reason, it was purposefully quite prescriptive.

Separate group meetings were offered for pregnant and lactating women with children up to 6 months, mothers with children 6–11 months, and mothers with children aged 1–2 years. However, as in practice, mothers did not keep to their allocated slots, we ensured that the session would cater to children of different ages, with age-appropriate activities for all. An average of five mothers attended each session (min = 1, max = 15, SD = 2.6). The curriculum involved materials to be used during the sessions, including age-appropriate books, puzzles, home-made toys, pictures, construction blocks, and nutrition cards. The intervention also included supplementary sessions to teach mothers how to construct home-made toys with recyclable materials that could be used to practice the activities proposed at home. This way, most mothers were able to set up a toy library for home use. All materials used in the session were taken home for practice and returned the following week.^7^

Pregnant women were invited to participate in all sessions and were encouraged to practice the activities along with the other mothers and their babies. However, in this study, we focus on the impacts of the intervention on children 0–24 months only.

A team of nine tutors, with college degrees in psychology and social work, trained and supervised by the research team, trained the FAMI mothers in the intervention before it started. Training was provided sequentially by the town. All FAMI mothers in each given town were trained simultaneously for an average of 3.5 weeks and 85 hours.^8^ The training involved demonstration, practice, and feedback in running the group sessions and in conducting the play and language activities with mothers and children, and in learning how to make the home-made toys. After the initial training was finalized, the tutors coached the FAMI mothers continuously throughout the duration of the intervention. In each supervision round, which took place approximately every 6 weeks, tutors observed one group session and one home visit, after which they provided feedback to the FAMI mother. Each tutor oversaw 5 towns and 19 FAMI mothers, on average. The tutors were, in turn, supervised by a program supervisor (a member of the research team) who visited each tutor every 2 months.

In short, the curriculum we introduced was intended to add both structure and content to the on-going sessions. FAMI mothers in the treatment group found the intervention to be substantially different to what was going on in the status quo, with 82% reporting they found it differed from their usual practice.^9^

Lastly, the intervention also included a monthly nutritional supplement that provided 35% of the daily calorie intake requirements for target children.^10^ The nutritional content of the supplement was specifically targeted either for the pregnant mothers or to each child depending on their age, see Online Appendix A for further details.^11^ All supplements were delivered monthly to the FAMI facilitator, who was in charge of distributing them among program participants during the first group session of each month. Families would not receive the monthly nutritional supplement if they did not attend this session. So, in a way, the early stimulation component represented a conditionality for receiving the supplement.

Clearly, crowding out of other nutrition and sharing within the household is a central concern. Participants were told that the beneficiary of the supplement was the child. However, there was no way to guarantee that its content was appropriately used in the home or the extent to which it was (exclusively) offered to the target child.^12^ We can only provide suggestive evidence, based on the program outcomes.

Table 1 presents the running cost of the existing program, in the first column, alongside the additional cost of the intervention, the improvement we evaluate, in the second column. Costs are presented by component, showing a total program cost with and without nutrition. All values in Table 1 are expressed in USD per year per child, using the exchange rate at the time of the intervention and assuming an average FAMI size of ten mother-child pairs.^13^

**Table 1.** Costs of the original program and its improvement US ${\$}$ per child per year.

	Original program	Additional intervention costs
Materials	${\$}$ 8	${\$}$ 27
Other administration costs	${\$}$ 2	–
Salary FAMI mother	${\$}$ 240	–
Mentoring	0	${\$}$ 88
Total without nutrition	${\$}$ 250	${\$}$ 115
Nutrition	${\$}$ 77	${\$}$ 209
Total with nutrition	${\$}$ 327	${\$}$ 322
FAMI training	N.A.	${\$}$ 11 one-time cost

The cost of the intervention we are evaluating, which is relevant both for its scalability and cost-effectiveness, should not be the same as the cost of the original program. As shown in Table 1, a substantial part of the cost of the original program is the salary of the FAMI mothers, which did not change, as the intervention did not hire additional FAMI mothers or decrease the number of children served by each FAMI. However, a substantial component of the cost of improving the existing program is the monitoring and mentoring that the FAMI mothers now receive. This amounts to ${\$}$88 US per year per child, which covers the salaries of the tutors. For comparison, the FAMI mother’s salary corresponds to ${\$}$240 US per child per year. Including the ${\$}$27 US for materials yields a total cost of the coaching component of ${\$}$115 US. Excluding the nutritional component in both the original program and this intervention, the FAMI intervention we are considering increases the cost of the program by about 46%. We consider the initial facilitator training (${\$}$11 US) as a one-off expense to be incurred in the first year. As it could benefit subsequent cohorts of children, it should be seen as an investment with some durability.^14^ The largest increase in cost comes from the added nutritional package, which costs 2.71 times more than what it regularly costs, from ${\$}$77 US to ${\$}$209 US per child per year. Overall, the total increase in the cost of the program is of ${\$}$322 US (or ${\$}$333 US adding the one-off initial training), which effectively amounts to doubling the original cost of ${\$}$327 US per child per year. Online Appendix A offers additional details on the cost of each component, and Online Appendix B includes a more thorough discussion on costs and scalability.

## 3. Sampling Design, Descriptive Statistics, and Implementation

The study took place between September 2014 and July 2016. At the start of the project, we prepared a pre-analysis plan and registered the trial at the ISRCTN registry (Online Appendix H).^15^ The intervention was intended to operate for 15 months between the end of 2014 and March 2016. In practice, the total duration varied by community, mainly to accommodate the initial training, and lasted an average of 45 weeks (10.4 months) with a range of 34–58 weeks. The logistics of rolling out the intervention implied a considerable amount of variation in exposure for the target children, mainly due to organizational issues.

The study towns were located in three departments in central Colombia (Cundinamarca, Boyacá, and Santander). They were all chosen to have (i) fewer than 40,000 inhabitants, to avoid large urban centers; (ii) at least two FAMI units;^16^ and (iii) no more than one unit of another public parenting program called *Modalidad Familiar* (MF) to minimize attrition towards this alternative program. MF is a public parenting program, similar to FAMI, that was introduced during the first half of 2014.^17^ The presence of MF is balanced between control and treatment sample towns, so we are de facto estimating the effect of enhancing the FAMI program in the presence of some MF. Importantly for interpreting the results of our evaluation, the presence of MF in the study sample is minimal, with only 7% of the target children leaving FAMI to join MF. We further discuss this issue below.

Out of a universe of 135 such towns in these departments, we randomly drew 49 for the treatment group and 47 for the control. We assigned the remaining 39 towns to a randomly ordered waiting list. Towns in this waiting list were used to replace towns that had completely transitioned to the new MF program (whether in treatment or control). We could successfully replace 10 of the 19 towns that no longer ran the FAMI program, which yielded a final sample of 87 towns: 46 in the treatment group and 41 in the control group.

The average number of children younger than two per FAMI unit in the sample was 9.5 (SD = 2.9), and the average number of pregnant women was 2.1 (SD = 1.7). This implies an average of 11.6 (SD = 2.8) total beneficiaries per FAMI unit. Within each unit, we enrolled in the study all children under 12 months of age at baseline, leading to a sample of *N* = 1,460 children (4.3 children per FAMI and 17 per town, on average). We chose this subsample of children in order to maximize the potential time of exposure to our intervention, before children outgrow the FAMI program at age two. Overall, a total of 702 children in 171 FAMI units in 46 towns received the treatment (our enhanced version of the FAMI program); and 758 children in 169 FAMI units in 41 towns were in the control group, and therefore continued to receive the FAMI program as usual. At follow-up, we tried to reach all children in the study sample, regardless of whether they were still attending a FAMI or not, and regardless of the length of their exposure to FAMI.

Online Appendix C provides further details on the study design, including power calculations, the study flow of participants, and the geographic distribution of treatment and control towns.

### 3.1. Data

As described in the pre-analysis plan, reported in Appendix H, we defined a number of primary outcomes. These included measures of nutritional status, namely, externally standardized height-for-age *Z*-scores, constructed following the World Health Organization (WHO) standards (Bayley 2006); and socio-emotional development, as measured by the Ages and Stages Questionnaire: Socio-Emotional (ASQ:SE) (Squires, Bricker, and Twombly 2009). We chose developmental tests that have been extensively used in evaluations of early care or education and/or have been recommended for LMICs (Fernald et al. 2017a). These instruments were either available in Spanish or had been previously translated, as they had been used in Colombia before among similar populations. Anthropometric measures were collected in both rounds, whereas developmental measures were only collected at follow-up. At baseline, children were younger than one year of age. Given the limited resources we had and how complex and expensive it is to reliably assess the development of such young children, we decided not to.^18^

For the analyses, we used internally age-standardized Bayley-III scores, where raw scores were standardized using the sample mean and SD calculated from weighted local smoothing regressions. We also aggregated all Bayley-III subscales using the factor model described in Online Appendix D, which we interpret to reflect the child’s “cognitive” development. Children with extreme values for developmental or nutritional outcomes, according to international standards, were excluded from the analyses.^19^

In order to obtain an understanding of the mechanisms at play, we also estimate impacts on intermediate outcomes that could have mediated the effect of the intervention on children’s developmental outcomes. In particular, we collected by maternal report, both at baseline and at follow-up, information on variables that measure the quality of the home environment, maternal self-efficacy, maternal knowledge about child development, and food insecurity.

For the quality of the home environment, we used four variables constructed from items in UNICEF’s Family Care Indicators (FCI, Kariger et al. 2012): the number of magazines, books, or newspapers in the home; the number of toy sources; the number of varieties of play materials in the home; and the number of varieties of play activities the child engaged in with an adult over the 3 days before the interview, which were summarized in a single factor, labelled “parental investment” and estimated using the factor model described in Online Appendix D. We assessed maternal self-efficacy using the self-efficacy in the nurturing role scale in Porter and Hsu (2003). This scale contains 16 items rated in 7-point scales that pertain to mothers’ perceptions of their competence on basic skills required in caring for an infant. To measure maternal knowledge about child development, we used 10 items, some selected from the Knowledge of Infant Development Inventory (KIDI, MacPhee 1981) and some developed by the research team.

Food insecurity was collected with the Latin American Scale for the Measurement of Food Insecurity (ELCSA scale), both at baseline and at follow-up. The ELCSA had been previously validated in Colombia (Álvarez Uribe and Instituto Colombiano de Bienestar Familiar 2008) and allows classifying households in four food insecurity levels: secure, mild insecurity, moderate insecurity, and severe insecurity (Álvarez Uribe and Instituto Colombiano de Bienestar Familiar 2008). In the analysis, we use an indicator that equals 1 if the household is food insecure (mild, moderate, or severe) and 0 otherwise.

Detailed socio-economic household information was also collected, including maternal vocabulary scores (a proxy for maternal IQ), which was assessed on the Spanish version of the Peabody Picture Vocabulary Test (PPVT), Test de Vocabulario en Imagenes Peabody (TVIP) (Padilla, Lugo, and Dunn, 1986).

Finally, background information on FAMI mothers was gathered directly from them in both rounds. In addition to basic socio-demographic characteristics, we also collected their vocabulary scores and knowledge of child development using the same tests as for mothers.

### 3.2. Descriptive Statistics

Table 2 shows baseline characteristics by treatment status. At baseline, children were, on average, for both the treatment and control groups, 5.6 months of age, and in about 27% of the cases, the father was absent from their household. Households had two children, on average; maternal average schooling was 8.6 years; and 23% of mothers were teenagers. In 2010, the teenage pregnancy rate was 21% nationwide and 30% for young girls living in households in the poorest income quintile.

**Table 2.** Sociodemographic characteristics of children and their families at baseline.

	Treatment	Control	*p*-value	RW
Sociodemographic characteristics				
Child’s age in months	5.72	5.51	0.353	0.945
	(3.39)	(3.26)		
Child’s birth weight (gr)	3189	3156	0.442	0.956
	(572)	(500)		
Maternal age (number of years)	26.16	26.47	0.421	0.956
	(6.84)	(6.70)		
Maternal years of schooling	8.85	8.41	0.121	0.688
	(3.42)	(3.31)		
Household Income (COP thousands)	526.1	477.2	0.232	0.883
	(388.1)	(340.7)		
Household size	4.08	4.10	0.932	0.976
	(1.47)	(1.43)		
Maternal PPVT (raw score)	22.32	19.76	0.037	0.386
	(8.53)	(8.08)		
Child’s gender (% male)	51.9	50.9	0.729	0.976
First born (%)	46.6	45.1	0.655	0.976
Teenage mothers (%)	25.4	20.9	0.059	0.508
Father present (%)	69.7	75.1	0.031	0.386
Owns home (%)	37.1	39.6	0.623	0.976
Household in poverty (%)$^{\rm a}$	58.7	64	0.298	0.920
Intermediate outcomes				
Parental Investment$^{\rm b}$	-0.03	0.03	0.625	0.948
	(0.96)	(1.02)		
Maternal knowledge$^{\rm c}$	29.26	29.49	0.680	0.948
	(3.61)	(3.44)		
Maternal self-efficacy	26.50	26.49	0.974	0.978
	(5.51)	(4.67)		
Food insecurity (%)	50.4	41.9	0.219	0.631
No. of observations	700	756		

Notes. Standard deviations (clustered by town) in parentheses. RW: *p*-values adjusted for multiple testing using the Romano–Wolf (Romano and Wolf 2005, 2016) step-down method. In this case all hypotheses in the panel are included in the RW *p*-value calculation. Household Income is measured in thousands of Colombian Pesos (COP).

a. % of households with total income below the poverty line in 2014 (${\$}$50 US person/month).

b. Factor score of FCI subscales.

c. Only available at follow-up (raw scores presented).

The target population was particularly poor: Average household income was COP 501,000 per month (US 178), which represents 81% of the legal monthly minimum wage in 2014. Close to 70% of these households had answered the SISBEN survey for screening of social program eligibility, a good proxy for poverty, and 96% of those surveyed were deemed eligible for social programs (i.e. they scored in SISBEN levels 1 and 2). Similarly, 62% of households in the sample had a total income below the poverty line adjusted for household size. In 2014, the poverty rate was 42% in semi-urban and rural areas of Colombia.

The environment in which the sample children grew up is highly deprived: In terms of the home learning environment (“parental investment”), on average, these households owned 2.6 books, magazines, or newspapers and 1.4 different varieties of play materials for young children in the household, and adults were reported to have engaged in 2.5 different types of play activities with young children over the past 3 days.^20^ For comparison, among a representative sample of low-middle-income households with children aged 6–12 months in Bogota (Colombia’s capital city), we observed an average of 3.2 different varieties of play materials and 3.4 different types of play activities. Moreover, the median household in this sample only owned three books for adults.

In Table 3, we show averages for the baseline nutritional status of children by treatment status. Specifically, we report weight-for-age, height-for-age, and height-for-weight *Z*-scores, in addition to a variety of nutritional indicators by deficit or excess as identified by international standards. In our sample 12% of the children are stunted. For comparison, stunting was about 9.3% for children younger than one year of age in rural areas in Colombia in 2013 and 11.8% in urban areas (as measured in the Colombian Longitudinal Household Survey, CEDE 2013). Table 3 also shows that an additional 15% of children were at risk of stunting, that is, children whose height-for-age was between -2 SD and -1 SD.

**Table 3.** Nutritional status of children at baseline by randomization status.

	Treatment	Control	*p*-value	RW
Weight-for-age *z*-score	0.26	0.27	0.921	0.988
	(1.39)	(1.42)		
Length/height-for-age *z*-score	-0.01	-0.21	0.241	0.797
	(1.68)	(1.74)		
Weight-for-length *z*-score	0.37	0.55	0.167	0.749
	(1.59)	(1.65)		
Underweight (%)	6.4	5.1	0.465	0.918
Risk of underweight (%)	9.1	10.7	0.415	0.918
Wasting (%)	5.9	6.4	0.775	0.988
Risk of wasting (%)	10.9	8.2	0.179	0.749
Stunting (%)	9.2	13.9	0.081	0.501
Risk of stunting (%)	14.7	15.5	0.793	0.988
Overweight (%)	9.9	9.2	0.707	0.988
Obesity (%)	4.8	7.3	0.174	0.749

Notes. Standard deviations (clustered by town) in parenthesis. Adjusted *p*-values using the Romano–Wolf (Romano and Wolf 2005, 2016) procedure (2,000 iterations, clustered by town) are included in the last column. All variables in the table are considered as one group of hypotheses. Underweight: weight-for-age $<$ -2 SD; risk of underweight: weight-for-age between -1 SD and -2 SD; wasting: weight-for-height $<$ -2 SD; risk of wasting: weight-for-height between -1 SD and -2 SD; stunting: height-for-age $<$ -2 SD; risk of stunting: height-for-age between -1 SD and -2 SD; overweight: weight-for-height between 2 SD and 3 SD; and obesity: weight-for-height $>$ 3 SD.

In Table 4, we report the mean and standard deviation of the cognitive, language, and socio-emotional development levels for the control group as measured at follow-up (ages 17–33 months). These have been standardized with a mean of 100 and a standard deviation of 15, which is the US reference population (composite scores). Subject to all the caveats of such comparisons, this allows us to place our population relative to the expected developmental outcome under favorable conditions. The Bayley-III composite scores were 0.6 SD below the norming sample mean in both the cognitive and language scales, and 0.4 SD below in the motor scale. We also observed that 18% of children score between -1 SD and -2 SD with respect to the norming sample in cognition, 23% in language, and 15% in motor development. Only about 2%–3% would be considered at risk of developmental delay given that their composite scores are below -2 SD.

**Table 4.** Developmental outcomes of children in the control group at follow-up.

	Mean (standard deviation)	*N*
**Bayley**		
Cognitive composite score	91.98 (13.07)	703
Language composite score	91.59 (12.31)	702
Motor composite score	93.97 (12.58)	701
**ASQ:SE**		
% of children at socio-emotional risk	0.38	705

Notes. Standard errors are clustered by town in parenthesis. Bayley-III composites are computed based on external standardization provided by test developers. The fraction of children at socio-emotional risk by the ASQ:SE is computed using the thresholds provided by the test developers (Squires, Bricker, and Twombly 2009).

In terms of socio-emotional development, 38% of the children were at risk of developmental delay according to thresholds defined by the ASQ:SE using the test norming sample. For comparison, we know from the CEDE 2013 that 22% of children younger than two in low-Socioeconomic Status (SES) urban households were at risk of developmental delay by the same measure, 26% in high-SES urban households, and 19% in rural households in 2013.

Finally, in Online Appendix E, we present the basic characteristics of FAMI mothers by study group. On average, they were 42 years of age, had completed 13 years of education, and had almost 12 years of work experience in the FAMI program. They had an average of 2.5 children of their own. There were no jointly significant differences between FAMI mothers in treatment and control towns.

### 3.3. Attrition, Compliance, and Dosage

In both treatment and control towns, children in the sample might be “lost” in the follow-up survey and/or might drop out of FAMI. The first is an attrition problem, while the latter is a compliance one. At follow-up, we attempted to reassess all children, including those who dropped out of FAMI, to avoid non-random selection.

We report figures on attrition in Online Appendix F (Table F.1). The attrition rate, at 8.6%, was slightly higher in the treatment group (10.6%) than in the control group (6.7%), although the difference is significant only at the 10% level. Children lost at follow-up were older, less likely to have a resident father at home, and more likely to have mothers with lower vocabulary (PPVT) scores. Moreover, as shown by the interactions of the treatment indicator with observables, attrition affected slightly the composition of the treatment and control samples (third column of Online Appendix Table F.1). While the attrition differential between treatment and control towns was not very large, in Online Appendix G, we discuss how we deal with the potential bias that it could introduce to our impact estimates. Furthermore, there we show that attrition does not bias our main findings.

Children who dropped out of the FAMI program between baseline and follow-up, if found, were interviewed at follow-up and their families were asked for the reason to leave FAMI. A total of 47% reported that they outgrew the program eligibility age, 40% that they started attending a different ECD public program (12% a parenting program and 28% a childcare program), and 13% reported to have moved to another municipality. In Tables F.2 and F.3 in Online Appendix F, we show that the treatment slightly reduces the probability of dropping out of FAMI for an alternative program and is not related to the probability of attending MF.

If age-eligible, a family could have attended a maximum of 44 weekly group sessions and received 11 monthly home visits during the study period. In terms of effective attendance, 77.5% of all children in the treatment group assessed at follow-up participated in at least one FAMI pedagogical activity (group session or home visit), while the rest did not attend any at all. Information on participation in specific activities was collected as part of the supervision protocol of the enhanced intervention and therefore is only available for the intervention group. In Figure F.1 in Appendix F (graphs (a) and (b)), we show the distribution of children in the intervention group by total exposure to the pedagogical component of the program. Conditional on having attended at least one session, the median number of pedagogical activities attended was 28 out of a total of 55.^21^

On the main reasons why parents found it difficult to attend group sessions or receive home visits, close to 38% reported child illness, 15% reported maternal illness, and 19% reported conflict with other commitments. An additional 12% reported difficulties in finding or being able to afford transportation to the meetings, and 10% reported bad weather. The remainder reported other reasons. Children with lower program attendance were older, less likely to live with their fathers, and had younger and more educated mothers. While they exhibited better learning environments at home, they were exposed to higher verbal or physical punishment (Table F.4 in Online Appendix F).

Regarding, the nutritional component of the intervention, close to 29% of children in the treatment group did not receive any nutritional supplements, and those who received at least one, received 9.8 supplements on average (SD = 3.6) out of a maximum of 14 (Online Appendix F, Figure F.1, graph (c)). As the supplements were delivered by the FAMI mother during the first group meeting of each month, non-attendance implied that a beneficiary might not receive the supplement. We cannot verify if and how the nutritional supplement was used at home or the extent to which it was shared within the family.

Compliance with both components of the program largely overlapped with the same subsamples of children. In particular, 66% of children in the treatment group received at least one nutritional supplement and attended at least one session, 21% did not receive any nutritional supplements nor attend any sessions, 9% attended at least one session but did not receive any nutritional supplements, and 5% received at least one supplement but never attended sessions (Figure F.1, graph (d) in Online Appendix F).

## 4. Estimating Average Impacts

For each outcome of interest, we estimate Intent to Treat (ITT) effects on children’s development using the regression

(1) \begin{equation*} y_{isl,1} = \beta _0 + \beta _1 T_{sl} + \delta ^{\prime } X_{isl,0} + F_{l,0} \sigma + D_0 \theta + Z_{isl,1}\rho + \varepsilon _{isl, 1}, \end{equation*}

where ${Y}_{isl,1}$ is an outcome of interest for child *i* in FAMI unit *s* in town *l* at follow-up (*t* = 1); $T_{sl}$ is a dummy equal to 1 if the FAMI unit *s* in town *l* was in the treatment sample. $X_{isl,0}$ is a set of baseline child and household characteristics, including child’s age, gender, weight-for-age and height-for-age *z*-scores, the household’s wealth index, maternal PPVT scores (to proxy for maternal IQ), and an indicator for the mother being an adolescent. These are included to improve efficiency and to correct for any minor baseline imbalances caused by attrition.^22^ Finally, $D_0$ represents a set of department fixed effects, which control for regional differences, $Z_{isl,1}$ is the vector of tester or interviewer dummies, and $\varepsilon _{isl,1}$ is the residual term. We cluster standard errors of the estimates at the town level, which is the unit of randomization.

The presence of the MF program in the town does not bias our impact estimates. MF was in place before randomization, and our sample of children was drawn from those attending the FAMI center at baseline before randomization. Moreover, as documented in Online Appendix F, treatment did not affect the probability of switching to MF, and it only affected that of switching to other alternatives marginally.

In addition to average impacts, we look at impacts across the distribution of outcomes and also analyze the possibility of heterogeneous impacts in two ways. First, we consider the entire distribution of the outcomes of interest in the treatment and control samples and test for differences in these distributions using the Anderson–Darling statistics (Anderson and Darling 1952).^23^ Second, we re-estimate equation (1) for subgroups in the evaluation sample. In particular, we divide the sample by wealth, as measured by a household wealth index, by the mother’s education, and by the child’s gender.

## 5. The Impact of the Improved FAMI

For most outcomes, we measure impacts in terms of SD units of the variable of interest in the control group. We also include the 95% confidence interval, the standard *p*-value for two-tailed null hypotheses, and the Romano–Wolf stepdown *p*-values adjusted for multiple hypotheses testing for the specific group of hypotheses presented in each table. The Romano–Wolf procedure was performed using 2,500 bootstrap replications and clustering by town.

### 5.1. Main Impacts

In Table 5, we report the average impacts of the intervention on the Bayley-III factor for a summary measure of overall development; the ASQ:SE for socio-emotional development; and the height-for-age *Z*-score for nutritional status. In subsequent tables, we present results for more disaggregated measures of these outcomes. Impacts are computed regardless of whether children actually attended the program or how many times they attended, that is, these are Ordinary Least Squares (OLS) estimates of equation (1) or ITT.

**Table 5.** Impact on children’s outcomes.

	Impact (95% CI)	*p*-value	RW *p*-value
Bayley-III factor	0.163$^{**}$	0.015	0.047
	(0.035, 0.290)		
ASQ:SE total score	0.021	0.722	0.704
	(-0.096, 0.139)		
Height for age *Z*-score	0.078	0.190	0.317
	(-0.038, 0.195)		

Notes. 95% confidence interval in parenthesis for two-tailed tests. Standard errors clustered by town. Covariates included: child’s gender, an indicator of high household wealth index, maternal PPVT score, teenage mother, an indicator of high municipality population, previous attendance to a childcare center, department and interviewer fixed effects, and baseline weight-for-age and height-for-age *Z*-scores. Bayley-III factor is a factor score of the five age-standardized Bayley-III scales. ASQ:SE total score is the age-standardized ASQ:SE score.

$^{**}$ *p* $<$ 0.05 based on Romano–Wolf adjusted *p*-values (RW, Romano and Wolf 2005, 2016), as we consider three simultaneous hypotheses for children’s outcomes.

The effect of the program on the Bayley-III factor was 0.163 SD, and it is statistically significant at the 5%, after adjusting for multiple hypotheses testing for the three primary outcomes in the table. We find no significant average impact of the program on socio-emotional development or height-for-age *Z*-scores. Socio-emotional development is part of the set of potential outcome variables, as the program also aimed at training mothers in sensitive and responsive parenting and appropriate behavior management. However, the curriculum had a stronger focus on cognition and language through the demonstration and practice of specific activities, which might explain the lack of effect on socio-emotional development.^24^ We discuss further the results on nutritional status below.

As mentioned, the impacts in Table 5 are measured in terms of SD of the outcome of interest in the control group. An alternative meaningful metric would be the fraction of the gap in the outcome of interest that the estimated impact represents in a reference population. To perform such an exercise, we use a subsample of children analyzed by Rubio-Codina et al. (2015). The authors considered a sample of about 1,400 children aged 6–36 months living in families representative of the bottom 85% of the wealth distribution in Bogota and estimated a difference in the Bayley-III cognitive scale of about 0.8 SD between those in the top and the bottom 25% of such a wealth distribution, which corresponds roughly to the 17th and the 68th percentile of the entire population in the city. To make the Bogota and the FAMI samples comparable, we estimated a factor model using both samples simultaneously, but limiting the Bogota sample to children of the same age as the FAMI children. We used the Bayley-III cognitive scale, available in both samples, as an anchor and imposed a loading factor normalized to one. We find that the developmental levels of FAMI children are similar to those of children in the bottom 10% of the Bogota sample, and the impact of the intervention is equivalent to closing the gap between children in the top and bottom wealth decile by 23%.

The size of these effects is not negligible, especially if we take into account that the intervention lasted on average no more than 45 weeks and attendance was incomplete (77.5% attended at least one session). It also compares favorably to the impact of nearly 0.26 SD obtained in Attanasio et al. (2014), which was a one-on-one weekly home visiting program that lasted for 18 months with very high compliance rates.

#### The Role of Attrition.

As discussed earlier, there has been some attrition, which is a differential between the treatment and control groups, even conditional on observables. To assess the possible bias caused by this, we estimate a selection model where attrition is a function of baseline characteristics as well as indicators for the identity of the interviewers assigned to households at baseline and follow-up. The identity of the interviewers explains attrition, presumably because of differing quality among them. Furthermore, as interviewers were allocated randomly across towns, making their identity orthogonal to individual characteristics, their identity is a valid instrument. We also need to assume that the identity of the interviewers is unrelated to children’s outcomes, which is reasonable since those administering the Bayley-III test were different people from the interviewers collecting the household survey. The attrition equation is estimated jointly with the outcome equation. The results are reported in Table G.1 in Online Appendix G and show that our conclusions are not sensitive to correcting for such non-random attrition.

### 5.2. ToT and Dosage Effects

#### ToT Effects.

Since non-compliance with the program is one sided, we can use instrumental variables to identify the effect of ToT, using the random assignment to treatment as an instrument. There are, however, many different ways of thinking of the intensity of the program. If we measure effective participation as the fraction of children who attended at least one of the pedagogical activities of the program (i.e. a group session or a home visit), which is 77.5%, then the ToT on the Bayley-III factor is 0.21 SD. If, instead, we measure effective participation as the fraction of children in the treatment group who attended at least the unconditional median number of sessions (i.e. 21 out of 55 total), which is 53.2%, the ToT on the Bayley-III factor is 0.30 SD. Finally, if we define effective participation as the fraction of children who attended the median number of pedagogical activities conditional on having attended at least one (i.e. 28 sessions), which is 38.6%, then the ToT effect is 0.42 SD.^25^ Thus, the potential effects are large even for a reasonably short intervention, delivered in groups. To realize such potential compliance, we would need to improve our understanding of the factors that drive attendance and whether parents misperceive the returns of the program in terms of child development. This is a key area of further research.

#### Dosage Effects.

By the time follow-up data were collected, the FAMI intervention had been running for about 10 months. This short interval was dictated by budgetary considerations. As discussed in Section 2, the intervention involved training the FAMI mothers for about 3.5 weeks. The trainers, divided into several groups, covered all the treatment towns in about 2 months. The end-line data collection itself extended for about 2 months. The combination of these two factors meant that by the time the outcomes were measured the potential intervention dosage that children could be exposed to in the various treatment communities varied considerably, between 34 and 58 weeks. We define the potential dosage of the intervention as the number of sessions that could have been attended during the period comprised between the date in which the children were assessed at end-line and the date on which the training had been completed, divided by 100. For the control sample, dosage is fixed at 0. As this measure of dosage was determined by logistical considerations, it is very likely to be uncorrelated with child development outcomes, and thus, we assumed it is exogenous.

To corroborate this assumption, we test whether dosage correlates with a number of village variables within the treatment group. The results do not show any discernible correlation (see Table F.4 in Online Appendix F). Furthermore, we add to the observable controls in equation (1) a variable that measures the difference in days between follow-up and baseline data collection rounds. This difference was also driven by similar logistic considerations but does not correlate with our measure of dosage.

Given this evidence, we modify equation (1) in the following fashion:

(2) \begin{equation*} Y_{isl,1}= \beta _0 + \beta _1{Dos}_{sl} + \delta ^{\prime } X_{isl,0} + F_{l,0}\sigma + D_0\theta + Z_{isl,1} \rho + \varepsilon _{isl,1} , \end{equation*}

where ${Dos}_{sl}$ is dosage as defined as above. We report the results on the Bayley-III factor as the outcome of interest in Table 6.

**Table 6.** Effects of potential dosage on Bayley-III factor.

	Potential dosage (standard error)	Effect of average potential dosage (*p*-value)
Bayley-III factor	0.209$^{**}$	0.169$^{**}$
	(0.079)	(0.010)

Notes. Standard errors clustered by town. Covariates included: child’s gender, an indicator of high household wealth index, maternal PPVT score, teenage mother, an indicator of high municipality population, previous attendance to a childcare center, department and interviewer fixed effects, baseline weight-for-age and height-for-age *Z*-scores, the difference in days between baseline, and follow-up data collections. In the treatment group the potential dose varies from 34–58 weeks.

$^{**} p < 0.05$ .

The estimates show a positive and significant effect (with a *p*-value of 0.010) of dosage equivalent to an increase of 0.209 SD in cognitive development for every 100 additional sessions. In the last column of the table, we report the impact implied by these results for the average dosage received by children in the treatment group, which is estimated at 0.169. This result is consistent with the impact reported in Table 5. We also experimented with a quadratic specification for dosage. We do not find any significant non-linearity. This result is perhaps not surprising given the relatively short amount of time the intervention had been implemented at the time we collected follow-up data.

### 5.3. Heterogeneous Impacts

In this subsection, we look at heterogeneity in impacts. As mentioned in Section 4, we consider both unobserved heterogeneity and heterogenous impacts of observable variables, such as wealth and maternal education.

#### Unobserved Heterogeneity.

**Figure 1.**
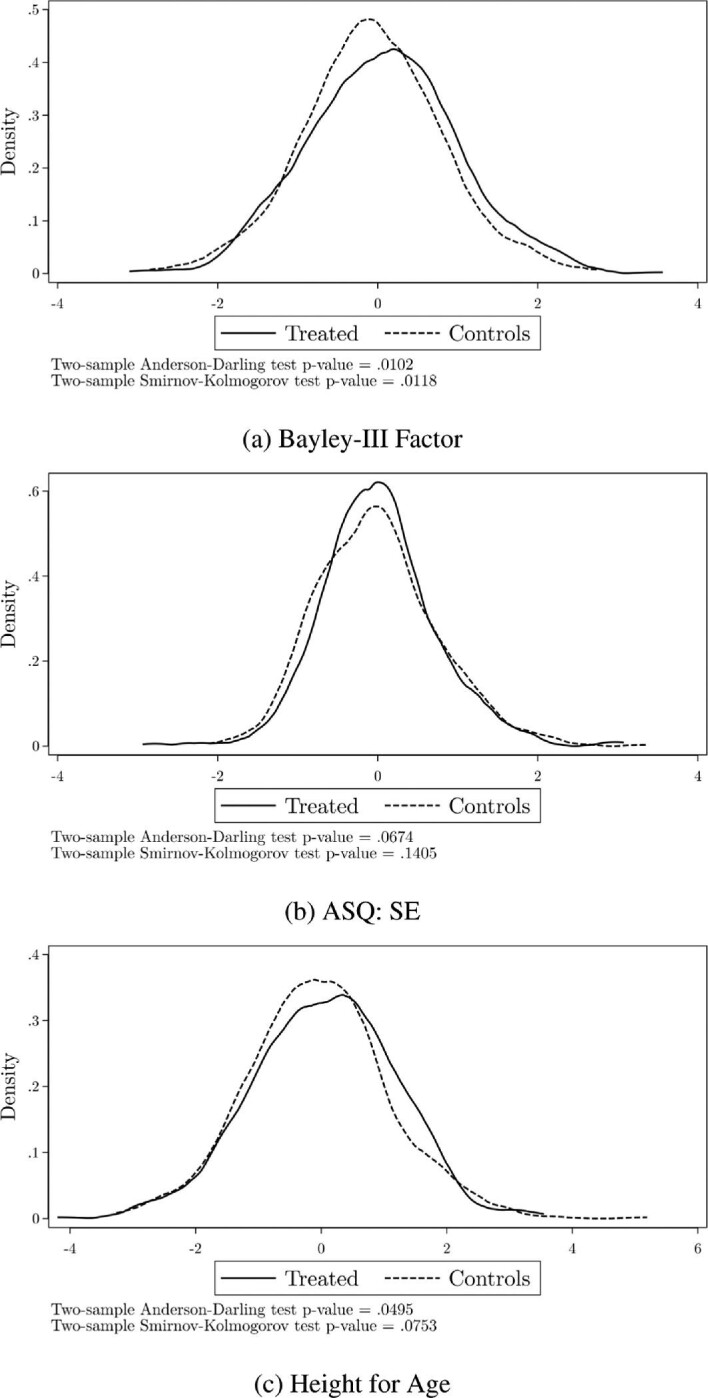
Distribution of conditional outcomes by treatment status. Plot of the distribution of the residuals resulting from a regression of outcomes on observed characteristics described in equation (1), for the treatment and the control samples separately.

Figure 1 reports the distribution of the Bayley-III factor and the ASQ:SE (socio-emotional skills) by treatment and control. To obtain each figure, we first regress the respective outcome on the control variables included in equation (1), and then we plot the distribution of the residuals of this regression for the treatment and the control groups separately. In the graph, we also report the *p*-value of the Anderson–Darling (AD) and the Kolmogorov–Smirnov (KS) tests for the null hypothesis of identical distributions by groups.^26^

What is apparent from the graphs and the results of these tests is that the program had a significant impact on the Bayley-III factor (*p*-values = 0.010 and 0.012 for the AD and KS tests, respectively) and affected the distribution over most of its support. The results for the ASQ:SE are less strong; nevertheless, the *p*-value for the AD test is 0.067, showing some impact.

As we saw in the descriptive analysis, 12% of the children in our sample are stunted (height-for-age $<$ -2 SD) and 15% are at the risk of stunting (-2 SD $<$ height-for-age $<$ -1 SD). It is well-established that stunting at this age is a good indicator of long-term malnutrition and can have long-run negative impacts on human capital development (Hoddinott et al. 2013). The program included a significant nutritional component, which given the nature of our sample, could have both a short- and a long-term impact. While Table 5 did not show significant impacts on height-for-age, the third graph in Figure 1 shows a more nuanced picture and significant impacts on the distribution of height for age (*p*-values = 0.050 and 0.075).

We pursue this in Table 7, where we assess the impacts on different parts of the distribution of height-for-age. The results indicate that the fraction of children whose height-for-age was below -1 SD decreased by 6.8 percentage points or 0.15 SD, while the number of children with normal height-for-age increased by a similar fraction (7.6 percentage points). Both results are statistically significant at the 5% level, even after adjusting the *p*-values for multiple testing, and point to the value of considering the entire distribution. This result is of importance because it has often been proven difficult to impact height-for-age through less intensive interventions (Bernal 2015).

**Table 7.** Impacts on height-for-age by ranges of the distribution.

	Impacts (95% CI)	*p*-value	RW *p*-value
Pr(Height-for-age between –5 SD and –1 SD)	$-0.068^{**}$	0.024	0.044
	($-0.126,-$0.010)		
Pr(Height-for-age between –1 SD and 1 SD)	0.076$^{**}$	0.013	0.033
	(0.017, 0.134)		
Pr(Height-for-age between 1 SD and 5 SD)	-0.001	0.950	0.955
	(-0.025, 0.023)		
Observations	Treatment 559	Control 632	
	559	632	

Notes. Impacts measure the change in the probabilities considered in each row in a linear probability model. Standard errors clustered by town. Covariates: child’s gender, an indicator of high household wealth index, maternal PPVT score, teenage mother, an indicator of high municipality population, previous attendance at a childcare center, department and interviewer fixed effects, and baseline weight-for-age and height-for-age *Z*-scores.

$^{**} {p} < 0.05$ based on Romano–Wolf adjusted *p*-values (RW, Romano and Wolf 2005, 2016), considering all three hypotheses jointly.

#### Observed Heterogeneity.

We now consider how average impacts differed across key groups. This exercise can help us understand whether the intervention helped the most vulnerable and from a policy perspective it helps improve targeting. We investigate whether the effects of the intervention on children’s development, as measured by the Bayley-III factor, varied by maternal education, child gender, and household wealth at baseline.

For each of these three baseline variables, we divided the sample into two groups: less than high school versus more for maternal education; boy versus girl for child’s gender; and household wealth above or below the sample median.^27^ The results are reported in Table 8. Impacts do not seem to substantially vary by the level of maternal education. Although the point estimates are larger for mothers with complete high school (0.176 SD vs. 0.142 SD), this difference is not significant. Turning to gender, the point estimates suggest that the intervention worked better for boys, but the differences are, again, not significantly different from zero. However, we do find significant effects of wealth on the impacts, even after correcting for multiple testing, across all the six hypotheses considered jointly. The effects, at 0.24 SD, are estimated to be much stronger for children living in poorer households. Moreover, the difference between the impact on children from poorer households and that on children from the higher wealth group is significant, with a RW *p*-value of 0.060.

**Table 8.** Heterogeneous impacts on the Bayley-III factor by child and household characteristics at baseline.

Group (${{\mathit N}}$)	Impacts (RW-*p*-value)	Estimated difference (RW-*p*-value)
Maternal education $\ge$ complete high school (*N* = 660)	0.176$^{*}$	0.034
	(0.072)	
Maternal education $<$ complete high school (*N* = 632)	0.142	(0.757)
	(0.234)	
Male (*N* = 673)	0.198$^{*}$	0.074
	(0.077)	
Female (*N* = 619)	0.125	(0.717)
	(0.231)	
Wealth index above the median (*N* = 657)	0.042	−0.243*
	(0.592)	
Wealth index below the median (*N* = 635)	0.285$^{***}$	(0.060)
	(0.008)	

Notes. Heterogeneous effects estimated by subsamples: Difference is a cross-model test for ITT associated parameter. Covariates: child’s gender, an indicator of high household wealth index, maternal PPVT score, teenage mother, an indicator of high municipality population, previous attendance to a childcare center, department and interviewer fixed effects, and baseline weight-for-age and height-for-age *Z*-scores. Romano–Wolf stepdown *p*-values for the six multiple hypotheses for the impact and three hypotheses for the differences in the last column.

$^{*}$ *p* $<$ 0.10, $^{***}$*p*$<$ 0.01 based on Romano–Wolf adjusted *p*-values (RW, Romano and Wolf 2005, 2016).

This result is key and contains both a positive and a negative message: The intervention can indeed improve the outcomes of the most deprived group in this already poor population. However, the better-off children from this group are in no way “well-off” or middle class, and neither do they measure up well in their development against, say, even the Bogota middle class, never mind the international standards. Hence, the intervention would need to improve for this group. These results generally highlight the difficulty with improving ECD programs for broad populations, so targeting interventions to the needs of separate groups is likely to be important. No significant heterogeneous effects were found in the case of socio-emotional or nutritional outcomes.

Lastly, we investigate whether intervention impacts varied by quality of implementation and FAMI mother characteristics. We do not find any significant differences in impacts by any of the measures of implementation fidelity available, nor by FAMI mother’s age or education. The only variable for which we find some marginally significant differences in impact is a measure of FAMI mother’s “motivation”, as assessed by the tutors: Children who attended centers by a FAMI mother reported to be more “motivated” than the median, registered a higher impact (0.22SD vs. 0.07). This 0.15 difference is significant with a *p*-value of 0.099.

## 6. Understanding the Impacts

In this section, we study possible mechanisms that could have generated the documented impacts on final outcomes. We start by estimating the impact of the intervention on a number of inputs that are relevant for child development, following Heckman, Pinto, and Savelyev (2013). We then take a structural approach to estimate the causal link between the relevant inputs we consider and child development, taking into account the possible endogeneity of the former, through a production function framework similar to that in Cunha and Heckman (2008), Cunha, Heckman, and Schennach (2010), and Attanasio et al. (2020).

### 6.1. Effects on Intermediate Outcomes and Mediation Analysis

The intervention we are studying is a transfer in kind of early education and nutritional supplementation. As with other transfers in kind, the intervention can induce parents to change their contributions to their child’s development in other dimensions. The food supplement delivered by the intervention we are evaluating could be clawed back by reducing other food inputs to the target child, or perhaps sharing it in the family and even selling it; and the additional stimulation received by the target children could cause parents to switch attention to other children or to themselves, therefore mitigating the intervention’s impact. On the other hand, it is also possible that low-income parents are not fully aware of the returns to investing in their children (Cunha, Elo, and Culhane 2013; Attanasio, Cunha, and Jervis 2019), so that the effects of the intervention may have been generated by an increase in investment induced by a change in these beliefs. Therefore, there are also good reasons to believe that, instead of crowding out, the intervention could have led to a crowding in of resources. In this case, adding to the transfer from the intervention may have particularly high returns. Indeed, Attanasio et al. (2020) evaluates another early years stimulation intervention in Colombia and shows that, in response to it, parents crowd-in resources by increasing investments. Exploring the mediating factors and the mechanisms underlying intervention impacts is a way of obtaining answers to some of these questions. Moreover, understanding these is critical to improve the design and targeting of public policies.

We start by presenting, in Table 9, the effects of the program on the intermediate outcomes described in Section 3.1. The first row reports the impact of the intervention on parental investment, estimated from the FCI index, which captures the quality of the home environment, combines books, magazines and newspapers, play activities, and play materials in the home (see Online Appendix D). The following rows assess impacts on maternal knowledge about child development, maternal self-efficacy, and food insecurity. Maternal knowledge and self-efficacy as potential mediators capture the idea that, through the intervention, parents (mothers, in particular) might become more effective in their childrearing practices.

**Table 9.** Program impacts on intermediate outcomes.

	Impact as fraction of SD in control group (95% CI)	*p*-value	RW *p*-value
Parental investment	0.340$^{***}$	0.000	0.000
	(0.207, 0.472)		
Maternal knowledge (raw score)	-0.016	0.831	0.828
	(-0.160, 0.128)		
Maternal self-efficacy (raw score)	0.039	0.604	0.828
	(-0.108, 0.186)		
ELCSA food insecurity status	-0.089	0.220	0.496
	(-0.231, 0.052)		

Notes: $^{*} p < 0.10$, $^{**} p < 0.05$, $^{***} p < 0.01$ based on Romano-Wolf adjusted p-values (RW, Romano and Wolf 2005, 2016), considering all four hypotheses jointly. 95% confidence interval in parenthesis for two-tailed tests. OLS estimation; standard errors clustered by town. Impacts are measured in terms of SD of the control group. Covariates: child’s gender, an indicator of high household wealth index, maternal PPVT score, teenage mother, an indicator of high municipality population, previous attendance to a childcare center, and department and interviewer fixed effects. Parental investment is measured by a factor model estimated using the subscales of FCI Home Environment Quality, as discussed in Online Appendix D.

The impact on the quality of the home environment was 0.34 of a SD in the control group and statistically significant, with a *p*-value of zero. This is a strong result and indicates that the intervention induces parents to invest more in their children. However, we do not find any statistically significant program effects on maternal knowledge about child development, maternal self-efficacy, or food insecurity.^28^

### 6.2. A Structural Interpretation of the Impacts: Production Function Estimates

Given the results on intermediate outcomes, we proceed to estimate a model where child development is determined by a production function, which depends on parental investment and other background variables. Both child development and parental inputs are represented by latent variables, which are not observed directly but for which we have informative markers that allow us to estimate them by factor analysis. Given the evidence in Table 10, the sole mediator we consider for child development is parental investment. This approach is a similar to that of Heckman, Pinto, and Savelyev (2013). However, here, following Attanasio et al. (2020), we also consider the possible endogeneity of parental investments.

**Table 10.** IV estimation of the production function for Bayley-III factor.

	OLS		First stage	IV	
	Bayley-III factor		Parental investment	Bayley-III factor	
	(1)	(2)	(3)	(4)	(5)
Treatment (T)	0.135$^{**}$	0.079	0.294$^{***}$	0.006	
	(0.065)	(0.065)	(0.068)	(0.110)	
Parental investment (PI)		0.185$^{***}$		0.467$^{*}$	0.454$^{***}$
		(0.036)		(0.249)	(0.171)
Time to town hall	-0.099$^{***}$	-0.079$^{***}$	-0.040	-0.048	-0.049
	(0.027)	(0.028)	(0.030)	(0.043)	(0.037)
Time to FAMI			-0.143$^{***}$		
			(0.035)		
First stage *F*-statistics					
IV: time to FAMI			16.86		
IV: time to FAMI and treatment			19.15		
Overidentification *p*-value					0.956
*N*	1,292	1,292	1,292	1,292	1,292

Notes. Standard errors are clustered by town in parenthesis. Covariates: child’s gender, an indicator of high household wealth index, maternal PPVT score, teenage mother, an indicator of high municipality population, previous attendance at a childcare center, department and interviewer fixed effects, and baseline weight-for-age and height-for-age *Z*-scores.

$^{*} p < 0.10$ , $^{**} p < 0.05$, $^{***} p < 0.01$.

We estimate a production function for human capital development, which we assume to be a function of parental investment, several other environmental factors, and, potentially, the intervention itself. In particular, we assume that child development can be expressed by the Cobb–Douglas production function:

(3) \begin{equation*} \ln ({\textit {CD}}_{isl}) = \gamma _0 + \gamma _1 \ln (\textit {PI}_{isl}) + \gamma _2 T_{sl} + \delta ^{\prime } X_{isl} + F_l \sigma + D \theta + Z_{isl} \rho + u_{isl}, \end{equation*}

where ${\textit {CD}}_{isl}$ is the child development latent variable and ${\textit {PI}}_{isl}$ represents the parental investments latent variable, both estimated by the factor model described in Online Appendix D and used to estimate the reduced form impacts in Tables 5 and 10. In equation (3), the treatment allocation $T_{sl}$ can affect child development both directly and through its impact on parental investments (PI). The covariates $X_{isl}$ include the child’s gender, household wealth, maternal PPVT score, a dummy variable for teenage mothers, and distance to the municipality’s Town Hall to capture unobserved differences in household socio-economic condition. We also control for baseline childcare attendance and municipal population. Earlier studies also controlled for lagged child development. However, as explained, we did not collect baseline developmental outcomes since the children were too young to obtain a precise measure with the resources we had available. Instead, we control for the child’s nutritional status at baseline’namely, height-for-age and weight-for-age. As before, *D* represents department fixed effects, and $Z_{isl}$ is the vector of tester fixed effects. Finally, $u_{isl}$ represents unobservable factors determining child development, including shocks experienced by the child and additional inputs not observed by the researchers but possibly chosen by parents. The Cobb–Douglas assumption is consistent with the evidence in Cunha, Heckman, and Schennach (2010) and in Attanasio et al. (2020), who performed a similar analysis on another early stimulation intervention in Colombia delivered through home visits rather than group sessions.

The main challenge in estimating the parameters in equation (3) is the fact that parental investment ${\textit {PI}}_{isl}$ is likely to be endogenous, as the parents might be reacting to shocks experienced by the child or might choose investment jointly with other inputs. While the treatment is exogenous by construction, since it is assigned randomly across communities, it is not necessarily a valid exclusion restriction because it can have an independent effect on the outcome. Indeed, a question we pose is whether the treatment affects child development directly or whether its impact is mediated by parental investment. To answer this question, we need to establish the causal link between investment and child development. We therefore need an instrument, $W_{isl}$, that affects parental investment while not affecting child development directly. For this purpose, we use the travel time from the household residence to the FAMI center. To control for differences between households that are centrally located versus households that live in more outlying areas (that could differ in unobservable dimensions), we control for distance to the Town Hall when estimating equation (3) by instrumental variables (IV). Therefore, we estimate a first-stage investment equation of the form:

(4) \begin{equation*} \ln (\textit {PI}_{isl})=\pi _0 + \pi _1T_s + \pi _2W_{isl} + \gamma ^{\prime } X_{is} + v_{is}, \end{equation*}

where the covariates $X_{is}$ are the same as those in the production function in equation (3).

In the first column of Table 10, we report the treatment effect on the Bayley-III factor, estimated by OLS, and in the second column, we introduce parental investment, also using OLS. The coefficient on treatment is reduced in size, and it is no longer statistically different from zero, demonstrating that if the OLS assumption is valid, then the impact is mediated by parental investments (although we cannot necessarily ignore the coefficient on treatment because it is quite large, albeit imprecisely estimated).

In the third column of Table 10, we report the estimates of the investment equation coefficients $\pi _1$ associated with treatment allocation and $\pi _2$ associated with travel time to FAMI, which serves as an instrument when we estimate the production function shown in the subsequent columns. This is strongly significant, even conditional on distance to the Town Hall, which is intended to capture how centrally the household is located. Importantly the *F*-statistic is large enough to rule out a weak instrument problem, whether treatment is used an additional exclusion restriction or not (see bottom of column (3)).

In the fourth column of Table 10, we re-estimate the production function, as in column (2) but using IV. These estimates show a much higher impact of investments and a zero direct effect of treatment: The point estimates imply that the entire effect of treatment is driven by an increase in parental investments through the intervention. The difference between the investment coefficients in columns (2) and (4) from 0.185 to 0.467 is significant at the 10% level and consistent with the results reported in Attanasio et al. (2020), where the coefficient in the production function of child development also increased considerably after accounting for the endogeneity of parental investment. This suggests that parents are compensating for negative shocks when choosing an investment.

Given this last consideration, in the fifth column of Table 10, we remove the intervention from the production function (3). Now the coefficient on investment is 0.454, and it is significant at the 1% level. We notice that the model is now overidentified, as we now have two instruments for the single endogenous variable, ${\textit {PI}}_{is}$. When testing the implied overidentifying restriction, we do not reject the null of the correct specification.

## 7. Discussion and Conclusions

Interventions that promote ECD, starting from birth, may well be the key to successful human capital policies, particularly in poor environments. However, the characteristics and effectiveness of such programs at scale are not well understood yet. In recent years, many early years interventions have been implemented worldwide, but effective and sustainable programs at scale are rare. Furthermore, many institutionalized initiatives are of low quality (Lo, Das, and Horton 2016). Scaling up is not only a question of funds, but also of the available human resources in a variety of different contexts. A possible approach to deploying early years intervention at scale is to determine whether existing large-scale programs (and their infrastructure) can be successfully improved, so to guarantee the quality required for them to have significant impacts on children.

In this study, we present results from an experiment where we designed and implemented a scalable intervention that was added to an existing government group-based parenting support intervention, combined with nutritional supplementation. Effectively, the intervention we study is an improvement of an existing national program, consisting of incorporating structured content (curriculum of activities) and training and coaching for program facilitators, as well as nutrition education and a larger and higher quality nutritional supplement. As we have discussed, this design offers a directly scalable policy, both in terms of its costs and in its implementability, given the existing infrastructure and human resources. We should stress that we are not evaluating the impact of FAMI as it exists or of our intervention compared to a situation with no program. As we have mentioned, FAMI has existed for many years, and a direct evaluation of it does not exist and would be difficult, if not impossible, to perform. On the other hand, we think that our exercise is useful and relevant for the current policy debate, which is considering improvements and not the abolition of FAMI.

Our curriculum is an adaptation of RU, a home visitation program shown to be effective in altering the long-run cognitive trajectory of children from deprived environments in its original implementation in Jamaica (Walker et al. 2011; Gertler et al. 2014). Adaptations of the curriculum to a variety of contexts and countries have also had positive impacts on developmental outcomes (see Grantham-McGregor and Smith (2016) for a review).

Evaluation of group-based adaptations of RU and other parenting programs is, however, more limited. Yet, they represent a promising and natural low-cost approach to improving outcomes in vulnerable populations in a more efficient manner as delivery is less intensive in human resources. Furthermore, while the delivery of the RU curriculum in groups might imply a reduced focus on the specific needs of an individual child, well-run groups might induce positive effects by improving existing networks and acquaintances and provide role models for some mothers.

The fact that we find reasonably-sized positive impacts in the short time span covered by our data collections is important, in practice, the intervention would last longer, and children would hopefully graduate into pre-schools where they could gradually build up their abilities and school readiness, thus addressing one key cause of poverty persistence. The evidence we present also points to potentially large gains where they are most needed, namely, among the poorest. The importance of these results is even more apparent if we consider the fact that compliance with the number of sessions actually attended by children and their caregivers was relatively low and the intervention was relatively short, at least in comparison with the most successful efficacy trials referred to in this study. And yet our intervention had an ITT effect of 16% of a SD and a ToT effect of up to 42% of a SD in development. Moreover, there was a reduction in the fraction of children whose height-for-age was below -1 SD of 5.8 percentage points.

Some features of this particular study make us believe that these estimates are lower bounds of the potential of this intervention. First, the control group had access to the basic program, without the improved intervention, unlike similar studies in the literature in which the control group did not receive any intervention. Second, as stressed, the average impact reflects larger impacts for the children most in need and a small or null impact for the better-off children. Third, and most importantly, it was not possible to fully control and enforce the many relevant implementation aspects that might be needed to ensure fidelity of the intervention and impact development.^29^ In fact, the implementation of the intervention was far from smooth and faced various challenges. Examples of the problems encountered include the low duration of participant exposure to the program, logistical difficulties for the delivery of pedagogical materials and the nutritional supplements in complicated rural geographies, heterogeneity in the fidelity of program implementation, and initial resistance of program providers to change their behavior. The implementation problems we document in our context are common to many programs implemented at scale.

The focus on the scalability is one of the most salient aspects of this study and reflects the difficulties policy makers face when moving from small trials to larger studies with reduced control over what actually happens in the field. As we suggest above, when an intervention is scaled-up, one needs to consider not only financial costs but also the possibility of sustaining the quality of implementation given the existing service infrastructure. On the latter, we notice that our intervention was implemented on top an existing program, with a minimal involvement on the part of the researcher team. Our results indicate that despite a number of implementation problems, which were in part present because we wanted to work with a model that could be reproduced at scale, the enhancement we evaluated had a sizeable effect on the children most in need. However, we do recognize that it is not obvious that a scaled-up intervention could maintain the level and quality of training and mentoring that were achieved during the study, although we stress that the evaluation did not use personnel with special qualifications. In any case, it is clear that proper mentoring should be developed with care.

Regarding the financial cost of the intervention, we notice that the cost of the pedagogical component of the intervention was ${\$}$115 US per child per year (${\$}$27 US for pedagogical materials and ${\$}$88 US for coaching) plus a ${\$}$11 US one-off cost per child for FAMI pre-service training. At scale, there could be important economies of scale in the mentoring system, by far the largest component of the total pedagogical cost, which could reduce these figures substantially. The cost of the additional nutritional supplementation was ${\$}$209 US per child per year. By the end of this study, the Colombian government adopted the nutritional supplementation evaluated herein nationwide, with an investment of ${\$}$10 US million. The pedagogical component corresponds to 40% of the operational cost of the unenhanced version of the FAMI program, equivalent to 1.7 monthly minimum wages per year. In contrast, center-based childcare services cost ${\$}$1,100 US per child per year. Or the transition to large childcare centers, which has been one of the center pieces of recent government policy, costs ${\$}$780 US per child per year, more than twice the intervention we are studying. Therefore, the cost of our intervention is moderate, especially, in comparison to other ECD programs in the country, and financially sustainable.

As we stressed above, the impacts of the intervention we evaluated are relative to a status quo where children of the same age were receiving an unimproved program. To interpret these results, it is useful to put them in the context of the quality of other public early-years services in Colombia. Bernal (2013) presents a diagnostic of public childcare quality by modality, using standard measures. Quality levels are low for all modalities, close to minimum standards. This pattern is also found in other Latin-American countries. Part of the problem is precisely the lack of a structured curriculum and supervision/mentoring strategies, which is what the improvement we evaluate introduces to FAMI. What we show is that scaling up services with quality is possible within an existing institutional infrastructure that allows for such coaching and mentoring strategies. The evidence we presented suggests that it is possible to gradually improve the quality of nationwide programs at scale in a way that is affordable. Ours is an enhancement of an existing program that leverages on local low-skilled human resources. Critically, the intervention specifically aims at improving process quality (such as the integration of a structured curriculum and improved interactions between caregivers and children supported by coaching and mentoring), which the literature has shown to be critically associated with child developmental outcomes (Yoshikawa, Weiland, and Brooks-Gunn 2016).

A key question is whether these short-term impacts sustain over time. Andrew et al. (2018) reports that the effects on child development and parental investment documented in Attanasio et al. (2014) disappear two years after the end of the intervention. The authors mention that this result might be due to a small initial effect (similar to ours) and/or the lack of continued family support for early stimulation. The impact fade-out observed for the intervention studied by Attanasio et al. (2014) is not unique. Several studies have found that medium-term program impacts might vanish but reappear later in the child’s life-cycle (Lawrence et al. 2005).

In Attanasio et al. (2014), intervention activities ended as soon as the study ended. In our case, however, the intervention effectively kept running since an important part of it consisted of the training of the facilitators in the pre-existing program. In particular, most treated FAMI providers continued to use the curriculum even though they were no longer being coached. In addition, participants in public programs are more likely to continue to be enrolled in similar public programs as children grow. For example, children could have moved on to home-based childcare, provided through the Hogares Comunitarios program (Bernal and Fernández 2013), which could help reinforce or maintain these effects over time.

The total number of FAMI beneficiaries has decreased since 2013. However, close to 150,000 children are still part of this program. Crucially, the toolkit developed for this intervention is flexible and easily adaptable to any ECD programs facilitated by paraprofessional personnel, as many are in Colombia, as well as in other developing countries. As we discuss in detail in Online Appendix B, it would be straightforward to replicate at scale the training and coaching strategy proposed in this study by leveraging on the already existing monitoring and supervision infrastructure for community-based programs, including FAMI. Training professional staff in local ICBF offices would be feasible, and they could easily implement both training and coaching of FAMI and similar programs run by paraprofessional personnel.

While the pre-existing program is present everywhere in Colombia, we implemented and evaluated the improvement in Central Colombia. This choice was motivated by the fact that this region tends to be more culturally and ethnically homogeneous with respect to other parts of Colombia, such as the coastal regions (both Pacific and Atlantic) where Afro-Colombians and indigenous) are more likely to reside. Scale up in these regions would likely require additional piloting and adaptation.

To conclude, we show that a scalable program can have substantial effects on child development in highly deprived populations at a low cost and based on government infrastructure. Improving the quality of large-scale programs in developing countries can form a key element of the policy toolkit for fighting poverty.

## Supplementary Material

jvac005_Attanasio_etal_FAMI_Online_Appendix

jvac005_Attanasio_etal_Replication-Data-Code

## Footnotes

1. Cunha et al. (2006), Heckman (2006), Engle et al. (2007), Doyle et al. (2009), Almond and Currie (2011), Pongcharoen et al. (2012), Shonkoff et al. (2012), and Yoshikawa et al. (2013).

2. Moran et al. (2004) and Nowak and Heinrichs (2008) reported that the most effective parenting programs included an evidence-based curriculum, systematic training of frontline workers, and opportunities for parents to learn and practice with children.

3. The *p*-values we report are adjusted for multiple testing as explained in the main body of the paper.

4. Such as the integration of a structured curriculum and improved interactions between caregivers and children supported by coaching and mentoring.

5. Examples from the United States include the Nurse-Family Partnership (NFP) (Olds et al. 1986a,b, 1994; Heckman et al. 2017) and the Promising Practices (Brooks-Gunn et al. 1994; McCormick et al. 2006). In LIMCs, there is the well-known Jamaica home visiting model, which provided early stimulation (play-based activities) and nutritional supplementation (powdered milk) to stunted children in slums in Kingston for 24 months and obtained large impacts on ECD outcomes in the short term that translated into improved IQ and mental health (Walker et al. 2011) and higher wages (Gertler et al. 2014) in adulthood.

6. This approach has applied to all public ECD services in the country to date. The Board for Early Childhood has emphasized the principle of curricular freedom, and national standards are intentionally broad. Program providers are expected to adapt the learning standards to their own programs.

7. While we received authorization from the ICBF to implement and evaluate the intervention, its deployment was not publicized.

8. This was done in two stages: an initial stage of 2 weeks and a second stage of 1.5 weeks about 2 months later, on average. More specifically, towns with less than five FAMI units received 75 hours of training in 3 weeks, towns with six–nine FAMI units were trained for 100–125 hours in 5–6 weeks, and towns with more than ten FAMI units received training during 150–175 hours offered during 6–7 weeks.

9. Specific differences with respect to how they had typically worked were: (i) practicing play activities with mothers and their children; (ii) practicing language activities with babies; (iii) making home-made toys with mothers; (iv) encouraging parents to play with their children at home; and (v) listening to parents about their achievements at home. Almost all of them (99%) reported that they would continue to use the proposed curriculum after the end of the project.

10. In fact, it included more than that as it allowed for a potential consumption of up to 20% of the supplement’s nutritional content by other household members.

11. The package contained tuna, sardines, canola oil, iron-fortified whole milk (the only micronutrient included), beans, and lentils.

12. We could not evaluate the stimulation component alone, that is, without the nutritional component, because both were part of the original program. Dissociating them for evaluation purposes was not feasible, both logistically and ethically.

13. This is conservative, given an average of 9.5 children younger than two per FAMI unit in the sample (plus 2.1 pregnant women); and a nationwide average of 13 (SD = 1.4; range = [10, 24]), as computed using administrative data for 2013 (before the intervention started).

14. While a similar argument on durability could be made for the materials, experience has taught us that their depreciation rate is quite high as they are rotated among families. Hence, it is safe to assume that they do need to be replaced, approximately, on a yearly basis.

15. The trial registration is at http://www.isrctn.com/ISRCTN93757590.

16. This requirement is associated with the power calculations for the trial and to facilitate the logistics associated with the training and coaching carried out by the tutors, who had to travel across various towns.

17. MF is similar to FAMI in that it serves beneficiaries through monthly home visits and weekly group meetings, but (1) it serves children 0–5 years of age, while FAMI serves children aged 0–2; (2) it has a set-up infrastructure for group meetings (a center), while FAMI uses other community spaces or FAMI’s own home; (3) it serves, on average, 45 beneficiaries as compared to close to 15 in FAMI; (4) it is led by a professional and an assistant, as compared to a single person who is not required to have a college degree in FAMI; (5) it offers a nutritional supplement five times larger than that of FAMI; and (6) it has access to a group of professionals including a psychologist and a nutritionist who support MF activities.

18. Child development assessments and anthropometric measures were collected by testers with degrees in psychology and health, respectively. The remaining variables in the household survey were collected by regular enumerators prior to the child assessments.

19. Specifically, we excluded 12 children who scored more than 3 SD below the mean on the Bayley-III cognitive scale (possible disability) and 15 children who were 6 SD below the mean and 6 SD above the mean of height-for-age (extreme observations).

20. These variables are not shown in Table 1 but correspond to the components of the FCI “parental investment” factor.

21. Some other children in the treatment group might have dropped out of the FAMI program between baseline and the beginning of the intervention due to the time elapsed to complete the training of the FAMI mother (up to 4 months). These children, therefore, would not have attended any of the enhanced sessions.

22. Item non-response in baseline covariates is not correlated with treatment status. Thus, we imputed missing covariate values with the average of the non-missing observations and accounted for this imputation with a dummy variable in equation (1). The exact fraction of imputed observations varies by covariate up to a maximum of 6.8%.

23. Such a test is considered more powerful to detect differences in the tails of the distribution than the Kolmogorov–Smirnoff test (Engmann and Cousineau 2011).

24. Note also that the measures used to capture socio-emotional development might not be very precise.

25. There is an additional complication in estimating ToT effects from the ITT impacts we report. As we mentioned above, our estimate represents the impact of the improved FAMI relative to the standard FAMI (status quo), which is attended by the children in the control group. Presumably, there are also compliance problems in the control program on which, unfortunately, we do not have data. The ToT estimate we have discussed should be interpreted as the impact of a fully compliant improved FAMI over the business-as-usual FAMI in which compliance does not change.

26. The Anderson–Darling test focuses more on the tails of the distribution and has been shown to have greater power than alternative tests, such as the Kolmogorov–Smirnov test (Bennett 2008), which focuses on first-order dominance.

27. The wealth index is computed as the first principal component of a number of dwelling characteristics (such as the material of walls, floors, and roofs, the number of bathrooms and rooms, access to utilities, etc.), and durable goods ownership.

28. The effect we found is not as strong as that reported in Attanasio et al. (2014) of 0.5 SD on play materials and play activities with adults at home and resulting from a home visiting intervention in Colombia. We return to this issue in the Discussion section.

29. FAMI providers continued to be paid and supervised by the government with no legal obligation or additional monetary incentive to participate in our program. They were strongly encouraged to do so, but they could choose not to without any practical consequence.

## Notes

*The editor in charge of this paper was Paola Giuliano*.
