# Supplementary material for: Early Stimulation and Nutrition: The Impacts of a Scalable Intervention
Source: J Eur Econ Assoc. 2022 Jan 28;20(4):1395–432. doi: 10.1093/jeea/jvac005 (PMC9372035; doi:10.1093/jeea/jvac005)
Supplement: jvac005_Attanasio_etal_FAMI_Online_Appendix [file jvac005_attanasio_etal_fami_online_appendix.pdf]

ONLINE APPENDICESEARLY STIMULATION AND NUTRITION: THE IMPACTS OF A SCALABLE INTERVENTION

Orazio Attanasio
Yale, IFS, and FAIR at NHH

Helen Baker-Henningham
Bangor University and University of the West Indies

Raquel Bernal
Universidad de los Andes and CEDE

Costas Meghir
Yale, JPal, and IFS

Diana Pineda
Fundación Éxito

Marta Rubio-Codina
Inter-American Development Bank

Appendix A: Detailed Description of the Interventions

Our program was aimed at improving the FAMI government program. During our initial formative research stage, we observed several group sessions in FAMI units located in municipalities not in our c-RCT sample. These were mostly lecture-like meetings in which the FAMI program facilitator would discuss a certain topic with beneficiary mothers sitting around a table and typically aided with a flipchart and/or other visual aids. Most importantly, babies would remain in their strollers or sitting on the mothers' laps with no direct participation in the sessions. The topics covered varied and some examples during our visits included community support, friendship, breastfeeding, dental care and teenage pregnancy, although sessions around sensitive parenting, child development and engagement of parents and other family members were also supposed to be addressed. In spite of such a wide spectrum of topics, however, the existing program did not use any curriculum: what to cover and how was entirely up to the FAMI facilitator.

The curriculum we introduced aims at assisting mothers to provide developmentally appropriate activities for their children (in particular, activities that promote language, cognitive, and motor development), as well as reinforcing maternal knowledge and practices about feeding and nutrition. In doing so, it aims at improving mothers' knowledge, practices, enjoyment in child up-bringing, and self-esteem. Given that the FAMI program is delivered mostly through group meetings and home visits, the

E-mail: orazio.attanasio@yale.edu (Attanasio); h.henningham@bangor.ac.uk (Baker-Henningham); rbernal@uniandes.edu.co (Bernal); c.meghir@yale.edu (Meghir); Diana.PinedaRuiz@grupo-exito.com (Pineda); martarubio@iadb.org (Rubio-Codina)

intervention we designed included two complementary curricula. Both used similar components, actions, and activities to promote better maternal child rearing practices. These included making the mother the agent of change and empowering her to improve her child's development by demonstrating the use of age-appropriate play materials and activities, by providing opportunities to practice with them, and by providing supportive feedback. The program also aimed at training mothers in sensitive and responsive parenting and appropriate behavior management, and encouraged positive mother-child interactions and child maltreatment prevention.

Most of the program content was delivered through the group meetings as they were held on a weekly basis. In addition to being spaces where to demonstrate and practice the use of age-appropriate play materials and language activities, the groups provided opportunities for discussing and practicing effective child rearing skills and positive interactions with children with other caregivers, sharing experiences, group problem-solving, as well as opportunities for social support. Group meetings also provided the opportunity for mothers to discuss how play activities promoted children's development and show them how to make simple toys so that each family could set up a toy library for home use. Group meetings were one hour long. An average of 5 mothers attended each session (min=1, max=15, SD=2.6).

The home visits were delivered monthly and provided the opportunity to introduce activities that were more difficult (i.e., more specific to a child's developmental level) in the context of the group (such as puzzles and matching activities), additional language activities, and specific ideas on how to use routine home activities to promote child development and how to identify materials in the home that could be used to promote child development. Home visits were, on average, one hour long.

Separate group meetings were offered for pregnant and lactating women with children up to 6 months, mothers with children 6-11 months, and mothers with children aged 1-2 years, and mothers were asked to attend the meeting that corresponded to her child's age. However, in practice, this did not always occur, and, in anticipation, the curriculum had been designed so that it could be delivered to groups with children over the entire age range. To this end, the play and language activities were divided into age bands (birth-5 months, 6-11 months, and 1-2 years) by level of difficulty. We expected mothers of children 6-24 months to attend four meetings per month and pregnant and lactating women with children up to 6 months to attend two meetings per month.

Each group session was structured in six different moments: arrival and free play; feedback from the previous group session (10 minutes); song (5 minutes); demonstration and practice of the age-appropriate play and language activities for the week with materials that will be taken home (30 minutes); discussion around a parenting theme or activity (15 minutes); review of the session to ensure that mothers understand the activities, and commitment to practice with children at home (10 minutes); and in closing, they share a snack. The themes for discussion during the group meetings included issues such as the importance of spending time playing with the child, praising the child, talking to the child, things to do at bath time or mealtimes, learning to trust, understanding the child's feelings, teaching the baby about her environment, and child behavior.

Similarly, each home visit consisted of (i) greeting and discussion of any issues, (ii) feedback from the previous home visiting session, (iii) song, (iv) introduction of new play and language activities (including how to integrate into everyday routines, (v) nutrition message, and (vi) a review of activities to be conducted over the next month.

The curriculum included discussion topics or key parenting messages, age-appropriate activities to promote child development using the play materials, as well as everyday activities to encourage adult-child interactions. It was specifically designed to increase the focus on language development with respect to the original RU curriculum. For example, (1) the language activities were designed to be more structured and to involve demonstration and practice, in the same way as the other play activities, rather than being based on discussion, and (2) we included the use of materials in the language activities to make them more concrete (i.e., when encouraging mothers to label and talk about things in the home environment, we used relevant objects, such as a cup, comb, chair, during the session for demonstration and practice). It was very rich in play materials—books, pictures to talk about, home-made toys, puzzles and building blocks—which were used during home visits and in the group meeting. It also included a set of nutrition cards relevant to the children's ages that were discussed with the mother during each home visit. The complete kit of materials had a cost of $27 US per child per year.[1]

In addition to the set of activities and materials, the qualification of the FAMI program also included a training and coaching component (pre- and in-service training) to support and maintain the quality of home visits and group meetings. Shifting away from a supervision model, the new approach consisted of a team of tutors with degrees in psychology and social work, who provided the initial pre-service training and then continued to provide in-service training and support during the implementation period. Tutors, trained and supervised by the research team, were in charge of training FAMI mothers. Pre-service training was provided sequentially by town, where all FAMI mothers were trained simultaneously. Average training time was of 3.5 weeks and 85 hours, although total training time varied by town depending on the number of FAMI units. Specifically, towns with less than 5 FAMI units received 75 hours of training in 3 weeks; towns with 6 to 9 FAMI units received 100-125 hours for 5-6 weeks; and towns with more than 10 FAMI units received 150-175 hours of training over 6-7 weeks. The training involved demonstration, practice and feedback in running the group sessions and conducting the play and language activities with mothers and children, and toy-making. The one-time cost of the pre-service training per FAMI provider was of about $113 US or $11 US per child.

Tutors also coached FAMI mothers continuously throughout the duration of the intervention. In each supervisory round, they observed one group session and one home visit, and provided feedback to the FAMI mother. Each tutor was in charge of 5 towns and 19 FAMI mothers, on average, and hence met with a FAMI mother every 6 weeks

1. All costs are computed at the average exchange rate of 2015-2016 (the evaluation period), of 2,800 COP/USD.

on average. Whenever possible, they also facilitated a group meeting of FAMI mothers in each town to discuss and share positive experiences and challenges and engage in problem-solving. The tutors were supervised by an intervention supervisor—the fieldwork manager, a member of the research team—who conducted visits with each tutor every 2 months.

Training costs included: tutors' salaries (plus fringe benefits), the fieldwork manager's salary, traveling expenses of tutors and supervisor, the cost of the tutors' training by the research team (accommodation, transportation, food, trainer's traveling expenses and accommodation, materials), and incentives offered to FAMI mothers for their participation ($14 US per FAMI). This was costed for 5 months, which was the total duration of training (for both, trainers and FAMI mothers). Intervention fixed costs, such as this one, were amortized over 171 treated FAMI units and assuming an average FAMI size of 10 children. Mentoring/coaching costs were similar to training costs and included: tutors' salaries, the fieldwork manager's salary, and travel expenses for both, tutors and supervisor. The difference is that mentoring is a recurrent cost for every month in which it took place. Overall, the cost of coaching was $82 US per month per FAMI provider or $8 US per child per month. Coaching is provided during 11 of the 12 months of the year which amounts to a total cost of $88 US per child per year.

In addition to the introduction of the early stimulation curriculum, the intervention also included a nutritional component, which comprised the delivery of a monthly nutritional supplement to FAMI participants, and psychoeducation around feeding and nutrition during group meetings and home visits. The nutritional supplement corresponded to 35% of daily calorie intake requirements for pregnant women, breastfeeding mothers, and children younger than 2 years of age (for 30 days). The cost of the package is $26 US per month including shipping costs. It contains tuna, sardines, canola oil, iron-fortified whole milk, beans, and lentils. The unenhanced version of the program delivers nutritional supplementation in the amount of $7. That means that the additional cost associated with this intervention is $19. The nutritional supplement is delivered for 11 of the 12 months of the year. The nutritional supplement is delivered for 11 of the 12 months of the year, which results in a total cost of $209 per child per year. In terms of educational contents, we developed a cooking book that takes into account the socioeconomic characteristics of households in our sample, brochures on food-handling and classification, and 19 nutrition cards that were discussed with the mother during each home visit. Mothers received a nutrition card relevant to their child's age at these monthly home visits. The topics covered included things like breastfeeding, bottle-feeding, breastmilk extraction and storage, weaning, hygiene, finger foods, menu ideas, mealtimes, and chatting while feeding.

TABLE A.1. Composition of the nutritional supplement.

	Calories	Protein	Calcium	Iron	Folic Acid
	Nutritional supplement unenhanced FAMI				
Pregnant woman	16.1%	23.1%	33.8%	22.2%	56.6%
Other adult	3.2%	4.1%	0.9%	7.1%	7.8%
Child	7.6%	12.6%	1.5%	11.0%	17.2%
	Additional supplementation of the intervention				
Pregnant woman	19.0%	31.1%	24.8%	11.0%	36.9%
Other adult	8.0%	18.2%	20.1%	22.3%	42.4%
Child	19.1%	56.2%	32.2%	34.7%	70.8%
	Total nutritional supplement				
Pregnant woman	35.1%	54.2%	58.5%	33.2%	93.4%
Other adult	11.2%	22.2%	21.0%	29.4%	50.2%
Child	26.6%	68.8%	33.7%	45.7%	88.0%

Notes: Nutritional intake requirements.

Appendix B: The Cost and Scalability of FAMI in the Colombian Context

B.1. Costs

The intervention we study costs about $322 per year per child plus $11 one-off cost per child for FAMI pre-service training. The cost of the unenhanced FAMI program is about $327 per child per year. The pedagogical enhancement (excluding the nutritional supplement) corresponds to approximately 35% of the cost of the unenhanced version of the program. This is equivalent to 1.5 monthly minimum wages per child per year, or 2.5 monthly minimum wages per year including the nutritional supplement. For comparison, the cost per child per year in center-based childcare in Colombia is approximately $1,100 or 4.4 monthly minimum wages per child per year.[2] In Table B.1 we compare the costs and impacts of other interventions recently implemented in Colombia, with FAMI.

Bernal (2015) studies the impact of vocational training of the women running the family nurseries considered. She reports a sizeable impact at a low cost per child. Bernal et al. (2019) consider the transfer of children from home-based daycare services offered in the provider's own home to large childcare centers and find virtually no impacts at a very large cost.[3] Finally, Andrew et al. (2019) study the impacts of (1)

2. Cost computations are not adjusted to consider the possibility of crowding in different programs since the intervention we evaluate did not affect the probability of leaving a FAMI to join the new program MF (although it did affect the probability of leaving a FAMI to join an alternative ECD service).

3. The cost reported in the table corresponds to the difference in the cost per child/year in a childcare center and the cost in a family nursery.

targeted pedagogical improvements to center-based care in large cities and (2) staffing of these centers with nutritionists and psychologists. The impacts are comparable to ours at a slightly higher cost for the pedagogical component. Incidentally, the hiring of professional personnel in centers had no effects on children's cognition.

This summary highlights the importance of enhancements to what is known in the specialized literature as process quality (such as the integration of a structured curriculum and improved interactions between caregivers and children supported by coaching and mentoring) with respect to changes and improvements in the so-called structural quality alone (such as infrastructure, as in Bernal et al. (2019); or staffing, as in the second intervention studied in Andrew et al. (2019)). In particular, the former seems to have more cost-effective impacts with respect to the latter. In sum, the evidence we have presented shows that it is possible to gradually improve the quality of nationwide programs at scale in a way that is affordable, while maintaining quality and with a reasonably sized impact on children's developmental outcomes.

TABLE B.1. Costs and impacts of alternative quality enhancements of ECD programs in Colombia,

Program	Age of children at baseline	Cost child/yr (USD)	Impact [a]	Duration of intervention (months)	Detailed result Effect (SE or p-value)
Training para-professional care providers (Bernal 2015)	6 months-5 years of age	101	0.25	12	+5.2 (2.65) score points for children 0-3 years of age on ASQ language scores, and +3.5 (2.0) score points for children 3+ years on Woodcock-Muñoz (WM) mathematical ability.
Transfer from home-based to center-based childcare (Bernal et al. 2019)	6 months-5 years of age	780	0.05	10-18	+0.05 (0.02) SD on nutrition factor, and -0.11 (0.05) SD on ASQ cognitive factor.
Targeted pedagogical improvements in center-based care (Andrew et al. 2019)	18-36 months of age	373	0.15	18	+0.15 (0.076) SD on cognition, language & school readiness (based on TVIP, WM cognition, Daberon and pencil tapping test)
Staffing of center-based care with professionals (Andrew et al. 2019)	18-36 months of age	150	0.10	18	Null effects on cognition. +0.1 (0.06) SD on height for age for children older than 30 months of age.
This study	0-12 months of age	322	0.15	10	+0.16 (p-value 0.044) on Bayley-III factor and -0.06 (p-value 0.54) reduction on the probability of being stunted or in risk of stunting.

Notes: a. SD of standardized scores.

B.2. Scalability

When thinking about the scalability of the FAMI improvement, we consider the possibility of using the already existing supervision infrastructure at ICBF. The national ICBF office works with 33 regional offices and 203 local (municipality-level) offices. Each local office has three different teams in charge of the main tasks. One of the teams, called the community team, is composed of 1 nutritionist, 2 pedagogues, 1 sociologist, 1 social worker and 1 educator. This team oversees supervision and

monitoring of ECD services. The other two teams are responsible for child protection services and intra-household legal issues.

Supervision and monitoring of ECD providers is through regular on-site visits structured around a checklist. This checklist is based upon items that we would consider structural quality features, such as, the physical characteristics of the center, the cleanliness of bathrooms, furniture, etc. It does not cover process quality aspects related to the quantity and nature of the interactions in the classroom (Bernal 2015). A bad evaluation may result in the closure of a center or the need to improve specific aspects to be re-checked within a certain period.

Based on our findings, we consider it would be extremely useful and effective to replace this type of supervision for one that focuses on process quality and uses a reflective approach that encourages best practices. As shown, this could be done by promoting the use of a structured curriculum and session guidelines that identify key features—both in content and form of delivery—that are relevant for child outcomes. These items could be assessed by supervisors, who could provide constructive feedback rather than penalties on the basis of their observations. In this way, continuous improvement would be supported.

There are close to 14,600 FAMI units countrywide. We think it would be possible to train 3 out of the 6 community team members (1 sociologists or 1 educator plus 2 pedagogues) in each local office. The quality and background of these team members is similar to those of the tutors we used in the intervention we evaluated. Each of these supervisors would be in charge of 24 FAMI mothers—a similar caseload as the one we implemented and tested. Similarly, one person in each of the 33 regional offices could be trained by the national office to become trainer of trainers/supervisors.

In our pilot, one senior member of the research team trained a young psychologist in the curriculum, our fieldwork manager). Both trained the 9 tutors during three weeks full-time. After that, the senior researcher was sporadically engaged, but mostly to supervise and provide feedback to the field manager. Each tutor trained approximately 20 to 23 FAMI mothers in sessions that were also 3 weeks long, approximately.

We think it would be feasible to implement a similar cascaded training and supervision process in a potential scale up. Remote online training could be used as a more efficient way of providing pre- and in-service training at the first level of the cascade. Practices by pairs could be supervised online and practices with children could be videotaped for the trainer to provide feedback on the recordings. If required, part of the training could be in-person.

Members of the research team have experience with similar at-scale training strategies for other purposes. In particular, in 2017 we piloted a training scheme that combined presential and online activities for staff in local offices to routinely collect data on child development (the TVIP test) nationwide. Even if connectivity was particularly problematic at the time, this constraint might be easing as digital services expand in response to the COVID- 19 pandemic.

Appendix C: Study Design: Power Calculations and Final Sample

Power calculations assumed program effects of 0.25 of a SD relative to the control on the Bayley-III. These were obtained using an average of 4 FAMI units per town and 4 children aged 0-12 months of age per FAMI. We assumed an intra-class correlation within towns of 0.04 (in the Bayley-III scale and conditional on observables), based on the data in Attanasio et al. (2014) for a similar study in Colombia. This sample design provided 95% power at the 5% significance level, allowing for an attrition rate of 10%.

Towns in the final sample had an average of four FAMI units (SD of 2.3, range between 1 and 13), which translates in a total of 171 FAMI units that received treatment and 169 FAMI units that remained as control.

Figure C.1 presents the c-RCT flow chart, which shows how the final study towns were selected (Figure C.1). Final C.2 depicts the final geographic location of the sample.

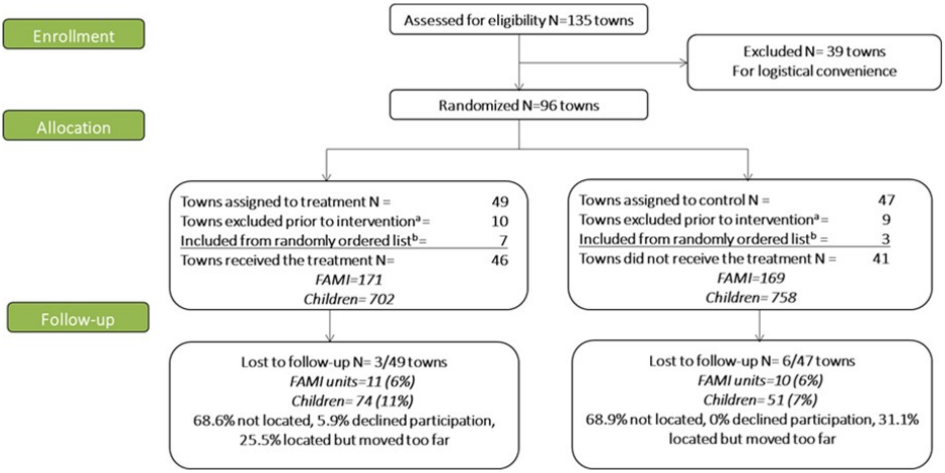

FIGURE C.1. Study's flow chart. [a] Once in the field for data collection, we realized some towns did not have any FAMI units as they had made the transition to other public parenting programs (Modalidad Familiar or MF). [b] Towns in the list of 39 towns excluded initially from the sample, were randomly ranked and used as replacements. However, we did not have enough replacement towns in all randomization strata.

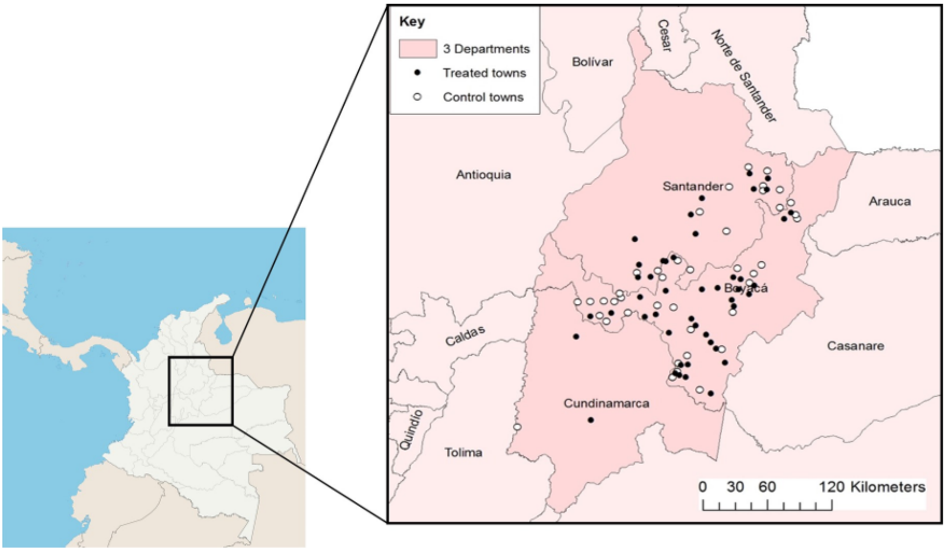

FIGURE C.2. Geographic location of the sample. Treated towns depicted in black and control towns depicted in white.

Appendix D: Construction of Bayley III-factor and Parental Investment

As mentioned in Section 3.1, we used Bayley-III age-standardized cognitive, receptive and expressive language, and fine and gross motor to measure child's development. For parental investment, we used the number of magazines, books, or newspapers in the home, the number of toy sources, the number of varieties of play materials in the home, and the number of varieties of play activities the child engaged in with an adult over the three days before the interview. As these are noisy measures of child development and parental investment we follow Cunha and Heckman (2008) and Heckman et al. (2013) and implement a dedicated measurement system that links each observed measure to one latent factor.

We define M_k as the number of measures of the k-th latent factor (i.e., child development or parental investment), and m_{jk} as the j-th measure of the k-th latent factor. Assuming each measure is additively separable in the logarithm of the latent factor we specify:

$$m_{jk} - \mu_{jk} = \alpha_{jk} \ln \theta_k + \varepsilon_{jk} \tag{D.1}$$

where the terms μ_{jk} are the intercept scaled to zero, α_{jk} are the loadings, θ_k the k-th latent factor, and ε_{jk} are the mean zero measurement error terms assumed to be independent of the latent factors and from each other. This specification assumes that the measurement system is invariant across treatment status.

As the latent factors are unobserved, they have no natural scale and identification requires normalizations (Anderson and Rubin 1956). Then, we set the scale for parental investment by setting $\text{Var}(\ln \theta_{PI}) = 1$, while for the child's development factor by

setting the loading of the Bayley-III cognitive scale to one, that is $\alpha_{1CD} = 1$. Finally, we set the location by setting $E[\ln \theta_k] = 0$ for $k = \{CD, PI\}$, where CD and PI, refer to child development and parental investment, respectively.

In Table D.1 we look at the fraction of the variance in each measure that is explained by the variance in signal. All measures are far from having 100% of their variance accounted for by signal, which illustrates the usefulness of the latent factor approach.

TABLE D.1. Signal-to-noise ratios.

Measurement	Signal
**Bayley-III factor**	
Bayley: Cognitive	0.498
Bayley: Receptive language	0.627
Bayley: Expressive language	0.464
Bayley: Fine motor	0.416
Bayley: Gross motor	0.327
**Parental Investment**	
FCI: Number of toy sources	0.161
FCI: Number of types of play materials	0.697
FCI: Number of types of play activities in last 3 days	0.704
FCI: Number of books, magazines, or newspapers	0.332

Notes: This table shows the fraction of the variance in each measure that is explained by the variance in signal.

Appendix E: Descriptive Statistics for FAMI Facilitators

TABLE E.1. Baseline characteristics of FAMI program facilitators by randomization status.

	Treatment		Control			
Variables	Mean	SD	Mean	SD	p-value	RW
Age	41.80	(10.04)	41.41	(10.37)	0.790	0.992
Education (years)	13.26	(1.66)	13.00	(1.97)	0.378	0.986
Work experience (years) (n1=171, n0=167)	11.71	(7.97)	11.88	(8.52)	0.856	0.992
Number of children	2.71	(1.36)	2.55	(1.50)	0.307	0.968
MC's household size	3.94	(1.48)	3.95	(1.43)	0.949	0.992
Number of children (0-12 months) attending	4.87	(2.06)	5.12	(2.29)	0.504	0.992
Number of pregnant women attending	1.86	(1.34)	1.96	(1.45)	0.588	0.992
Number of group sessions held last month (n1=169,n0=167)	5.46	(4.50)	5.09	(3.40)	0.608	0.992
Number of home visits held last month (n1=170, n0=166)	12.05	(6.66)	13.51	(7.13)	0.209	0.911
Hours devoted to planning activities (n1=168, n0=169)	4.95	(3.02)	6.86	(6.93)	0.013**	0.210
Peabody Picture Vocabulary Test (Z-score)	0.16	(1.03)	-0.17	(0.95)	0.062*	0.545
Knowledge about ECD (Raw Score: correct)	7.29	(1.72)	7.11	(1.40)	0.383	0.986
Single, divorced, or widowed (%)	24	-	21	-	0.591	0.992
No. of observations	171		169			

Notes: * $p < 0.10$, ** $p < 0.05$, *** $p < 0.01$. Standard deviations clustered by town in parenthesis. n1 and n0 are the number of observations on the treatment and control group, respectively. Adjusted p-values using the Romano-Wolf (Romano and Wolf 2005, 2016) procedure (2,000 iterations, clustered by town) are included in the last column. All variables in the table are considered as one group of hypotheses.

Appendix F: Attrition, Compliance, and Dosage Analysis

TABLE F.1. Attrition analysis.

Explanatory variables	Dependent variable: Lost at FU		
ITT	0.0383*	0.0401*	-0.0283
	(0.0213)	(0.0208)	(0.0462)
Age at BL (in months)		0.0159*	0.0139
		(0.0088)	(0.0092)
Age squared		-0.001	-0.0012
		(0.0008)	(0.0008)
Child Gender		-0.0176	
		(0.0127)	
First Born		0.0394**	0.0277
		(0.0165)	(0.0253)
High Household Wealth		-0.0279*	-0.0028
		(0.0148)	(0.0191)
Maternal Years of Education		-0.0011	
		(0.0026)	
Father is Present		-0.0450**	-0.0585**
		(0.0208)	(0.0269)
Household Size		-0.0094	
		(0.0066)	
Maternal PPVT		-0.0018	
		(0.0011)	
ITT * Age			0.0090**
			(0.0039)
ITT * First Born			0.04
			(0.0337)
ITT * High Household Wealth			-0.0524*
			(0.0284)
ITT * Father is Present			0.029
			(0.0411)
Constant	0.0675***	0.1408***	0.0718**
	(0.0116)	(0.0469)	(0.0344)
Observations	1,456	1,456	1,456
R-squared	0.0047	0.0334	0.0334
F-stat	3.229	2.908	3.281
Prob $>F$	0.076	0.004	0.001

Notes: * $p < 0.10$, ** $p < 0.05$, *** $p < 0.01$. Standard errors clustered by town in parenthesis.

Attrition. In Table F.1, we analyze attrition between baseline and follow up. The table indicates that children in treated FAMIs were more likely to be lost at follow up, although the size of this impact is only marginally significant, both with and without controls for a number of baseline characteristics. In the Table we report estimates obtained with a Linear Probability Model.

Compliance. In Table F.2 we look at the possibility that the intervention affected the probability of leaving FAMI for alternative programs. We find a significant reduction of leaving FAMI. This result is robust across different specifications (with and without controls) and to different statistical models. In the table we report estimates obtained with a Linear Probability Model, a Probit, and a Logit model.

TABLE F.2. Compliance analysis: impact of the program on the probability of leaving FAMI for an alternative ECD program.

Explanatory Variables	Dependent variable: Change from FAMI to other ECD program between BL and FU					
ITT	-0.100*	-0.123**	-0.100*	-0.119**	-0.100*	-0.123**
	(0.056)	(0.051)	(0.056)	(0.051)	(0.056)	(0.051)
Child Gender		0.008		0.010		0.010
		(0.028)		(0.027)		(0.027)
Attendance to ECD at BL		0.027		0.031		0.035
		(0.056)		(0.058)		(0.059)
Town's population range		-0.105*		-0.104*		-0.106*
		(0.055)		(0.054)		(0.055)
High Household Wealth		0.068**		0.069**		0.071**
		(0.034)		(0.033)		(0.033)
Teenage mother		-0.016		-0.014		-0.017
		(0.034)		(0.032)		(0.033)
Maternal PPVT		0.012***		0.012***		0.011***
		(0.002)		(0.002)		(0.002)
Department FE: 1		-0.052		-0.056		-0.044
		(0.225)		(0.224)		(0.213)
Department FE: 2		0.032		0.032		0.026
		(0.060)		(0.063)		(0.064)
Observations	989	989	989	989	989	989
Model	LPM	LPM	Probit	Probit	Logit	Logit

Notes: * $p < 0.10$, ** $p < 0.05$, *** $p < 0.01$. Standard errors clustered by town in parenthesis. Average marginal effects presented.

In Table F.3 we look at the effect of the intervention on the probability of leaving FAMI to join Modalidad Familiar. We do not find any evidence of a significant effect.

TABLE F.3. Compliance analysis: impact of the program on the probability of leaving FAMI for an alternative ECD program.

Explanatory variables	Dependent variable: Change from FAMI to MF between BL and FU					
ITT	-0.029	-0.051	-0.029	-0.053	-0.029	-0.054
	(0.048)	(0.046)	(0.048)	(0.047)	(0.048)	(0.048)
Child Gender		-0.023		-0.020		-0.021
		(0.021)		(0.021)		(0.021)
Attendance to ECD at BL		0.015		0.025		0.023
		(0.042)		(0.047)		(0.050)
Town's population range		-0.039		-0.044		-0.041
		(0.049)		(0.049)		(0.053)
High Household Wealth		0.020		0.023		0.022
		(0.025)		(0.025)		(0.025)
Teenage mother		-0.032		-0.032		-0.034
		(0.025)		(0.026)		(0.026)
Maternal PPVT		0.008***		0.007***		0.007***
		(0.003)		(0.002)		(0.002)
Department FE: 1		-0.126***				
		(0.035)				
Department FE: 2		0.006		-0.013		-0.014
		(0.040)		(0.045)		(0.045)
Observations	770	770	770	770	770	770
Model	LPM	LPM	Probit	Probit	Logit	Logit

Notes: * $p < 0.10$, ** $p < 0.05$, *** $p < 0.01$. Standard errors clustered by town in parenthesis. Average marginal effects presented.

Dosage. The following set of Figures show the distribution of children in the intervention group by the duration of exposure to the pedagogical component of the program; as well as joint compliance between the nutritional and pedagogical components. Table F.4 compares household characteristics by total exposure to the program.

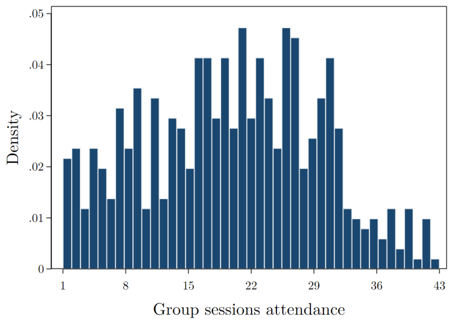

(a) Group sessions

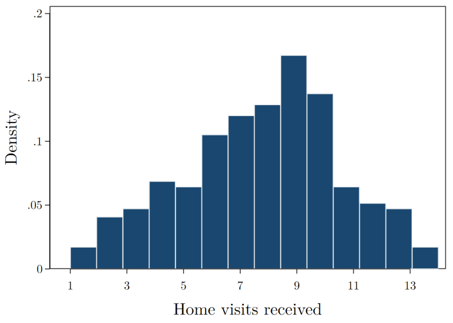

(b) Home visits

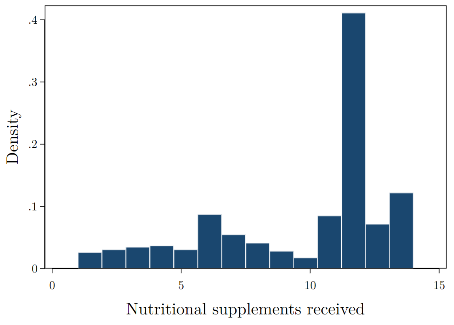

(c) Nutritional supplement

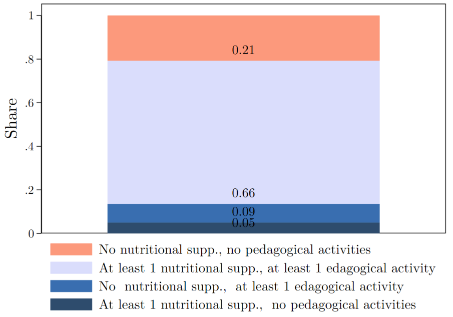

(d) Joint compliance with the nutritional and pedagogical components

FIGURE F.1. Effective individual program participation. In Panels (a) and (b) program attendance registry was recorded by FAMI facilitators. In Panel (a) plots the density over the subsample of children registered at least once in group session attendance lists (74.2% of all treated children assessed at baseline). In panel (b) plots the density over the Subsample of children registered at least once in home visit attendance lists (71.7% of all treated children assessed at baseline). Panel (c) information comes from Fundación Éxito. The maximum nutritional supplements during the intervention period was 14. Pedagogical activity refers to group session or home visit.

TABLE F.4. Differences in child and household characteristics by duration of exposure to the program.

	Attendance above median		Attendance below median		
	Mean	SD	Mean	SD	
**Sociodemographic characteristics**					
Child's age in months	5.43	(3.31)	6.07	(3.47)	***
Male	0.53	(0.50)	0.50	(0.50)	
Child's birth weight (gr) (n1=358, n0=326)	3141	(543)	3245	(599)	**
Birth order	1.11	(1.35)	1.00	(1.35)	
First born	0.43	(0.50)	0.50	(0.50)	
Maternal years of schooling	8.58	(3.48)	9.15	(3.33)	*
Father present	0.74	(0.44)	0.65	(0.48)	**
Number of siblings	1.06	(1.25)	1.0	(1.33)	
Mother married / cohabiting	0.71	(0.45)	0.71	(0.45)	
Mother is single	0.22	(0.41)	0.28	(0.45)	
Mother is divorced	0.01	(0.12)	0.01	(0.09)	
Teenage mothers	0.23	(0.42)	0.28	(0.45)	
Mother's age (years)	26.79	(7.00)	25.49	(6.59)	**
Household wealth index (n1=366, n0=334) [a]	0.02	(0.95)	0.10	(0.96)	
No. of observations	366		335		
**Intermediate Outcomes**					
FCI No. of adult books, magazines and newspapers (n1=365, n0=335)	2.54	(3.07)	2.62	(3.10)	
FCI No. of toy sources	1.33	(0.94)	1.38	(0.93)	
FCI No. of varieties of play materials	1.33	(1.37)	1.52	(1.45)	
FCI No. of varieties of play activities over past 3 days	2.45	(1.51)	2.63	(1.60)	
FCI No. of parental care activities over past 3 days	4.65	(1.13)	4.86	(0.92)	***
Parental Investment (n1=365, n0=335) [b]	-0.07	(0.98)	0.05	(0.99)	
Social support DUKE UNC-11 total (raw score) (n1=362, n0=331)	40.75	(8.39)	41.77	(8.11)	
High maternal self-efficacy (above median) (n1=366, n0=334)	0.39	(0.49)	0.41	(0.49)	
Mothers with depression symptoms (n1=363, n0=332)	0.14	(0.35)	0.17	(0.38)	
Use of verbal or physical abuse in the household (%)	0.01	(0.10)	0.04	(0.19)	*
No. of observations	366		335		
**Nutritional Status**					
Weight-for-age z-score (n1=351, n0=310)	0.18	(1.42)	0.34	(1.35)	
Length/height-for-age z-score (n1=345, n0=308)	0.00	(1.66)	-0.03	(1.73)	
BMI-for-age z-score (n1=338, n0=298)	0.33	(1.64)	0.39	(1.62)	
Weight-for-length/height z-score (n1=331, n0=294)	0.41	(1.56)	0.32	(1.62)	
Food-Insecure (n1=366, n0=335)	0.47	(0.50)	0.54	(0.50)	

Notes: * $p < 0.10$, ** $p < 0.05$, *** $p < 0.01$. Median= 21 activities (groups sessions and home visits). n1 and n0 are the number of observations above and below the median, respectively. Standard errors clustered by town in parenthesis. [a] The wealth index was computed as the principal component of a set of dichotomous variables that describe characteristics of the household, ownership of durable goods, and access to public utilities. [b] Factor score of FCI items.

Appendix G: Main Impacts (ITT) Corrected for Attrition

In Table G.1, we report the estimates of the impact we obtain by modeling attrition as a selection process. The identifying assumption of the impact results is that the quality and motivation of the interviewers (randomly assigned to both treatment and control towns) does not affect the outcome of interest while determining the probability of attrition. We find that interviewers dummies are a significant determinant of attrition and that the results on the impacts of the program are virtually unaffected by non-random attrition.

TABLE G.1. Program impacts on children's outcomes estimated by maximum likelihood correcting for self-selection into the follow up sample.

VARIABLE	Impacts (95% CI)	*P* Value	RW *P* Value
Bayley-III Factor	0.175<br>(0.044,0.305)	0.009***	0.032**
ASQ:SE Total Score	-0.075<br>(-0.246,0.095)	0.387	0.579
Height-for-age *Z*-Score	0.075<br>(-0.038,0.187)	0.194	0.393

Notes: * $p < 0.10$, ** $p < 0.05$, *** $p < 0.01$. Confidence interval in parenthesis for two-tailed tests. Standard errors clustered by town. p-values are computed using Romano-Wolf (RW, Romano and Wolf (2005, 2016)) step-down procedure. We consider 3 hypotheses for children's outcomes. Exclusion restrictions: interviewer fixed effects at baseline and assigned interviewer fixed effects at follow-up. First stage F-stat=11.24. Covariates: child's gender, an indicator of high household wealth index, maternal PPVT score, teenage mother, an indicator of high municipality population, previous attendance to a child care center, department fixed effects, and baseline weight-for-age and height-for-age Z-scores. Bayley-III factor is the factor score of the age-standardized Bayley III scales. ASQ:SE Total Score is the age-standardized ASQ:SE score.

Appendix H: Evaluation of an Integrated Intervention Targeted at Deprived Pre-School Children in Rural Colombia Pre-Analysis Plan. 22/06/2016

H.1. Introduction

This document outlines a pre-analysis plan—study design, hypothesis to be tested, and data and specifications to be used—for evaluating the impact of the integration of a structured curriculum (both, home visits and group meetings) to promote young children's development into the FAMI (Hogares Comunitarios - Modalidad Familia, Mujer e Infancia) parenting programme for disadvantaged families in rural Colombia. The program is being implemented by the research team in cooperation with the National Family Welfare Agency (Instituto Colombiano de Bienestar Familiar - ICBF). The intervention and evaluation have been funded by Grand Challenges Canada (GCC) and the Fundación Éxito (FE). Baseline data collection took place between August

and November 2014. Follow-up data will take place between April and July 2016. The intervention ran from September 2014 through March 2016. This plan has been written up prior to follow-up data processing, serving as a pre-commitment for subsequent analysis.

This document is structured as follows. Section 2 reviews the intervention and evaluation design. Section 3 enumerates the hypotheses to be tested as part of the study and the data we will use to test them. Finally, Section 4 outlines the empirical specification(s) to be used in analyzing the data and other data management issues.

H.2. Overview of the Study: Interventions and Evaluation Design

Hogares Comunitarios – Modalidad Familia, Mujer e Infancia (FAMIs): are small-sized community centers located in areas of high social and economic vulnerability in semi-urban and rural areas of Colombia where pregnant women and parents of children younger than two years of age receive training regarding parental practices including family relationships, pregnancy, breastfeeding, nutrition, health and the upbringing of young children. The program targets socioeconomically vulnerable pregnant women, nursing mothers and parents of children less than 2 years of age. Program eligibility is defined by the national proxy means test (PMT) known as SISBEN, which classifies households into socioeconomic vulnerability levels based on a household survey. A front line worker known as Community Mother (MC) works 80 hours per month for the program. In particular, she devotes 32 hours for parental training in group sessions; 20 hours of home visits (minimum 1 visit per family per month); 8 hours of training for the MC; and 20 hours for planning activities, documentation and transportation times. Group sessions are held separately by age subgroups according to the child's age: children from 0 to 5 months old, children from 6 to 11 months old, 1 to 2 years old children and pregnant women. Each FAMI unit has an average of 12 to 15 beneficiaries.

The FAMI program has two main components: i) the provision of nutritional supplement – that should cover 20 to 25% of daily nutritional requirements of the child or the pregnant women; and ii) training of beneficiary families on parental practices and child development since gestation and up to age two, particularly regarding nutrition, socioemotional development, health, maternal health, and early stimulation through group sessions and one-on-one home visits.

Our study offers a rigorous evaluation, by Randomized Controlled Trial (RCT), of the short-term impacts of *the integration of a structured curriculum* (*both, home visits and group meetings*) *to the FAMI program and the addition of rigorous training and supervision protocols for front line workers.*

H.2.1. The Intervention. Based on a rigorous study on the weaknesses of the program and a small pilot on the improvements that were to be developed, the team designed an integrated upgrade of the FAMI program. The upgrade consists of 3 components. First, the implementation of a structured but feasible, flexible and effective curriculum (both for home visits and group meetings) focused on encouraging mother-child interactions and maternal self-efficacy, teaching mothers how to promote

their children's development and promoting maternal self-esteem and mental health. This component is complemented by the provision of pedagogical materials such as puzzles and books, materials for home-made toys and toy making workshops during group sessions. Second, the improvement of MCs' training, provided by professional tutors trained by the research team, in order to guarantee the fidelity of program implementation and the addition of a supervision and coaching protocol for front line workers to be delivered throughout the duration of the intervention. Third, the delivery of an additional nutritional supplement (increased intake of calorie, protein, vitamins and minerals), complemented with psychoeducation around feeding and nutrition during sessions, and informative materials to promote healthy nutritional habits.

H.2.2. Sample and Evaluation Design. The evaluation sample consists of 1,466 children 0-12 months at baseline and 553 pregnant women in 340 FAMIs located in 87 municipalities of three Colombian departments of the Andean region: Boyacá, Santander and Cundinamarca. These municipalities were selected for their geographical location, their semi-rurality and rurality conditions and the presence (or not) of other similar ECD services such as another parenting program similar to the one included in this study, known as MF or *modalidad familiar*.

From a universe of 151 eligible municipalities (i.e., municipalities with less than 40.000 inhabitants, at most one MF and that belong to Boyacá, Santander, Cundinamarca and Tolima departments), we selected all municipalities with no MFs and then we selected from the remaining municipalities with at most one MF, striving to achieve distributed geographic coverage, until we reached 96 town. Then we ran a stratified randomization based on (i) MF (*modalidad familiar*) presence, (ii) Department and (iii) Population size (less than or more than 10.000 inhabitants) resulting in 49 municipalities in the treatment group and 44 in the control group. We then dropped FAMIs that were transiting or were going to transit to the new version of the program (MF) and, as a consequence, had to drop all study municipalities located in the department of Tolima since no control municipalities were left for this group. This procedure implies a final sample of 41 municipalities with 169 FAMIs in the control group and 46 municipalities with 171 FAMIs in the treatment group.

Baseline data collection on the children and the pregnant women, their households, the MCs and the centers they attend took place between August and November 2014. Follow-up data collection will take place from April to July 2016. We collected baseline data directly in participants' homes, except in those instances in which it was not possible to interview the mother of the child in her own home, in which case the interview took place in community centers at the town's urban center such as schools, churches or in the FAMI.

The analysis of baseline data shows that the sample is balanced across the evaluation groups. Whilst there are some significant differences in children's nutritional status and a few socio-demographic characteristics (e.g. presence of the father and mother's TVIP and personality), and in some of sociodemographic characteristics of MCs, none of these differences systematically occur in one of the study groups nor point towards a specific (bias) direction.

H.3. Hypotheses to be Tested and Data

We have collected (at baseline) and are collecting (at follow-up) a rich set of data and we will use it to test a series of hypotheses concerning the impacts of the intervention under study. We present the study hypotheses in two groups: impact on children's outcomes and impact on mothers' parenting abilities and on the learning environment at home.

H.3.1. Hypotheses Group A: Impact on Children's Outcomes. The treatment may have positive average impacts on outcomes for children attending the program. We group these outcomes into two areas: children's development and children's nutritional status.

We consider a number of domains within development—namely, some aspects of cognition, language and motor and socio-emotional development. We next list the specific hypothesis on each of them by domain of development, and detail the specific tests (and scales) we will use to measure them and how we will process the data. We would however like to flag two considerations before proceeding.

On the one hand, we would like to clarify that we plan to use factor analysis (on standardized scores) to determine the most appropriate way of combining the various tests and scales collected in "constructs". The reason for this is that child development is composed of many different dimensions that are interrelated. For example, even if a vocabulary test should be viewed as an achievement test as opposed to a measure of raw ability (given that it measures acquired vocabulary) it nevertheless correlates well with ability. Hence, it is very difficult to establish a priori the most sensible way to organize the data, and we plan to rely on factor analysis to combine data that captures common underlying constructs (data that would be thought to go together on theoretical grounds). To do this, we will follow standard protocols in the use of factor analysis. First, we will construct as many factors ("constructs") as there are with eigenvalues larger than 1. Next, we will only use outcomes (scales or tests) with factor loads larger than 0.4 in the construction of these factors. Hence, the following categorization of scales and tests in domains is for the purpose of illustrating our hypothesis and may be modified as a result of the outcomes of factor analysis.

In addition to the main analysis based on the impacts on the factors we have identified, we will also report impacts on the individual tests, correcting our p-values for multiple hypotheses testing, using the Romano-Wolf step-down procedure (Romano and Wolf 2005), as further discussed in Section 5.

On the other hand, note that the following child development outcomes, listed under Hypotheses Group A, will be collected at follow-up only (as they were not suitable for administration to children at baseline given their age) by direct administration to children by a trained psychologist at a community venue in each town. An expert psychologist with extensive training and practice in the collection of the assessment instruments included in this study trained a group of 10 psychologists who administered the assessments in the field. The only exception to this is the ASQ:SE, which is collected by maternal report (direct interview with the mother, and

was included as part of the household questionnaire). Children were 0-12 months of age at baseline and will be 15-29 months of age at follow up, depending on the exact time at which they are assessed at follow-up. At baseline we collected different child outcomes, which will be used as baseline controls, as explained in Section 4.

HYPOTHESIS H.1. *The treatment is likely to have a positive average impact on children's cognitive development, language development, and motor development.*

These domains will be assessed using the indicators listed next.

- Cognitive Development: some dimensions of cognition will be assessed by the Spanish Version of the Cognitive Scale of the Bayley Scales of Infant and Toddler Development, Third Edition (Bayley-III) (Bayley 2006). The scale evaluates the sensor and motor development, exploration and manipulation, the relationship of objects, concept formation, memory, and other aspects of cognitive processing of the child.
  - The scales will be administered and scored as indicated in the Bayley-III administration manual. Higher scores indicate higher cognitive abilities. However, given the process of development, scores are also likely to increase with age.
- Language Development: we will assess both receptive and expressive language.
  - Receptive language will be assessed using the Spanish version of the Receptive Communication Scale of the Bayley Scales of Infant and Toddler Development, Third Edition (Bayley-III) (Bayley 2006). The scale evaluates pre-verbal behaviors and vocabulary development – i.e. the ability to identify objects and images that are been referenced.
    - The scale will be administered and scored as indicated in the Bayley-III administration manual. Higher scores indicate higher receptive language development. However, given the process of development, scores are also likely to increase with age.
  - Expressive language will be assessed using the Spanish version of the Expressive Communication Scale of the Bayley Scales of Infant and Toddler Development, Third Edition (Bayley-III) (Bayley 2006). The scale evaluates pre-verbal communication, such as babbling, gesticulation, joint referencing and early talking; and vocabulary development, such as naming objects, images and attributes (eg, color and size).
    - The scale will be administered and scored as indicated in the Bayley-III administration manual. Higher scores indicate higher expressive language development. However, given the process of development, scores are also likely to increase with age.
- Motor Development: we will assess both fine and gross motor development.
  - Fine Motor Development will be assessed using the Spanish version of the Fine Motor subset Scale of the Bayley Scales of Infant and Toddler Development, Third Edition (Bayley-III) (Bayley 2006). The scale evaluates the child's skills related to visual tracking, reaching, gripping and manipulation of objects.

Functional abilities of the child's hand and responses to tactile information are also measured.
  – o The scale will be administered and scored as indicated in the Bayley-III administration manual. Higher scores indicate higher fine motor development. However, given the process of development, scores are also likely to increase with age.
- Gross Motor Development will be assessed using the Spanish version of the Gross Motor subset Scale of the Bayley Scales of Infant and Toddler Development, Third Edition (Bayley-III) (Bayley 2006). The scale evaluates the child's static position (e.g. when the child is sitting or standing); dynamic movement, including locomotion and coordination; balance; and planning of movements.
  - The scale will be administered and scored as indicated in the Bayley-III administration manual. Higher scores indicate higher gross motor development. However, given the process of development, scores are also likely to increase with age.

HYPOTHESIS H.2. *The treatment is likely to have a positive average impact on children's socio-emotional development.*

- Socio-emotional development: we will assess socio-emotional development using the Ages and Stages: Socio-Emotional Questionnaire (ASQ:SE), which screens several socio-emotional areas such as self-regulation, compliance, communication, adaptive behaviors, autonomy, affect, and interaction with people, for children 15-29 months at follow-up by parental report.
- The scale will be administered and scored as indicated in the ASQ:SE manual.
- While scores should not be age dependent, we will remove any lingering age effect standardizing the scores internally, i.e. using the distribution empirical mean and standard deviation, estimated using non-parametric regression methods.

HYPOTHESIS H.3. *The treatment is likely to have a positive average impact on children's nutritional status.*

We expect the intervention to improve nutritional status through the addition of a nutritional supplement, the education provided about feeding and nutrition during sessions and home visits, and the provision of informative materials to promote healthy nutritional habits. Children's nutritional status is measured both at baseline and at follow-up by personnel that is trained by an expert nutritionist and assessed for reliability. In particular, we collected information on height, weight and body mass index (BMI) following World Health Organization (WHO) standards (World Health Organization 2006, 2007) for all children in our sample, both at baseline and follow-up. Based on these measures, we will construct a variety of nutritional indicators

depending on the child's age and based on World Health Organization (2006, 2007) standards.

Hypothesis H.4. *The treatment is likely to have a positive average impact on children's food insecurity status.*

We expect the intervention to improve the household's and the child's food insecurity status as a result of the nutritional supplement delivered with the program. We assess food insecurity status using the Latin-American and Caribbean Nutritional and Food Insecurity Scale (ELCSA) adapted and validated for this population.

H.3.2. Hypotheses Group B: impact on the mother's parenting skills and the learning environment at home. The intervention is more likely to improve children's outcomes if the children's mothers effectively internalized the message and training delivered by the program's new curriculum and took these lessons to practice with their own children. In particular, the program might have been more effective if maternal self-esteem, mental health and motivation improve as a result of program participation; if the quality of the home environment improves; if discipline practices at home are more appropriate; and if parents devote more time to stimulating activities with their children such as reading and playing. Also, these things are more likely to have happened if parents participated more regularly in the program's group sessions and home visits.

Common Hypothesis B. *We will test the hypotheses that the intervention will have an impact on mother's parenting skills, parental knowledge and perceptions, parental self-efficacy, mental health, and the home environment. We also hypothesize that these changes in parental practices, knowledge, perceptions, self-esteem, mental health, motivation and changes in the learning environment at home will correlate with (and contribute to—i.e. mediate) the impacts on children's outcomes described above (Hypotheses Group A).*

We next describe the outcomes previously listed and how we will construct them.

1. *Maternal self-efficacy:* To measure maternal efficacy, we use an adapted version of the 15-item Self-Efficacy in the Nurturing Role Questionnaire (Pedersen et al. 1989; Gibaud-Wallston and Wandersman 1979). Mothers responded Yes, No or Unsure to indicate how they felt about their role as a parent.
2. *The quality of the home environment is improved:* We will use an adaptation of a subset of questions from the UNICEF scale of the home environment quality: The Family Care Indicator (FCI) - Version 16 (Frongillo et al. 2003) to measure the quality of the home environment. The FCI measures the availability and variety of play materials, sources of these materials and books and magazines in the child's household.
3. *Discipline strategies used by parents:* We will use an adaptation of the domain III of the UNICEF Care Indicator Questions (version 16). The questionnaire inquires about the discipline strategies most frequently used by parents at home with their

children. In particular, the questions aim at determining how frequent non-violent strategies, psychological aggression strategies, and physical punishment strategies are used with children.

4. *Parental time devoted to stimulating activities with their children:* We will use an adaptation of a subset of questions of the Family Care Indicator (FCI) - Version 16 (Frongillo et al. 2003) which measures the amount of time devoted by adults in the household to a variety of activities, including personal care routines, with the child during the three days prior to the interview date.
5. *Parental knowledge about early childhood development:* We use a 10-item scale to assess maternal factual knowledge of childcare/parenting practices, child developmental processes, and milestones. This scale has been piloted by the research team but has not been formally assessed for reliability and validity.
6. *Parental mental health:* We use the CESD-10 scale to assess maternal mental health. The 10-item Center for the Epidemiological Studies of Depression Short Form (CES-D-10) is a widely used measure to screen for depression. The 10-item measure has demonstrated strong psychometric properties, including predictive accuracy and high correlations with the original 20-item version. It is generally used to assess depression symptom severity rather than as a diagnostic screening tool (Andresen et al. 1994).
7. *Parental social support:* We use the DUKE-UNC Functional Social Support Questionnaire 11 to assess the mother's perception about the amount and type of functional social support that she receives. Given the characteristics of the intervention, we seek to understand whether parenting group meetings contribute to a perception of enhanced social support, which might contribute towards maternal self-efficacy and maternal mental health.
8. *Mothers attend FAMI group sessions and receive home visits:* We will use self-reported data on attendance and turnover, collected from the main caregiver, and from administrative data on attendance.

H.4. Use of the Qualitative and Process Data

In addition, as part of a qualitative evaluation, we are collecting process data on a subsample of FAMIs that will contribute to inform the quality (and fidelity) of the program, and provide insights on the extent to which the mechanisms through which the program has (or did not have) impacts occurred. The objective of this qualitative component is to characterize the interventions in the field with a detailed tracking of activities and responsible individuals related to the components of the interventions under study. Based on direct observations, in-depth interviews and session videotaping we hope to gain a better understanding of the ways in which the interventions are understood, adopted and used directly by MCs. In addition, interviews to beneficiary mothers are taking place, to capture mother's perceptions about the program's strengths and weakness.

With this input we hope to be able to (1) better interpret our quantitative estimates of program impacts, (2) identify possible transmission mechanisms of the effects of

interest, and (3) propose specific policy recommendations for program improvement based on the joint analysis of the quantitative and the qualitative results.

H.5. Empirical Strategy

H.5.1. Baseline Specification. Given the experimental design described in Section 2, we can identify the impacts of the treatment on outcomes using the following estimating equation:

$$Y_{isl,1} = \beta_0 + \beta_1 T_{sl} + \gamma Y_{isl,o} + X'_{isl,0}\delta + D_{isl,0}\theta + F_{isl,0}\sigma + S_{isl,0}\tau + Z_{isl,1}\rho + \varepsilon_{isl,1} \tag{H.1}$$

where $Y_{isl,1}$ is the outcome of interest for child i in FAMI center s in municipality l at follow-up (t=1); T_{1sl} is a dummy equal to 1 if the FAMI center s in municipality l receives the treatment; and $Y_{isl,0}$ is the baseline (t=0) level of the outcome of interest (or level of the corresponding aggregate construct in the case that the same measure was not administered at baseline and follow-up) for child i in FAMI center s in municipality l at follow-up. For child developmental outcomes we will not have the same outcome at baseline and follow-up since the tests could not be administered given children's ages at baseline. For these outcomes, we will control for all existing aggregate scores (constructed using factor analysis, as described above) and including all developmental scores and nutritional scores. The purpose of this approach is to maximize efficiency. $X'_{isl,0}$ is a set of basic child and household characteristics, which are also added to improve efficiency (minimize residual variance) and control for the slight imbalance in some baseline characteristics observed between groups at baseline (detailed list pending); $D_{isl,0}$ are a set of department fixed effects, $F_{isl,0}$ are a set of dummies indicating the presence or not of the alternative parenting program in town l (modalidad familiar) and $S_{isl,0}$ are a set of municipality population size dummy variables indicating above and below 10 thousand inhabitants (all included due to our stratified randomization procedure), and$Z_{isl,1}$ are a complete set of tester or interviewer dummies. $\varepsilon_{isl,1}$ is the random error term, clustered at the municipal level l (the unit of randomization).

We can estimate equation (H.1) by OLS. β_1 is the estimated average impact of the treatment on outcome $Y_{isl,1}$; (intent-to-treat estimate). If compliance is not complete in the sense that children do not attend all program sessions they are intended to (and assuming this non-compliance is not larger than 40%) we would additionally estimate duration of exposure to treatment effects by instrumenting actual duration of participation in the program with the result of the random assignment (intention-to-treat or randomized treatment variable). We would also assess the extent to which actual duration of exposure to treatment is correlated with treatment status.

H.5.2. Impact on Mother's Parenting Skills and the Home Environment (Hypotheses Set B). We can also use equation (H.1) to estimate the impact of the treatment on intermediate outcomes. When the impact on mother's parenting skills and the home

environment refer to the child's mother (or other caregiver) we will replace the set of basic covariates in X with mother (or other caregiver) basic baseline or time invariant characteristics (e.g., age and educational attainment).

H.5.3. Dealing with Testing for Multiple Outcomes through Standardized Treatment Effects and Adjustments for Multiple Inference. For some of the developmental domains analyzed in this study, we have more than one outcome measure with which to explore treatment effects. To deal with multiple hypothesis testing we will employ two approaches.

The first approach will be to group our outcome measures into domains or "constructs" using factor analysis (following the procedure described in Section 3) and estimate equation (1) using the resulting factor index as the relevant dependent variable. This procedure is based on the idea that items within a domain are measuring an underlying common "construct" (or factor).

The second approach will consist of estimating each outcome (individual test) independently but adjusting p-values for multiple hypotheses testing using the step-down procedure developed by Romano-Wolf on each set of (Romano and Wolf 2005).

H.5.4. Survey Attrition. We acknowledge that a certain level of attrition is unavoidable. We will check that the sample of non-attriters remains balanced on baseline observables (as is the entire sample). We will also check that attrition is independent of treatment status. In the event of a significant correlation between attrition and treatment status we will estimate the determinants of attrition using a probit regression on observables and if feasible use a Heckman selection correction procedure to adjust the estimates of the main equation (H.1).

H.5.5. Procedures for Addressing Missing Data. We will not impute the values for any dependent variable (final or intermediate outcomes) at follow-up. Regarding missing data on covariates, $Y_{isl,o}$ and $X'_{isl,o}$, we will check whether item non-response is correlated with treatment status. If it is not correlated, we will impute the missing covariate value with the average of the non-missing observations and this imputation will be accounted for with a dummy variable (we will check the robustness of our results by also estimating the regression without that covariate). If non-response in the baseline covariate is correlated with treatment status, we will not use that covariate when estimating the regressions. In cases in which the percentage of observations with covariate missing data is less than 2%, we will simply work with the sample with non-missing data.

H.5.6. Questions with Limited Variation. We will not use as dependent or independent variables any indicator variable that has a prevalence rate of below 10% or above 90%, in order to limit noise caused by variables with minimal variation.

In the event that omission decisions result in the exclusion of all constituent variables (or for as many as indicated in the test manual) for an indicator, the indicator will not be calculated.

H.5.7. Treatment of Outliers. We will drop children with developmental outcomes or nutritional status with standardized values lower than 3 standard deviations below the mean (<-3SD) of the relevant standardized distribution, since we consider this to be an indication of potential disability (for developmental outcomes), severe malnutrition (for nutritional status) or significant measurement error.
