# Supplementary material for: Early Stimulation and Nutrition: The Impacts of a Scalable Intervention
Source: J Eur Econ Assoc. 2022 Jan 28;20(4):1395–432. doi: 10.1093/jeea/jvac005 (PMC9372035; doi:10.1093/jeea/jvac005)
Supplement: jvac005_Attanasio_etal_Replication-Data-Code [file jvac005_attanasio_etal_replication-data-code.zip › replication-data-code/output/table-10/Prod_fun.doc]

IV estimation of the production function for cognitive skills
	OLS	OLS	First Stage	IV		
Instruments:			Distance, wages	Distance, wages, ITT		
	(1)	(2)	(3)	(4)		
ITT	0.135**	0.079	0.294***	-0.006		
	(0.065)	(0.065)	(0.068)	(0.110)		
FCI Home Environment		0.185***		0.467*	0.454***	
		(0.036)		(0.249)	(0.171)	
Time to Town's Hall	-0.099***	-0.079***	-0.040	-0.048	-0.049	
	(0.027)	(0.028)	(0.030)	(0.043)	(0.037)	
Time to FAMI			-0.143***			
			(0.035)			
First stage F-statistic:						
						
IV: Time to FAMI			16.859			
						
IV: Time to FAMI and Treatment			19.149			
						
Overidentification p-value					0.956	
						
Observations	1,292	1,292	1,292	1,292	1,292	
						
