# Supplementary material for: Early Stimulation and Nutrition: The Impacts of a Scalable Intervention
Source: J Eur Econ Assoc. 2022 Jan 28;20(4):1395–432. doi: 10.1093/jeea/jvac005 (PMC9372035; doi:10.1093/jeea/jvac005)
Supplement: jvac005_Attanasio_etal_Replication-Data-Code [file jvac005_attanasio_etal_replication-data-code.zip › replication-data-code/output/table-2/intermediates.doc]

VARIABLE	Treatment	Control	Treatment - Control		
Total Observaciones = 1456	700	756	Differencia	p-value	
Parental investment (bl) n1=698, n0=751 	-0.025	0.030	-0.055	0.625	
	(0.962)	(1.020)	(0.113)		
Parental knowledge of child dev. (fu) n1=626, n0=705 	29.257	29.492	-0.235	0.680	
	(3.611)	(3.443)	(0.570)		
Mother's self-efficacy (bl) n1=700, n0=756 	26.503	26.487	0.016	0.974	
	(5.509)	(4.672)	(0.487)		
ELCSA Food insecurity (bl) (%) n1=700, n0=756 	0.504	0.419	0.085	0.219 	
	(0.500)	(0.494)	[1.514]		
*** Significance at 1%, ** Significance at 5%, * Significance at 10%
() Standard errors in brackets
[] Chi2 Statistic, clustered by Fake Municipality ID (bl)
