# Supplementary material for: Early Stimulation and Nutrition: The Impacts of a Scalable Intervention
Source: J Eur Econ Assoc. 2022 Jan 28;20(4):1395–432. doi: 10.1093/jeea/jvac005 (PMC9372035; doi:10.1093/jeea/jvac005)
Supplement: jvac005_Attanasio_etal_Replication-Data-Code [file jvac005_attanasio_etal_replication-data-code.zip › replication-data-code/output/table-2/sociodemographic.doc]

VARIABLE	Treatment	Control	Treatment - Control		
Total Observaciones = 1456	700	756	Differencia	p-value	
Age (months) (bl) n1=700, n0=756 	5.724	5.513	0.211	0.353	
	(3.391)	(3.257)	(0.227)		
Child's birth weight (gr) n1=683, n0=751 	3,189	3,156	34	0.442	
	(572)	(500)	(44)		
Mother's age (years) (bl) n1=700, n0=756 	26.159	26.472	-0.314	0.421	
	(6.835)	(6.703)	(0.389)		
Mother's education (years) (bl) n1=700, n0=756 	8.849	8.412	0.436	0.121	
	(3.422)	(3.313)	(0.281)		
Household income (thousands COP) (bl) n1=686, n0=736 	526.098	477.242	48.856	0.232	
	(388.110)	(340.705)	(40.900)		
Household size (bl) n1=700, n0=756 	4.084	4.097	-0.012	0.932	
	(1.474)	(1.428)	(0.143)		
Mother's PPVT (bl) n1=700, n0=756 	22.323	19.763	2.560	0.037**	
	(8.527)	(8.083)	(1.225)		
Gender: Male (%) n1=700, n0=756 	0.519	0.509	0.009	0.729 	
	(0.500)	(0.500)	[0.120]		
First born (%) n1=700, n0=756 	0.466	0.451	0.015	0.655 	
	(0.499)	(0.498)	[0.199]		
Teenage mother (bl) (%) n1=700, n0=756 	0.254	0.209	0.045	0.059* 	
	(0.436)	(0.407)	[3.565]		
Father present (bl) (%) n1=700, n0=756 	0.697	0.751	-0.054	0.031** 	
	(0.460)	(0.433)	[4.672]		
Owns home (bl) (%) n1=700, n0=756 	0.371	0.396	-0.024	0.623 	
	(0.484)	(0.489)	[0.242]		
Household in poverty (bl) (%) n1=686, n0=736 	0.587	0.640	-0.052	0.298 	
	(0.493)	(0.480)	[1.084]		
*** Significance at 1%, ** Significance at 5%, * Significance at 10%
() Standard errors in brackets
[] Chi2 Statistic, clustered by Fake Municipality ID (bl)
