# Supplementary material for: Early Stimulation and Nutrition: The Impacts of a Scalable Intervention
Source: J Eur Econ Assoc. 2022 Jan 28;20(4):1395–432. doi: 10.1093/jeea/jvac005 (PMC9372035; doi:10.1093/jeea/jvac005)
Supplement: jvac005_Attanasio_etal_Replication-Data-Code [file jvac005_attanasio_etal_replication-data-code.zip › replication-data-code/output/table-3/nutricion.doc]

VARIABLE	Treatment	Control	Treatment - Control		
Total Observaciones = 1456	700	756	Differencia	p-value	
Weight-for-age z-score (bl) n1=660, n0=708 	0.258	0.269	-0.010	0.921	
	(1.387)	(1.422)	(0.104)		
Length/height-for-age z-score (bl) n1=652, n0=705 	-0.006	-0.206	0.200	0.241	
	(1.684)	(1.743)	(0.170)		
Weight-for-length z-score (bl) n1=624, n0=686 	0.369	0.552	-0.182	0.167	
	(1.591)	(1.645)	(0.132)		
Underweight (bl) (%) n1=660, n0=708 	0.064	0.051	0.013	0.465 	
	(0.244)	(0.220)	[0.534]		
Risk of underweight (bl) (%) n1=660, n0=708 	0.091	0.107	-0.016	0.415 	
	(0.288)	(0.310)	[0.663]		
Wasting (bl) (%) n1=624, n0=686 	0.059	0.064	-0.005	0.775 	
	(0.236)	(0.245)	[0.082]		
Risk of wasting (bl) (%) n1=624, n0=686 	0.109	0.082	0.027	0.179 	
	(0.312)	(0.274)	[1.804]		
Stunting (bl) (%) n1=652, n0=705 	0.092	0.139	-0.047	0.081* 	
	(0.289)	(0.346)	[3.042]		
Risk of stunting (bl) (%) n1=652, n0=705 	0.147	0.155	-0.007	0.793 	
	(0.355)	(0.362)	[0.069]		
Overweight (bl) (%) n1=624, n0=686 	0.099	0.092	0.008	0.707 	
	(0.299)	(0.289)	[0.142]		
Obesity (bl) (%) n1=624, n0=686 	0.048	0.073	-0.025	0.174 	
	(0.214)	(0.260)	[1.847]		
*** Significance at 1%, ** Significance at 5%, * Significance at 10%
() Standard errors in brackets
[] Chi2 Statistic, clustered by Fake Municipality ID (bl)
