# Supplementary material for: Early Stimulation and Nutrition: The Impacts of a Scalable Intervention
Source: J Eur Econ Assoc. 2022 Jan 28;20(4):1395–432. doi: 10.1093/jeea/jvac005 (PMC9372035; doi:10.1093/jeea/jvac005)
Supplement: jvac005_Attanasio_etal_Replication-Data-Code [file jvac005_attanasio_etal_replication-data-code.zip › replication-data-code/output/table-4/dev_outcome_control.doc]

	Mean	N	
	(Sd. Dev.)		
Bayley			
			
Cognitive Composite Score	91.984	703	
	(13.072)		
Language Composite Score	91.587	702	
	(12.306)		
Motor Composite Score	93.966	701	
	(12.580)		
ASQ:SE			
			
% of children at socio-emotional risk	0.379	705	
	(0.485)		
