# Supplementary material for: Early Stimulation and Nutrition: The Impacts of a Scalable Intervention
Source: J Eur Econ Assoc. 2022 Jan 28;20(4):1395–432. doi: 10.1093/jeea/jvac005 (PMC9372035; doi:10.1093/jeea/jvac005)
Supplement: jvac005_Attanasio_etal_Replication-Data-Code [file jvac005_attanasio_etal_replication-data-code.zip › replication-data-code/output/table-5/itt.doc]

VARIABLE	Impact (95 CI)	P Value	RW P Value	
Bayley-III Factor	0.163++	0.015	0.047	
	(0.035,0.290)			
ASQ:SE Total Score	0.021	0.722	0.704	
	(-0.096,0.139)			
Height for age Z-Score	0.078	0.190	0.317	
	(-0.038,0.195)			
