# Supplementary material for: Early Stimulation and Nutrition: The Impacts of a Scalable Intervention
Source: J Eur Econ Assoc. 2022 Jan 28;20(4):1395–432. doi: 10.1093/jeea/jvac005 (PMC9372035; doi:10.1093/jeea/jvac005)
Supplement: jvac005_Attanasio_etal_Replication-Data-Code [file jvac005_attanasio_etal_replication-data-code.zip › replication-data-code/output/table-5/TD-Bayley OLS.doc]

VARIABLE   n1=599, n0=693	Beta (95% CI)	P Value	D	
Bayley-III Factor	0.163	0.015	0.163	
	(0.035,0.290)	**		
Standard Errors Clustered by Fake Municipality ID (bl).
D=(ß/SD controls), where SD controls is standard deviation for control group within estimation sample.
Covariates Included: , Gender: Male, Fake Department ID, Previous attendance to a child care center (bl), Municipality's population category (bl), Household wealth index above the median (bl), Teenage mother (bl), Mother's PPVT (bl), Length/height-for-age z-score (missings replaced) (bl), Replaced missing of Length/height-for-age z-score (bl), Weight-for-age z-score (missings replaced) (bl), Replaced missing of Weight-for-age z-score (bl), Interviewer Bayley (fu)
