# Supplementary material for: Early Stimulation and Nutrition: The Impacts of a Scalable Intervention
Source: J Eur Econ Assoc. 2022 Jan 28;20(4):1395–432. doi: 10.1093/jeea/jvac005 (PMC9372035; doi:10.1093/jeea/jvac005)
Supplement: jvac005_Attanasio_etal_Replication-Data-Code [file jvac005_attanasio_etal_replication-data-code.zip › replication-data-code/output/table-6/_Table_Potential_Dosage_on_cognition.pdf]

```
----------------------------------------------------------------------------------------
> --------------------------------------------------------------------------------
      name:  <unnamed>
       log:  C:/Users/Usurio/Dropbox/Trabajo/Raquel Bernal/Pilot II/Paper/Revision JEE
> A/Final Publication/November 2021/replication-data-code/output/table-6/_Tab
> le_Potential_Dosage_on_cognition.log
  log type:  text
 opened on:   2 Dec 2021, 17:34:39

.
. {
. cd "${root}/output/table-6/"
C:\Users\Usurio\Dropbox\Trabajo\Raquel Bernal\Pilot II\Paper\Revision JEEA\Final Publi
> cation\November 2021\replication-data-code\output\table-6
. cap drop diff_days
. gen diff_days = fecha_encuesta_day - P1_1V16F
(125 missing values generated)
. pwcorr diff_days potential_dosage, sig

             | diff_d~s potent~e
-------------+------------------
   diff_days |   1.0000
             |
             |
potential_~e |  -0.0517   1.0000
             |   0.0592
             |
.
. * A. Matrix definition
. * -------------------------------------------------------------------------------
. {
. mat MAT_tabla = J(1,6,.)
. mat MAT_tabla_s = J(1,6,0)
.
. }
.
. * B. Reduced form estimation, linear form
. * -------------------------------------------------------------------------------
. {
. reg b_total_fac_ potential_dosage ${covs} ${anthro_bl}
>                     ///
>         diff_days i.encuestadorBayley, cluster(CodigoMu)

Linear regression                               Number of obs     =      1,292
                                                F(23, 86)         =      16.65
                                                Prob > F          =     0.0000
                                                R-squared         =     0.2181
                                                Root MSE          =     .91338

                                   (Std. err. adjusted for 87 clusters in CodigoMu)
-------------------------------------------------------------------------------------
                    |               Robust
      b_total_fac_ | Coefficient  std. err.      t    P>|t|     [95% conf. interval]
-------------------+-----------------------------------------------------------------
  potential_dosage |   .0020932   .0007938     2.64   0.010     .0005153    .0036712
              sexo |  -.0595932   .0564645    -1.06   0.294    -.1718409    .0526546
                   |
         CodigoDe |
                2  |  -.1401848   .1015795    -1.38   0.171    -.3421181    .0617486
                3  |   -.138614   .1225096    -1.13   0.261    -.3821551    .1049271
                   |
  datosprevios_cdi |   .0665474   .0893265     0.74   0.458    -.1110279    .2441227
      rango_pob_BL |   .0801631   .0777848     1.03   0.306    -.0744679    .2347942
         indw_alto |   .2014268   .0574173     3.51   0.001     .0872849    .3155687
madresadolescentes |   -.035839   .0553809    -0.65   0.519    -.1459326    .0742546
 puntuaciondirecta |   .0084681    .003824     2.21   0.029     .0008662      .01607
        r_zlen_bl |    .000012   .0188982     0.00   0.999    -.0375564    .0375805
        d_zlen_bl |   -.010298   .1527988    -0.07   0.946     -.314052     .293456
        r_zwei_bl |   .0078876   .0224414     0.35   0.726    -.0367244    .0524996
        d_zwei_bl |  -.0702783   .1692125    -0.42   0.679    -.4066617    .2661051
        diff_days |  -.0005397   .0009542    -0.57   0.573    -.0024365    .0013571
                   |
```

```
 encuestadorBayley |
               P2  |  -.0142357   .1766841    -0.08   0.936     -.365472    .3370007
               P3  |  -.1975012   .1554715    -1.27   0.207    -.5065684     .111566
               P4  |  -.5278315   .1925618    -2.74   0.007    -.9106318   -.1450313
               P5  |  -.3138605   .2075398    -1.51   0.134    -.7264359    .0987148
               P6  |   .4737066   .1832644     2.58   0.011     .1093891    .8380241
               P7  |  -.8913972   .1928439    -4.62   0.000    -1.274758   -.5080363
               P8  |   .0247734   .2140454     0.12   0.908    -.4007349    .4502816
               P9  |   .6595617   .1939708     3.40   0.001     .2739606    1.045163
               S2  |  -.4515117   .2184192    -2.07   0.042    -.8857148   -.0173086
                   |
             _cons |    .100069   .5313715     0.19   0.851    -.9562625      1.1564
------------------------------------------------------------------------------------
.
. *) N
. mat MAT_tabla[1,1]=e(N)
.
. *) Potential dosage
. mat MAT_tabla[1,3]=_b[potential_dosage]*100
. mat MAT_tabla[1,4]=_se[potential_dosage]*100
.
. local p2 = string(2*ttail(e(N)-e(rank),
>                                                   ///
>         abs(_b[potential_dosage]/_se[potential_dosage])),"%5.3f")
. mat MAT_tabla_s[1,3]= (`p2' <= 0.1) + (`p2' <= 0.05) + (`p2' <= 0.01)
. di "p-valor: `p2'"
p-valor: 0.008
.
. *) Effect on average potential dosage
. qui sum potential_dosage if T==1 & e(sample)
. local mean=r(mean)
.
. mat MAT_tabla[1,5]=_b[potential_dosage]*`mean'
. test _b[potential_dosage]*`mean'=0

 ( 1)  80.94491*potential_dosage = 0

       F(  1,    86) =    6.95
            Prob > F =    0.0099
. local p=string(r(p), "%5.3f")
. mat MAT_tabla[1,6]=`p'
. mat MAT_tabla_s[1,5]== (`p' <= 0.1) + (`p' <= 0.05) + (`p' <= 0.01)
.
. }
.
. * C. Table without RW
. * -------------------------------------------------------------------------------
. {
. cd "${root}/output/table-6/"
C:\Users\Usurio\Dropbox\Trabajo\Raquel Bernal\Pilot II\Paper\Revision JEEA\Final Publi
> cation\November 2021\replication-data-code\output\table-6
.
. frmttable using "_Table_Potential_Dosage_on_cognition", replace
>            ///
>         sdec(0,3)
>                                                                     ///
>         statmat(MAT_tabla) substat(1)
>                                               ///
>         annotate(MAT_tabla_s) asymbol("*","**","***")
>                               ///
>         ct("VARIABLES","N","Potential Dosage (SE)",
>                                    ///
>         "Average Effect of Potential Dosage (P value)")
>                               ///
>         rt("Bayley-III Factor"\"")
>                                                       ///
>         note("Note: ***p<0.01; **p<0.05; *p<0.1; 95% confidence interval in parenthe
> sis for two-tailed tests." ///
>         "Standard errors clustered by town; D = beta /SD (Controls)." ///
>         "Covariates included: gender, household wealth index, maternal PPVT score, t
> eenage mother, town's population rang, inverviewer and department FE, and B
> L weight-for-age and height-for-age Z-scores, chilfcare attendance.")
```

```
                                  ------------------------------------------------------
> ------------------------------------------
                                   VARIABLES            N    Potential Dosage (SE)  Ave
> rage Effect of Potential Dosage (P value)
                                  ------------------------------------------------------
> ------------------------------------------
                                   Bayley-III Factor   1,292       0.209***
>               0.169***
                                                                    (0.079)
>               (0.010)
                                  ------------------------------------------------------
> ------------------------------------------
                                   Note: ***p<0.01; **p<0.05; *p<0.1; 95% confidence int
> erval in parenthesis for two-tailed tests.
                                                     Standard errors clustered by town;
> D = beta /SD (Controls).
Covariates included: gender, household wealth index, maternal PPVT score, teenage moth
> er, town's population rang, inverviewer and department FE, and BL weight-fo
> r-age and height-for-age Z-scores, chilfcare attendance.

.
. }
.
. }

. log close
      name:  <unnamed>
       log:  C:/Users/Usurio/Dropbox/Trabajo/Raquel Bernal/Pilot II/Paper/Revision JEE
> A/Final Publication/November 2021/replication-data-code/output/table-6/_Tab
> le_Potential_Dosage_on_cognition.log
  log type:  text
 closed on:   2 Dec 2021, 17:34:39
--------------------------------------------------------------------------------------
> --------------------------------------------------------------------------
```
