# Supplementary material for: Early Stimulation and Nutrition: The Impacts of a Scalable Intervention
Source: J Eur Econ Assoc. 2022 Jan 28;20(4):1395–432. doi: 10.1093/jeea/jvac005 (PMC9372035; doi:10.1093/jeea/jvac005)
Supplement: jvac005_Attanasio_etal_Replication-Data-Code [file jvac005_attanasio_etal_replication-data-code.zip › replication-data-code/output/table-6/dosage.doc]

VARIABLES	N	Potential Dosage (SE)	Average Effect of Potential Dosage (P value)	
Bayley-III Factor	1,292	0.209***	0.169***	
		(0.079)	(0.010)	
