# Supplementary material for: Early Stimulation and Nutrition: The Impacts of a Scalable Intervention
Source: J Eur Econ Assoc. 2022 Jan 28;20(4):1395–432. doi: 10.1093/jeea/jvac005 (PMC9372035; doi:10.1093/jeea/jvac005)
Supplement: jvac005_Attanasio_etal_Replication-Data-Code [file jvac005_attanasio_etal_replication-data-code.zip › replication-data-code/output/table-7/height_heterog.doc]

Table X. Impact on children's outcomes
VARIABLE	Impact (95% CI)	P Value	RW P Value	
Pr(Height-for-age between -5 SD and -1 SD)	-0.0683++	0.0235	0.0440	
	(-0.1263,-0.0102)			
Pr(Height-for-age between -1 SD and 1 SD)	0.0756++	0.0132	0.0332	
	(0.0171,0.1342)			
Pr(Height-for-age between 1 SD and 5 SD)	-0.0008	0.9497	0.9552	
	(-0.0248,0.0233)			
