# Supplementary material for: Early Stimulation and Nutrition: The Impacts of a Scalable Intervention
Source: J Eur Econ Assoc. 2022 Jan 28;20(4):1395–432. doi: 10.1093/jeea/jvac005 (PMC9372035; doi:10.1093/jeea/jvac005)
Supplement: jvac005_Attanasio_etal_Replication-Data-Code [file jvac005_attanasio_etal_replication-data-code.zip › replication-data-code/output/table-8/_Table_Het_Impact_on_cognition_HHcharacteristics.pdf]

```
------------------------------------------------------------------------------------
> ------------------------------------------------------------------------------
      name:  <unnamed>
       log:  C:/Users/Usurio/Dropbox/Trabajo/Raquel Bernal/Pilot II/Paper/Revision JEE
> A/Final Publication/November 2021/replication-data-code/output/table-8/_Tab
> le_Het_Impact_on_cognition_HHcharacteristics.log
  log type:  text
 opened on:   2 Dec 2021, 17:36:23

.
. {
. cd "$root/output/table-8"
C:\Users\Usurio\Dropbox\Trabajo\Raquel Bernal\Pilot II\Paper\Revision JEEA\Final Publi
> cation\November 2021\replication-data-code\output\table-8
.
. * A. Matrix definition
. * -----------------------------------------------------------------------------
. {
. mat MAT_tabla = J(6,4,.)
. mat MAT_tabla_s = J(6,4,0)
. mat N = J(3,2,.)
.
. }
.
. * B. Heterogeneous ITT estimation
. * -----------------------------------------------------------------------------
. {
. *) Maternal education
. het_ols b_total_fac_, tratamiento(T) grupo(escmadre_alto1)
>                      ///
>         filename("Bayley - EscMadre") atricion(atr)
>                                      ///
>         covariates(${covs} ${anthro_bl} i.encuestadorBayley) cluster(CodigoMu)
Pruebas de Dos Colas
Cluster var is group(CodigoMu)
Actual directory is C:\Users\Usurio\Dropbox\Trabajo\Raquel Bernal\Pilot II\Paper\Revis
> ion JEEA\Final Publication\November 2021\replication-data-code\output\table
> -8

                                  --------------------------------------------------
> ----------------------------------------
                                   VARIABLE                     Secundaria o más   Sec
> undaria Incompleta o Menos  Diferencia
                                  --------------------------------------------------
> ----------------------------------------
                                   Total Observaciones = 1331          674
>          657
                                  --------------------------------------------------
> ----------------------------------------
                                              *** Significance at 1%, ** Significance
> at 5%, * Significance at 10%
                                                  () Standard errors in brackets, clus
> tered by group(CodigoMu)

Variable Actual Bayley-III Factor

                          ----------------------------------------------------------
> -------------------------------------------------
                           VARIABLE                             Secundaria o más   Secun
> daria Incompleta o Menos  Diferencia
                          ----------------------------------------------------------
> -------------------------------------------------
                           Total Observaciones = 1331               674
>        657
                           Bayley-III Factor n1=660, n0=632        0.176
>       0.142               0.034     1,292.000
                                                                  (0.075)**
>      (0.097)             (0.115)
                          ----------------------------------------------------------
> -------------------------------------------------
                                              *** Significance at 1%, ** Significance
> at 5%, * Significance at 10%
```

```
                                                () Standard errors in brackets, clus
> tered by group(CodigoMu)

.
. mat MAT_tabla[1,1] = resultados[1,1..2]
. mat MAT_tabla_s[1,2] = resultados_signif[1,2]
.
. mat MAT_tabla[2,1] = resultados[1,3..4]
. mat MAT_tabla_s[2,2] = resultados_signif[1,4]
.
. mat MAT_tabla[1,3] = resultados[1,5..6]
. local p = resultados[1,6]
. mat MAT_tabla_s[1,4] = (`p'<0.1) + (`p'<0.05) + (`p'<0.01)
.
. mat N[1,1] = resultados[1,7..8]
.
. *) Gender
. het_ols b_total_fac_, tratamiento(T) grupo(sexo)
>                             ///
>         filename("Bayley - Gender") atricion(atr)
>                                     ///
>         covariates(${covs} ${anthro_bl} i.encuestadorBayley) cluster(CodigoMu)
Pruebas de Dos Colas
Cluster var is group(CodigoMu)
Actual directory is C:\Users\Usurio\Dropbox\Trabajo\Raquel Bernal\Pilot II\Paper\Revis
> ion JEEA\Final Publication\November 2021\replication-data-code\output\table
> -8

                                                  ----------------------------------
> ----------------------
                                                   VARIABLE                     Male
>   Female  Diferencia
                                                  ----------------------------------
> ----------------------
                                                   Total Observaciones = 1331   690
>    641
                                                  ----------------------------------
> ----------------------
                                            *** Significance at 1%, ** Significance
> at 5%, * Significance at 10%
                                                () Standard errors in brackets, clus
> tered by group(CodigoMu)

Variable Actual Bayley-III Factor

                                       --------------------------------------------
> ----------------------------------
                                        VARIABLE                               Male
>   Female   Diferencia
                                       --------------------------------------------
> ----------------------------------
                                        Total Observaciones = 1331              690
>     641
                                        Bayley-III Factor n1=673, n0=619      0.198
>    0.125     0.074     1,292.000
                                                                           (0.088)**
>   (0.077)   (0.103)
                                       --------------------------------------------
> ----------------------------------
                                            *** Significance at 1%, ** Significance
> at 5%, * Significance at 10%
                                                () Standard errors in brackets, clus
> tered by group(CodigoMu)
```

```
.
. mat MAT_tabla[4,1] = resultados[1,1..2]
. mat MAT_tabla_s[4,2] = resultados_signif[1,2]
.
. mat MAT_tabla[3,1] = resultados[1,3..4]
. mat MAT_tabla_s[3,2] = resultados_signif[1,4]
.
. mat MAT_tabla[3,3] = resultados[1,5..6]
. local p = resultados[1,6]
. mat MAT_tabla_s[3,4] = (`p'<0.1) + (`p'<0.05) + (`p'<0.01)
.
. mat N[2,1] = resultados[1,7..8]
.
. *) Wealth index
. het_ols b_total_fac_, tratamiento(T) grupo(indw_alto)
>                     ///
>         filename("Bayley - Indw") atricion(atr)
>                                   ///
>         covariates(${covs} ${anthro_bl} i.encuestadorBayley) cluster(CodigoMu)
Pruebas de Dos Colas
Cluster var is group(CodigoMu)
Actual directory is C:\Users\Usurio\Dropbox\Trabajo\Raquel Bernal\Pilot II\Paper\Revis
> ion JEEA\Final Publication\November 2021\replication-data-code\output\table
> -8

                                                                 ---------------------------------
> ---------------------
                                                                  VARIABLE                     Alt
> o  Bajo  Diferencia
                                                                 ---------------------------------
> ---------------------
                                                                  Total Observaciones = 1331   675
>    656
                                                                 ---------------------------------
> ---------------------
                                                          *** Significance at 1%, ** Significance
> at 5%, * Significance at 10%
                                                              () Standard errors in brackets, clus
> tered by group(CodigoMu)

Variable Actual Bayley-III Factor

                                                    ---------------------------------------------
> ---------------------------------
                                                     VARIABLE                            Alto
>   Bajo     Diferencia
                                                    ---------------------------------------------
> ---------------------------------
                                                     Total Observaciones = 1331           675
>   656
                                                     Bayley-III Factor n1=657, n0=635    0.042
>  0.285       -0.243    1,292.000
                                                                                        (0.082)  (
> 0.087)***  (0.106)**
                                                    ---------------------------------------------
> ---------------------------------
                                                          *** Significance at 1%, ** Significance
> at 5%, * Significance at 10%
                                                              () Standard errors in brackets, clus
> tered by group(CodigoMu)
```

```
.
. mat MAT_tabla[5,1] = resultados[1,1..2]
. mat MAT_tabla_s[5,2] = resultados_signif[1,2]
.
. mat MAT_tabla[6,1] = resultados[1,3..4]
. mat MAT_tabla_s[6,2] = resultados_signif[1,4]
.
. mat MAT_tabla[5,3] = resultados[1,5..6]
. local p = resultados[1,6]
. mat MAT_tabla_s[5,4] = (`p'<0.1) + (`p'<0.05) + (`p'<0.01)
.
. mat N[3,1] = resultados[1,7..8]
.
. *) Ns locals
. local n = 0
. foreach var of varlist escmadre_alto1 sexo indw_alto{
  2.
.         local ++n
  3.
.         count if encuestadorBayley <. & b_total_fac_ <. & `var' == 1
  4.         local n`n'_1 = r(N)
  5.
.         count if encuestadorBayley <. & b_total_fac_ <. & `var' == 0
  6.         local n`n'_0 = r(N)
  7. }
  660
  632
  673
  619
  657
  635
.
. }
.
. * C. Table without RW
. * ---------------------------------------------------------------------------
. {
. cd "$root/output/table-8"
C:\Users\Usurio\Dropbox\Trabajo\Raquel Bernal\Pilot II\Paper\Revision JEEA\Final Publi
> cation\November 2021\replication-data-code\output\table-8
. frmttable using "_Table_Het_Impact_on_cognition_HHcharacteristics", replace     ///
>         statmat(MAT_tabla) annotate(MAT_tabla_s) asymbol(*,**,***)
>                     ///
>         landscape sdec(3,3) sub(1) dbldiv(,)
>                                     ///
>         ti("Table X. Heterogeneous impacts on the Bayley-III factor by child and hou
> sehold characteristics") ///
>         rt(""\"Maternal education ≥ complete high school (N=`n1_1')"\
>           ///
>         ""\"Maternal education < complete high school (N=`n1_0')"\
>                     ///
>         ""\"Male (N=`n2_1')"\
>                                                     ///
>         ""\"Female (N=`n2_0')"\
>                                                     ///
>         ""\"Wealth index above the median (N=`n3_1')"\
>                             ///
>         ""\"Wealth index below the median (N=`n3_0')")
>                             ///
>         ct("{\b Group (Number of observations)}","{\b ITT}","{\b Estimated}"\
>     ///
>         "","{\b (SE)}","Difference"\
>                                             ///
>         "","","{\b (pvalue)}")
>                                                     ///
>         note("Note: ***p<0.01; **p<0.05; *p<0.1. Standard errors clustered by town.
> Heterogeneous effects estimated by subsamples: Difference is a cross-model
> test for ITT associated parameter. Covariates included: gender, household wealth ind
> ex, maternal PPVT score, teenage mother, town's population rang, inverviewe
> r and department FE, and BL weight-for-age and height-for-age Z-scores, chilfcare at
> tendance.")
```

```
                                 Table X. Heterogeneous impacts on the Bayley-III fact
> or by child and household characteristics
                                       -----------------------------------------------
> -----------------------------------
                                        {\b Group (Number of observations)}
>          {\b ITT}   {\b Estimated}

>         {\b (SE)}     Difference

>                     {\b (pvalue)}
                                       -----------------------------------------------
> -----------------------------------

>           0.176        0.034
                                        Maternal education ≥ complete high school (N=6
> 60)     (0.075)**      (0.115)

>           0.142
                                        Maternal education < complete high school (N=6
> 32)      (0.097)

>           0.125        0.074
                                        Male (N=673)
>          (0.077)       (0.103)

>           0.198
                                        Female (N=619)
>         (0.088)**

>           0.042        -0.243
                                        Wealth index above the median (N=657)
>          (0.082)       (0.106)

>           0.285
                                        Wealth index below the median (N=635)
>         (0.087)***
                                       -----------------------------------------------
> -----------------------------------
Note: ***p<0.01; **p<0.05; *p<0.1. Standard errors clustered by town. Heterogeneous ef
> fects estimated by subsamples: Difference is a cross-model test for ITT ass
> ociated parameter. Covariates included: gender, household wealth index, maternal PPV
> T score, teenage mother, town's population rang, inverviewer and department
>  FE, and BL weight-for-age and height-for-age Z-scores, chilfcare attendance.

.
. }
.
. * D. Romano-Wolf
. * -----------------------------------------------------------------------------
. * Two step RW
. * 1st: bootstrap matrix (of p-values)
. * 2nd: RW p-values' calculation
. {
. cd "$root/output/table-8"
C:\Users\Usurio\Dropbox\Trabajo\Raquel Bernal\Pilot II\Paper\Revision JEEA\Final Publi
> cation\November 2021\replication-data-code\output\table-8
.
. *) 1st. Boostrap matrix
. {
. cd "$root/output/table-8"
C:\Users\Usurio\Dropbox\Trabajo\Raquel Bernal\Pilot II\Paper\Revision JEEA\Final Publi
> cation\November 2021\replication-data-code\output\table-8
```

```
.
.         * New interaction vars
.         foreach var of varlist escmadre_alto1 sexo indw_alto{
  2.                cap drop T_`var'_1 cap drop T_`var'_0
  3.                gen T_`var'_1 = T * `var'
  4.                gen T_`var'_0 = T * (1-`var')
  5.         }
.
.         * Maternal education: Below
.         romwolf_matgen b_total_fac_, indepvar(T)
>                                      ///
>               controls(T_escmadre_alto1_1 escmadre_alto1 ${covs} ${anthro_bl}
>           ///
>               i.encuestadorBayley)
>                                                      ///
>               reps(2500) strata(T) cluster(CodigoMu) seed(1234)
>                            ///
>               outmatfile("Or T MatEd0") outfile("Null T MatEd0")
Two-tailed Tests
Running 2500 bootstrap replications for each variable.  This may take some time
Original regress works for b_total_fac_
Original T is 1.460941895954879 and Or. P Value of b_total_fac_ is 0.1443
Boostrap for b_total_fac_ started at 17:36:23  2 Dec 2021
Bootstrap for b_total_fac_ finished at 17:36:56  2 Dec 2021
file C:\Users\Usurio\Dropbox\Trabajo\Raquel Bernal\Pilot II\Paper\Revision JEEA\Final
> Publication\November 2021\replication-data-code\output\table-8/Null T
    MatEd0.dta saved
file C:\Users\Usurio\Dropbox\Trabajo\Raquel Bernal\Pilot II\Paper\Revision JEEA\Final
> Publication\November 2021\replication-data-code\output\table-8/Or T
    MatEd0.dta saved
Program has finished. Two files were created:
File Null T MatEd0 contains bootstrap matrix in varlist order (size 1 x 2500)
File Or T MatEd0 contains Original T stat and Pvalue by varlist (obs 1 is T and obs 2
> is Pval)
.
.         * Maternal education: Above
.         romwolf_matgen b_total_fac_, indepvar(T)
>                                      ///
>               controls(T_escmadre_alto1_0 escmadre_alto1 ${covs} ${anthro_bl}
>           ///
>               i.encuestadorBayley)
>                                                      ///
>               reps(2500) strata(T) cluster(CodigoMu) seed(1234)
>                            ///
>               outmatfile("Or T MatEd1") outfile("Null T MatEd1")
Two-tailed Tests
Running 2500 bootstrap replications for each variable.  This may take some time
Original regress works for b_total_fac_
Original T is 2.335028269146l8 and Or. P Value of b_total_fac_ is 0.0197
Boostrap for b_total_fac_ started at 17:36:56  2 Dec 2021
Bootstrap for b_total_fac_ finished at 17:37:29  2 Dec 2021
file C:\Users\Usurio\Dropbox\Trabajo\Raquel Bernal\Pilot II\Paper\Revision JEEA\Final
> Publication\November 2021\replication-data-code\output\table-8/Null T
    MatEd1.dta saved
file C:\Users\Usurio\Dropbox\Trabajo\Raquel Bernal\Pilot II\Paper\Revision JEEA\Final
> Publication\November 2021\replication-data-code\output\table-8/Or T
    MatEd1.dta saved
Program has finished. Two files were created:
File Null T MatEd1 contains bootstrap matrix in varlist order (size 1 x 2500)
File Or T MatEd1 contains Original T stat and Pvalue by varlist (obs 1 is T and obs 2
> is Pval)
```

```
.
.         * Maternal education: Difference
.         romwolf_matgen b_total_fac_, indepvar(T_escmadre_alto1_1)
>                     ///
>                 controls(T escmadre_alto1 ${covs} ${anthro_bl}
>                             ///
>                 i.encuestadorBayley)
>                                                         ///
>                 reps(2500) strata(T) cluster(CodigoMu) seed(1234)
>                             ///
>                 outmatfile("Or T MatEdDiff") outfile("Null T MatEdDiff")
Two-tailed Tests
Running 2500 bootstrap replications for each variable.  This may take some time
Original regress works for b_total_fac_
Original T is .2940607924404073 and Or. P Value of b_total_fac_ is 0.7688
Boostrap for b_total_fac_ started at 17:37:29  2 Dec 2021
Bootstrap for b_total_fac_ finished at 17:38:02  2 Dec 2021
file C:\Users\Usurio\Dropbox\Trabajo\Raquel Bernal\Pilot II\Paper\Revision JEEA\Final
> Publication\November 2021\replication-data-code\output\table-8/Null T
    MatEdDiff.dta saved
file C:\Users\Usurio\Dropbox\Trabajo\Raquel Bernal\Pilot II\Paper\Revision JEEA\Final
> Publication\November 2021\replication-data-code\output\table-8/Or T
    MatEdDiff.dta saved
Program has finished. Two files were created:
File Null T MatEdDiff contains bootstrap matrix in varlist order (size 1 x 2500)
File Or T MatEdDiff contains Original T stat and Pvalue by varlist (obs 1 is T and obs
>  2 is Pval)
.
.         * Gender: Female
.         romwolf_matgen b_total_fac_, indepvar(T)
>                                 ///
>                 controls(T_sexo_1 sexo ${covs} ${anthro_bl}
>                             ///
>                 i.encuestadorBayley)
>                                                         ///
>                 reps(2500) strata(T) cluster(CodigoMu) seed(1234)
>                             ///
>                 outmatfile("Or T Gender0") outfile("Null T Gender0")
Two-tailed Tests
Running 2500 bootstrap replications for each variable.  This may take some time
Original regress works for b_total_fac_
Original T is 1.620489299128523 and Or. P Value of b_total_fac_ is 0.1054
Boostrap for b_total_fac_ started at 17:38:02  2 Dec 2021
Bootstrap for b_total_fac_ finished at 17:38:34  2 Dec 2021
file C:\Users\Usurio\Dropbox\Trabajo\Raquel Bernal\Pilot II\Paper\Revision JEEA\Final
> Publication\November 2021\replication-data-code\output\table-8/Null T
    Gender0.dta saved
file C:\Users\Usurio\Dropbox\Trabajo\Raquel Bernal\Pilot II\Paper\Revision JEEA\Final
> Publication\November 2021\replication-data-code\output\table-8/Or T
    Gender0.dta saved
Program has finished. Two files were created:
File Null T Gender0 contains bootstrap matrix in varlist order (size 1 x 2500)
File Or T Gender0 contains Original T stat and Pvalue by varlist (obs 1 is T and obs 2
>  is Pval)
.
.         * Gender: Male
.         romwolf_matgen b_total_fac_, indepvar(T)
>                                 ///
>                 controls(T_sexo_0 sexo ${covs} ${anthro_bl}
>                             ///
>                 i.encuestadorBayley)
>                                                         ///
>                 reps(2500) strata(T) cluster(CodigoMu) seed(1234)
>                             ///
>                 outmatfile("Or T Gender1") outfile("Null T Gender1")
Two-tailed Tests
Running 2500 bootstrap replications for each variable.  This may take some time
Original regress works for b_total_fac_
Original T is 2.244663971250839 and Or. P Value of b_total_fac_ is 0.0250
Boostrap for b_total_fac_ started at 17:38:34  2 Dec 2021
Bootstrap for b_total_fac_ finished at 17:39:07  2 Dec 2021
file C:\Users\Usurio\Dropbox\Trabajo\Raquel Bernal\Pilot II\Paper\Revision JEEA\Final
```

```
> Publication\November 2021\replication-data-code\output\table-8/Null T
    Gender1.dta saved
file C:\Users\Usurio\Dropbox\Trabajo\Raquel Bernal\Pilot II\Paper\Revision JEEA\Final
> Publication\November 2021\replication-data-code\output\table-8/Or T
    Gender1.dta saved
Program has finished. Two files were created:
File Null T Gender1 contains bootstrap matrix in varlist order (size 1 x 2500)
File Or T Gender1 contains Original T stat and Pvalue by varlist (obs 1 is T and obs 2
>  is Pval)
.
.         * Gender: Difference
.         romwolf_matgen b_total_fac_, indepvar(T_sexo_1)
>                               ///
>                 controls(T sexo ${covs} ${anthro_bl}
>                                       ///
>                 i.encuestadorBayley)
>                                                       ///
>                 reps(2500) strata(T) cluster(CodigoMu) seed(1234)
>                               ///
>                 outmatfile("Or T GenderDiff") outfile("Null T GenderDiff")
Two-tailed Tests
Running 2500 bootstrap replications for each variable.  This may take some time
Original regress works for b_total_fac_
Original T is .7159253001615931 and Or. P Value of b_total_fac_ is 0.4742
Boostrap for b_total_fac_ started at 17:39:07  2 Dec 2021
Bootstrap for b_total_fac_ finished at 17:39:40  2 Dec 2021
file C:\Users\Usurio\Dropbox\Trabajo\Raquel Bernal\Pilot II\Paper\Revision JEEA\Final
> Publication\November 2021\replication-data-code\output\table-8/Null T
    GenderDiff.dta saved
file C:\Users\Usurio\Dropbox\Trabajo\Raquel Bernal\Pilot II\Paper\Revision JEEA\Final
> Publication\November 2021\replication-data-code\output\table-8/Or T
    GenderDiff.dta saved
Program has finished. Two files were created:
File Null T GenderDiff contains bootstrap matrix in varlist order (size 1 x 2500)
File Or T GenderDiff contains Original T stat and Pvalue by varlist (obs 1 is T and ob
> s 2 is Pval)
.
.         * Wealth index: Below
.         romwolf_matgen b_total_fac_, indepvar(T)
>                                       ///
>                 controls(T_indw_alto_1 indw_alto ${covs} ${anthro_bl}
>                     ///
>                 i.encuestadorBayley)
>                                                       ///
>                 reps(2500) strata(T) cluster(CodigoMu) seed(1234)
>                               ///
>                 outmatfile("Or T Indw0") outfile("Null T Indw0")
Two-tailed Tests
Running 2500 bootstrap replications for each variable.  This may take some time
Original regress works for b_total_fac_
Original T is 3.267945969202977 and Or. P Value of b_total_fac_ is 0.0011
Boostrap for b_total_fac_ started at 17:39:40  2 Dec 2021
Bootstrap for b_total_fac_ finished at 17:40:13  2 Dec 2021
file C:\Users\Usurio\Dropbox\Trabajo\Raquel Bernal\Pilot II\Paper\Revision JEEA\Final
> Publication\November 2021\replication-data-code\output\table-8/Null T
    Indw0.dta saved
file C:\Users\Usurio\Dropbox\Trabajo\Raquel Bernal\Pilot II\Paper\Revision JEEA\Final
> Publication\November 2021\replication-data-code\output\table-8/Or T
    Indw0.dta saved
Program has finished. Two files were created:
File Null T Indw0 contains bootstrap matrix in varlist order (size 1 x 2500)
File Or T Indw0 contains Original T stat and Pvalue by varlist (obs 1 is T and obs 2 i
> s Pval)
```

```
.
.         * Wealth index: Above
.         romwolf_matgen b_total_fac_, indepvar(T)
>                                       ///
>                 controls(T_indw_alto_0 indw_alto ${covs} ${anthro_bl}
>                     ///
>                 i.encuestadorBayley)
>                                                       ///
>                 reps(2500) strata(T) cluster(CodigoMu) seed(1234)
>                             ///
>                 outmatfile("Or T Indw1") outfile("Null T Indw1")
Two-tailed Tests
Running 2500 bootstrap replications for each variable.  This may take some time
Original regress works for b_total_fac_
Original T is .5084784535416963 and Or. P Value of b_total_fac_ is 0.6112
Boostrap for b_total_fac_ started at 17:40:13  2 Dec 2021
Bootstrap for b_total_fac_ finished at 17:40:45  2 Dec 2021
file C:\Users\Usurio\Dropbox\Trabajo\Raquel Bernal\Pilot II\Paper\Revision JEEA\Final
> Publication\November 2021\replication-data-code\output\table-8/Null T
    Indw1.dta saved
file C:\Users\Usurio\Dropbox\Trabajo\Raquel Bernal\Pilot II\Paper\Revision JEEA\Final
> Publication\November 2021\replication-data-code\output\table-8/Or T
    Indw1.dta saved
Program has finished. Two files were created:
File Null T Indw1 contains bootstrap matrix in varlist order (size 1 x 2500)
File Or T Indw1 contains Original T stat and Pvalue by varlist (obs 1 is T and obs 2 i
> s Pval)
.
.         * Wealth index: Difference
.         romwolf_matgen b_total_fac_, indepvar(T_indw_alto_1)
>                     ///
>                 controls(T indw_alto ${covs} ${anthro_bl}
>                                       ///
>                 i.encuestadorBayley)
>                                                       ///
>                 reps(2500) strata(T) cluster(CodigoMu) seed(1234)
>                             ///
>                 outmatfile("Or T IndwDiff") outfile("Null T IndwDiff")
Two-tailed Tests
Running 2500 bootstrap replications for each variable.  This may take some time
Original regress works for b_total_fac_
Original T is 2.300763809163524 and Or. P Value of b_total_fac_ is 0.0216
Boostrap for b_total_fac_ started at 17:40:45  2 Dec 2021
Bootstrap for b_total_fac_ finished at 17:41:18  2 Dec 2021
file C:\Users\Usurio\Dropbox\Trabajo\Raquel Bernal\Pilot II\Paper\Revision JEEA\Final
> Publication\November 2021\replication-data-code\output\table-8/Null T
    IndwDiff.dta saved
file C:\Users\Usurio\Dropbox\Trabajo\Raquel Bernal\Pilot II\Paper\Revision JEEA\Final
> Publication\November 2021\replication-data-code\output\table-8/Or T
    IndwDiff.dta saved
Program has finished. Two files were created:
File Null T IndwDiff contains bootstrap matrix in varlist order (size 1 x 2500)
File Or T IndwDiff contains Original T stat and Pvalue by varlist (obs 1 is T and obs
> 2 is Pval)
.
. }
.
. *) Matching matrix
. {
. cd "$root/output/table-8"
C:\Users\Usurio\Dropbox\Trabajo\Raquel Bernal\Pilot II\Paper\Revision JEEA\Final Publi
> cation\November 2021\replication-data-code\output\table-8
```

```
.
. preserve
.         foreach name in Gender0 Gender1 GenderDiff Indw0 Indw1 IndwDiff
>             ///
>         MatEd0 MatEd1 MatEdDiff{
  2.                  use "Or T `name'", replace
  3.                  cap confirm variable b_total_fac_`name'
  4.                  if !_rc di " "
  5.                  else {
  6.                          rename b_total_fac_ b_total_fac_`name'
  7.                          save "Or T `name'", replace
  8.                  }
  9.                  cap confirm variable n
 10.                  if !_rc di "Variable n already exist"
 11.                  else {
 12.                          cap gen n = _n
 13.                          save "Or T `name'", replace
 14.                  }
 15.
.                 use "Null T `name'", replace
 16.                  cap confirm variable b_total_fac_`name'
 17.                  if !_rc di " "
 18.                  else {
 19.                          rename b_total_fac_ b_total_fac_`name'
 20.                          save "Null T `name'", replace
 21.                  }
 22.          }
file Or T Gender0.dta saved
file Or T Gender0.dta saved
file Null T Gender0.dta saved
file Or T Gender1.dta saved
file Or T Gender1.dta saved
file Null T Gender1.dta saved
file Or T GenderDiff.dta saved
file Or T GenderDiff.dta saved
file Null T GenderDiff.dta saved
file Or T Indw0.dta saved
file Or T Indw0.dta saved
file Null T Indw0.dta saved
file Or T Indw1.dta saved
file Or T Indw1.dta saved
file Null T Indw1.dta saved
file Or T IndwDiff.dta saved
file Or T IndwDiff.dta saved
file Null T IndwDiff.dta saved
file Or T MatEd0.dta saved
file Or T MatEd0.dta saved
file Null T MatEd0.dta saved
file Or T MatEd1.dta saved
file Or T MatEd1.dta saved
file Null T MatEd1.dta saved
file Or T MatEdDiff.dta saved
file Or T MatEdDiff.dta saved
file Null T MatEdDiff.dta saved
.
.         use "Null T Gender0", replace
.         foreach file in  Gender1 GenderDiff Indw0 Indw1 IndwDiff
>                     ///
>         MatEd0 MatEd1 MatEdDiff{
  2.                  merge 1:1 iter using "Null T `file'"
  3.                  drop _merge
  4.          }

    Result                      Number of obs
    -----------------------------------------
    Not matched                             0
    Matched                             2,500  (_merge==3)
    -----------------------------------------
```

```
    Result                           Number of obs
    -----------------------------------------
    Not matched                                 0
    Matched                                 2,500  (_merge==3)
    -----------------------------------------

    Result                           Number of obs
    -----------------------------------------
    Not matched                                 0
    Matched                                 2,500  (_merge==3)
    -----------------------------------------

    Result                           Number of obs
    -----------------------------------------
    Not matched                                 0
    Matched                                 2,500  (_merge==3)
    -----------------------------------------

    Result                           Number of obs
    -----------------------------------------
    Not matched                                 0
    Matched                                 2,500  (_merge==3)
    -----------------------------------------

    Result                           Number of obs
    -----------------------------------------
    Not matched                                 0
    Matched                                 2,500  (_merge==3)
    -----------------------------------------

    Result                           Number of obs
    -----------------------------------------
    Not matched                                 0
    Matched                                 2,500  (_merge==3)
    -----------------------------------------

    Result                           Number of obs
    -----------------------------------------
    Not matched                                 0
    Matched                                 2,500  (_merge==3)
    -----------------------------------------
.         save "Null Ts", replace
file Null Ts.dta saved
.         use "Or T Gender0", replace
.         foreach file in  Gender1 GenderDiff Indw0 Indw1 IndwDiff
>                    ///
>         MatEd0 MatEd1 MatEdDiff {
  2.                 merge 1:1 n using "Or T `file'"
  3.                 drop _merge
  4.         }

    Result                           Number of obs
    -----------------------------------------
    Not matched                                 0
    Matched                                     2  (_merge==3)
    -----------------------------------------

    Result                           Number of obs
    -----------------------------------------
    Not matched                                 0
    Matched                                     2  (_merge==3)
    -----------------------------------------

    Result                           Number of obs
    -----------------------------------------
    Not matched                                 0
    Matched                                     2  (_merge==3)
    -----------------------------------------
```

```
    Result                      Number of obs
    -----------------------------------------
    Not matched                             0
    Matched                                 2  (_merge==3)
    -----------------------------------------

    Result                      Number of obs
    -----------------------------------------
    Not matched                             0
    Matched                                 2  (_merge==3)
    -----------------------------------------

    Result                      Number of obs
    -----------------------------------------
    Not matched                             0
    Matched                                 2  (_merge==3)
    -----------------------------------------

    Result                      Number of obs
    -----------------------------------------
    Not matched                             0
    Matched                                 2  (_merge==3)
    -----------------------------------------

    Result                      Number of obs
    -----------------------------------------
    Not matched                             0
    Matched                                 2  (_merge==3)
    -----------------------------------------
.         save "Or Ts", replace
file Or Ts.dta saved
.
. restore
.
. }
.
. *) 2nd. RW P Value calculation
. {
. cd "$root/output/table-8"
C:\Users\Usurio\Dropbox\Trabajo\Raquel Bernal\Pilot II\Paper\Revision JEEA\Final Publi
> cation\November 2021\replication-data-code\output\table-8
.
. foreach name in Gender0  Gender1 GenderDiff Indw0 Indw1 IndwDiff
>              ///
> MatEd0 MatEd1 MatEdDiff {
  2.         cap drop b_total_fac_`name'
  3.         gen b_total_fac_`name'=.
  4. }
(1,456 missing values generated)
(1,456 missing values generated)
(1,456 missing values generated)
(1,456 missing values generated)
(1,456 missing values generated)
(1,456 missing values generated)
(1,456 missing values generated)
(1,456 missing values generated)
(1,456 missing values generated)
.
. * Efects by group
. romwolf_matcalc b_total_fac_Gender0 b_total_fac_Gender1
>                      ///
>         b_total_fac_Indw0 b_total_fac_Indw1
>                                 ///
>         b_total_fac_MatEd0 b_total_fac_MatEd1,
>                                 ///
>         inmatfile("Or Ts") infile("Null Ts")
Maximum t among remaining candidates is 3.267946004867554 (variable 3)
Maximum t among remaining candidates is 2.335028171539307 (variable 6)
Maximum t among remaining candidates is 2.244663953781128 (variable 2)
Maximum t among remaining candidates is 1.620489239692688 (variable 1)
Maximum t among remaining candidates is 1.460941910743713 (variable 5)
Maximum t among remaining candidates is .5084784626960754 (variable 4)
```

```
For the variable b_total_fac_Gender0: Original p-value is .1054000034928322. Romano Wo
> lf p-value is 0.2439.
For the variable b_total_fac_Gender1: Original p-value is .025000000372529. Romano Wol
> f p-value is 0.0768.
For the variable b_total_fac_Indw0: Original p-value is .0010999999940395. Romano Wolf
>  p-value is 0.0056.
For the variable b_total_fac_Indw1: Original p-value is .6111999750137329. Romano Wolf
>  p-value is 0.5990.
For the variable b_total_fac_MatEd0: Original p-value is .1442999988794327. Romano Wol
> f p-value is 0.2439.
For the variable b_total_fac_MatEd1: Original p-value is .019700000625849. Romano Wol
> f p-value is 0.0720.
.
. local n = 0
. foreach var of varlist b_total_fac_MatEd1 b_total_fac_MatEd0
>            ///
> b_total_fac_Gender1 b_total_fac_Gender0
>                                     ///
> b_total_fac_Indw1 b_total_fac_Indw0{
  2.          local ++n
  3.
.         local p = e(rw_`var')
  4.          mat MAT_tabla[`n',2] = `p'
  5.          mat MAT_tabla_s[`n',2] = (`p'<0.1) + (`p'<0.05) + (`p'<0.01)
  6. }
.
. * Differential effects
. romwolf_matcalc b_total_fac_GenderDiff
>                                     ///
>         b_total_fac_IndwDiff
>                                                     ///
>         b_total_fac_MatEdDiff,
>                                                     ///
>         inmatfile("Or Ts") infile("Null Ts")
Maximum t among remaining candidates is 2.300763845443726 (variable 2)
Maximum t among remaining candidates is .7159252762794495 (variable 1)
Maximum t among remaining candidates is .2940607964992523 (variable 3)


For the variable b_total_fac_GenderDiff: Original p-value is .4742000102996826. Romano
>  Wolf p-value is 0.7197.
For the variable b_total_fac_IndwDiff: Original p-value is .0216000005602837. Romano W
> olf p-value is 0.0624.
For the variable b_total_fac_MatEdDiff: Original p-value is .7688000202178955. Romano
> Wolf p-value is 0.7605.
.
. local n = 1
. foreach var of varlist b_total_fac_MatEdDiff
>                             ///
> b_total_fac_GenderDiff b_total_fac_IndwDiff{
  2.
.         di "var `n'"
  3.          local p = e(rw_`var')
  4.          di "a"
  5.          mat MAT_tabla[`n',4] = `p'
  6.          di "b"
  7.          mat MAT_tabla_s[`n',4] = (`p'<0.1) + (`p'<0.05) + (`p'<0.01)
  8.          di "c"
  9.          local ++n
 10.          local ++n
 11. }
var 1
a
b
c
var 3
a
b
c
var 5
```

```
a
b
c
.
. }
.
. }
.
. *) Ns locals
. local n = 0
. foreach var of varlist escmadre_alto1 sexo indw_alto{
  2.
.         local ++n
  3.
.         count if encuestadorBayley <. & b_total_fac_ <. & `var' == 1
  4.         local n`n'_1 = r(N)
  5.
.         count if encuestadorBayley <. & b_total_fac_ <. & `var' == 0
  6.         local n`n'_0 = r(N)
  7. }
  660
  632
  673
  619
  657
  635
.
. * E. Table with RW
. * ---------------------------------------------------------------------------------
. {
. cd "$root/output/table-8"
C:\Users\Usurio\Dropbox\Trabajo\Raquel Bernal\Pilot II\Paper\Revision JEEA\Final Publi
> cation\November 2021\replication-data-code\output\table-8
.
. frmttable using "_Table_Het_Impact_on_cognition_HHcharacteristics_RW", replace  ///
>         statmat(MAT_tabla) annotate(MAT_tabla_s) asymbol(*,**,***)
>                     ///
>         landscape sdec(3,3) sub(1) dbldiv(,)
>                                     ///
>         ti("Table X. Heterogeneous impacts on the Bayley-III factor by child and hou
> sehold characteristics") ///
>         rt(""\"Maternal education ≥ complete high school (N=`n1_1')"\
>           ///
>         ""\"Maternal education < complete high school (N=`n1_0')"\
>                     ///
>         ""\"Male (N=`n2_0')"\
>                                                     ///
>         ""\"Female (N=`n2_1')"\
>                                                     ///
>         ""\"Wealth index above the median (N=`n3_1')"\
>                             ///
>         ""\"Wealth index below the median (N=`n3_0')")
>                             ///
>         ct("{\b Group (Number of observations)}","{\b ITT}","{\b Estimated}"\
>     ///
>         "","{\b (RW pvalue)}","{\b Difference}"\
>                                     ///
>         "","","{\b (RW pvalue)}")
>                                                     ///
>         note("Note: ***p<0.01; **p<0.05; *p<0.1. Standard errors clustered by town.
> Heterogeneous effects estimated by subsamples: Difference is a cross-model
> test for ITT associated parameter. Covariates included: gender, household wealth ind
> ex, maternal PPVT score, teenage mother, town's population rang, inverviewe
> r and department FE, and BL weight-for-age and height-for-age Z-scores, chilfcare at
> tendance. P-values adjusted by Romano Wolf (2005, 2016) for 6 multiple hypo
> theses.")
```

```
                                Table X. Heterogeneous impacts on the Bayley-III fact
> or by child and household characteristics
                                  --------------------------------------------------
> ----------------------------------------
                                   {\b Group (Number of observations)}
>         {\b ITT}       {\b Estimated}

>     {\b (RW pvalue)}  {\b Difference}

>                      {\b (RW pvalue)}
                                  --------------------------------------------------
> ----------------------------------------

>          0.176            0.034
                                   Maternal education ≥ complete high school (N=660)
>         (0.072)*          (0.760)

>          0.142
                                   Maternal education < complete high school (N=632)
>         (0.244)

>          0.125            0.074
                                   Male (N=619)
>         (0.077)*          (0.720)

>          0.198
                                   Female (N=673)
>         (0.244)

>          0.042           -0.243
                                   Wealth index above the median (N=657)
>         (0.599)          (0.062)*

>          0.285
                                   Wealth index below the median (N=635)
>        (0.006)***
                                  --------------------------------------------------
> ----------------------------------------
Note: ***p<0.01; **p<0.05; *p<0.1. Standard errors clustered by town. Heterogeneous ef
> fects estimated by subsamples: Difference is a cross-model test for ITT ass
> ociated parameter. Covariates included: gender, household wealth index, maternal PPV
> T score, teenage mother, town's population rang, inverviewer and department
>  FE, and BL weight-for-age and height-for-age Z-scores, chilfcare attendance. P-valu
> es adjusted by Romano Wolf (2005, 2016) for 6 multiple hypotheses.

.
. }
.
. }

. log close
      name:  <unnamed>
       log:  C:/Users/Usurio/Dropbox/Trabajo/Raquel Bernal/Pilot II/Paper/Revision JEE
> A/Final Publication/November 2021/replication-data-code/output/table-8/_Tab
> le_Het_Impact_on_cognition_HHcharacteristics.log
  log type:  text
 closed on:   2 Dec 2021, 17:41:19
--------------------------------------------------------------------------------------
> --------------------------------------------------------------------------
```
