# Supplementary material for: Early Stimulation and Nutrition: The Impacts of a Scalable Intervention
Source: J Eur Econ Assoc. 2022 Jan 28;20(4):1395–432. doi: 10.1093/jeea/jvac005 (PMC9372035; doi:10.1093/jeea/jvac005)
Supplement: jvac005_Attanasio_etal_Replication-Data-Code [file jvac005_attanasio_etal_replication-data-code.zip › replication-data-code/output/table-8/_Table_Het_Impact_on_cognition_HHcharacteristics_RW.doc]

Table X. Heterogeneous impacts on the Bayley-III factor by child and household characteristics
Group (Number of observations)	ITT	Estimated	
	(RW pvalue)	Difference	
		(RW pvalue)	
	0.176	0.034	
Maternal education â¥ complete high school (N=660)	(0.072)*	(0.760)	
	0.142		
Maternal education < complete high school (N=632)	(0.244)		
	0.125	0.074	
Male (N=619)	(0.077)*	(0.720)	
	0.198		
Female (N=673)	(0.244)		
	0.042	-0.243	
Wealth index above the median (N=657)	(0.599)	(0.062)*	
	0.285		
Wealth index below the median (N=635)	(0.006)***		
Note: ***p<0.01; **p<0.05; *p<0.1. Standard errors clustered by town. Heterogeneous effects estimated by subsamples: Difference is a cross-model test for ITT associated parameter. Covariates included: gender, household wealth index, maternal PPVT score, teenage mother, town's population rang, inverviewer and department FE, and BL weight-for-age and height-for-age Z-scores, chilfcare attendance. P-values adjusted by Romano Wolf (2005, 2016) for 6 multiple hypotheses.
