# Supplementary material for: Early Stimulation and Nutrition: The Impacts of a Scalable Intervention
Source: J Eur Econ Assoc. 2022 Jan 28;20(4):1395–432. doi: 10.1093/jeea/jvac005 (PMC9372035; doi:10.1093/jeea/jvac005)
Supplement: jvac005_Attanasio_etal_Replication-Data-Code [file jvac005_attanasio_etal_replication-data-code.zip › replication-data-code/output/table-8/Bayley - EscMadre.doc]

VARIABLE	Secondary or above	Less than secondary	Diferencia		
Total Observaciones = 1331	674	657			
Bayley-III Factor n1=660, n0=632	0.176	0.142	0.034	1,292.000	
	(0.075)**	(0.097)	(0.115)		
*** Significance at 1%, ** Significance at 5%, * Significance at 10%
() Standard errors in brackets, clustered by Fake Municipality ID (bl)
