# Supplementary material for: Early Stimulation and Nutrition: The Impacts of a Scalable Intervention
Source: J Eur Econ Assoc. 2022 Jan 28;20(4):1395–432. doi: 10.1093/jeea/jvac005 (PMC9372035; doi:10.1093/jeea/jvac005)
Supplement: jvac005_Attanasio_etal_Replication-Data-Code [file jvac005_attanasio_etal_replication-data-code.zip › replication-data-code/output/table-8/Bayley - Gender.doc]

VARIABLE	Male	Female	Diferencia		
Total Observaciones = 1331	690	641			
Bayley-III Factor n1=673, n0=619	0.198	0.125	0.074	1,292.000	
	(0.088)**	(0.077)	(0.103)		
*** Significance at 1%, ** Significance at 5%, * Significance at 10%
() Standard errors in brackets, clustered by Fake Municipality ID (bl)
