# Supplementary material for: Early Stimulation and Nutrition: The Impacts of a Scalable Intervention
Source: J Eur Econ Assoc. 2022 Jan 28;20(4):1395–432. doi: 10.1093/jeea/jvac005 (PMC9372035; doi:10.1093/jeea/jvac005)
Supplement: jvac005_Attanasio_etal_Replication-Data-Code [file jvac005_attanasio_etal_replication-data-code.zip › replication-data-code/output/table-8/Bayley - Indw.doc]

VARIABLE	Yes	No	Diferencia		
Total Observaciones = 1331	675	656			
Bayley-III Factor n1=657, n0=635	0.042	0.285	-0.243	1,292.000	
	(0.082)	(0.087)***	(0.106)**		
*** Significance at 1%, ** Significance at 5%, * Significance at 10%
() Standard errors in brackets, clustered by Fake Municipality ID (bl)
