# Supplementary material for: Early Stimulation and Nutrition: The Impacts of a Scalable Intervention
Source: J Eur Econ Assoc. 2022 Jan 28;20(4):1395–432. doi: 10.1093/jeea/jvac005 (PMC9372035; doi:10.1093/jeea/jvac005)
Supplement: jvac005_Attanasio_etal_Replication-Data-Code [file jvac005_attanasio_etal_replication-data-code.zip › replication-data-code/output/table-8/cog_heterog.doc]

Group (Number of observations)	ITT	Estimated	
	(RW pvalue)	Difference	
		(RW pvalue)	
	0.176	0.034	
Maternal education â¥ complete high school (N=660)	(0.072)+	(0.757)	
	0.142		
Maternal education < complete high school (N=632)	(0.234)		
	0.198	0.074	
Male (N=673)	(0.077)+	(0.717)	
	0.125		
Female (N=619)	(0.231)		
	0.042	-0.243	
Wealth index above the median (N=657)	(0.592)	(0.060)+	
	0.285		
Wealth index below the median (N=635)	(0.008)+++		
