# Supplementary material for: Early Stimulation and Nutrition: The Impacts of a Scalable Intervention
Source: J Eur Econ Assoc. 2022 Jan 28;20(4):1395–432. doi: 10.1093/jeea/jvac005 (PMC9372035; doi:10.1093/jeea/jvac005)
Supplement: jvac005_Attanasio_etal_Replication-Data-Code [file jvac005_attanasio_etal_replication-data-code.zip › replication-data-code/output/table-9/_Table_Impact_on_intermediate_outcomes.doc]

Table X. Program impacts on intermediate outcomes
VARIABLE	Beta (95% CI)	P Value	RW P Value	
FCI Home Environment Quality	0.340	0.000***		
	(0.207,0.472)			
Parental Knowledge (Raw Score)	-0.016	0.831		
	(-0.160,0.128)			
Maternal Self-Efficacy (Raw Score)	0.039	0.604		
	(-0.108,0.186)			
ELCSA Food Insecurity Status	-0.089	0.220		
	(-0.231,0.052)			
Note: ***p<0.01; **p<0.05; *p<0.1; 95% confidence interval in parenthesis for two-tailed tests.
Standard errors clustered by town; D = beta /SD (Controls). P values are computed using Romano-Wolf (2005, 2016) step-down procedure. We consider 3 hypotheses for children outcomes.
Covariates included: gender, household wealth index, maternal PPVT score, teenage mother, town's population rang, inverviewer and department FE, and BL weight-for-age and height-for-age Z-scores, childcare attendance.
