# Supplementary material for: Early Stimulation and Nutrition: The Impacts of a Scalable Intervention
Source: J Eur Econ Assoc. 2022 Jan 28;20(4):1395–432. doi: 10.1093/jeea/jvac005 (PMC9372035; doi:10.1093/jeea/jvac005)
Supplement: jvac005_Attanasio_etal_Replication-Data-Code [file jvac005_attanasio_etal_replication-data-code.zip › replication-data-code/output/table-9/_Table_Impact_on_intermediate_outcomes.pdf]

```
-------------------------------------------------------------------------------------
> ------------------------------------------------------------------------------
      name:  <unnamed>
       log:  C:/Users/Usurio/Dropbox/Trabajo/Raquel Bernal/Pilot II/Paper/Revision JEE
> A/Final Publication/November 2021/replication-data-code/output/table-9/_Tab
> le_Impact_on_intermediate_outcomes.log
  log type:  text
 opened on:   2 Dec 2021, 17:41:19

.
. {
. cd "$root/output/table-9"
C:\Users\Usurio\Dropbox\Trabajo\Raquel Bernal\Pilot II\Paper\Revision JEEA\Final Publi
> cation\November 2021\replication-data-code\output\table-9
.
. * A. Matrix definition and new vars
. * ----------------------------------------------------------------------------------
. {
. *) Matrix definition
. mat MAT_tabla = J(4,7,.)
. mat MAT_tabla_s = J(4,7,0)
. mat ci = (0,0,1,0,0,0,0,0,0)
.
. *) New vars
. foreach var of varlist FCI_conjunto know_total auto_eficacia2 inseguridad_bin {
  2.         qui sum `var' if T == 0
  3.         gen `var'_ = (`var'- r(mean))/r(sd)
  4. }
(125 missing values generated)
(125 missing values generated)
(125 missing values generated)
(125 missing values generated)
.
. ren (FCI_conjunto_bl auto_eficacia2_bl inseguridad_bin2_bl)
>                  ///
>         (FCI_conjunto__bl auto_eficacia2__bl inseguridad_bin__bl)
.
. }
.
. * B. ITT estimation
. * ----------------------------------------------------------------------------------
. {
. table_bmj FCI_conjunto_ know_total_ auto_eficacia2_ inseguridad_bin_,
>     ///
>         tratamiento(T) ols filename("Intermediate OLS")
>                                ///
>         blvar covariates(${covs} i.encuestadorM2) cluster(CodigoMu)
Actual directory is C:\Users\Usurio\Dropbox\Trabajo\Raquel Bernal\Pilot II\Paper\Revis
> ion JEEA\Final Publication\November 2021\replication-data-code\output\table
> -9
Pruebas de Dos Colas
Cluster var is group(CodigoMu)
N mero de variables a exportar = 4
Variable Actual FCI_conjunto_
Acaba FCI_conjunto_
Variable Actual know_total_
Acaba know_total_
Variable Actual auto_eficacia2_
Acaba auto_eficacia2_
Variable Actual inseguridad_bin_
Acaba inseguridad_bin_
(1,456 missing values generated)
(1,456 missing values generated)
(1 real change made)
(1 real change made)
(1 real change made)
(1 real change made)
(1 real change made)
(1 real change made)
(1 real change made)
(1 real change made)
```

```
                                                 -------------------------------------
> ------------------------
                                                  VARIABLE   n1=626, n0=705   Beta (95
> % CI)   P Value    D
                                                 -------------------------------------
> ------------------------
                                                  FCI_conjunto_                   0.34
> 0        0.000   0.340
                                                                              (0.207,0
> .472)     ***
                                                  know_total_                     -0.0
> 16       0.831   -0.016
                                                                              (-0.160,
> 0.128)
                                                  auto_eficacia2_                 0.03
> 9        0.604   0.039
                                                                              (-0.108,
> 0.186)
                                                  inseguridad_bin_                -0.0
> 89       0.220   -0.089
                                                                              (-0.231,
> 0.052)
                                                 -------------------------------------
> ------------------------
                                                           Standard Errors Clustered by
>  group(CodigoMu).
                          D=( /SD controls), where SD controls is standard deviatio
> n for control group within estimation sample.
        Covariates Included: , Gender, group(CodigoDe), Indice Riqueza - Por encima
> de la mediana (mayor o igual), Teenage Mother, Mother's TVIP. BL

.
. mat MAT_tabla[1,1] = resultados[1..4,1..5]
. mat MAT_tabla_s[1,3] = resultados_signif[1..4,4]
. }
.
. * C. Table without RW
. * ---------------------------------------------------------------------------
. {
. cd "$root/output/table-9"
C:\Users\Usurio\Dropbox\Trabajo\Raquel Bernal\Pilot II\Paper\Revision JEEA\Final Publi
> cation\November 2021\replication-data-code\output\table-9
. frmttable using "_Table_Impact_on_intermediate_outcomes", replace
>            ///
>        statmat(MAT_tabla) annotate(MAT_tabla_s) asymbol(*,**,***)
>                ///
>        doubles(ci) landscape sdec(3,3) sub(1) dbldiv(,)
>                    ///
>        ti("Table X. Program impacts on intermediate outcomes")
>                ///
>        rt("FCI Home Environment Quality"\""\
>                        ///
>        "Parental Knowledge (Raw Score)"\""\
>                        ///
>        "Maternal Self-Efficacy (Raw Score)"\""\
>                        ///
>        "ELCSA Food Insecurity Status")
>                            ///
>        ct("{\b VARIABLE}","{\b Beta (95% CI)}","{\b P Value}","{\b RW P Value}")
>    ///
>        note("Note: ***p<0.01; **p<0.05; *p<0.1; 95% confidence interval in parenthe
> sis for two-tailed tests." ///
>        "Standard errors clustered by town; D = beta /SD (Controls). P values are co
> mputed using Romano-Wolf (2005, 2016) step-down procedure. We consider 3 hy
> potheses for children outcomes." ///
>        "Covariates included: gender, household wealth index, maternal PPVT score, t
> eenage mother, town's population rang, inverviewer and department FE, and B
> L weight-for-age and height-for-age Z-scores, childcare attendance.")
```

```
                                                          Table X. Program impacts on in
> termediate outcomes
                                    --------------------------------------------------
> ---------------------------------------
                                     {\b VARIABLE}                        {\b Beta (95
> % CI)}  {\b P Value}  {\b RW P Value}
                                    --------------------------------------------------
> ---------------------------------------
                                     FCI Home Environment Quality              0.340
>          0.000***
                                                                          (0.207,0.4
> 72)
                                     Parental Knowledge (Raw Score)            -0.016
>           0.831
                                                                          (-0.160,0.
> 128)
                                     Maternal Self-Efficacy (Raw Score)        0.039
>           0.604
                                                                          (-0.108,0.
> 186)
                                     ELCSA Food Insecurity Status              -0.089
>           0.220
                                                                          (-0.231,0.
> 052)
                                    --------------------------------------------------
> ---------------------------------------
                                Note: ***p<0.01; **p<0.05; *p<0.1; 95% confidence int
> erval in parenthesis for two-tailed tests.
Standard errors clustered by town; D = beta /SD (Controls). P values are computed usin
> g Romano-Wolf (2005, 2016) step-down procedure. We consider 3 hypotheses fo
> r children outcomes.
Covariates included: gender, household wealth index, maternal PPVT score, teenage moth
> er, town's population rang, inverviewer and department FE, and BL weight-fo
> r-age and height-for-age Z-scores, childcare attendance.

. }
.
. * D. Romano-Wolf
. * ---------------------------------------------------------------------------------
. * Two step RW
. * 1st: bootstrap matrix (of p-values)
. * 2nd: RW p-values' calculation
. {
. cd "$root/output/table-9"
C:\Users\Usurio\Dropbox\Trabajo\Raquel Bernal\Pilot II\Paper\Revision JEEA\Final Publi
> cation\November 2021\replication-data-code\output\table-9
.
. *) 1st. Boostrap matrix
. {
. romwolf_matgen FCI_conjunto_ know_total_ auto_eficacia2_ inseguridad_bin_,
>     ///
>         indepvar(T) controls(${covs} i.encuestadorM2) blvar
>                 ///
>         reps(2500) strata(T) cluster(CodigoMu) seed(1234)
>                         ///
>         outmatfile("Or T Int OLS") outfile("Null T Int OLS")
Two-tailed Tests
Running 2500 bootstrap replications for each variable.  This may take some time
Original regress works for FCI_conjunto_
Original T is 5.031745022749456 and Or. P Value of FCI_conjunto_ is 0.0000
Boostrap for FCI_conjunto_ started at 17:41:19  2 Dec 2021
Bootstrap for FCI_conjunto_ finished at 17:41:52  2 Dec 2021
Original regress works for know_total_
Original T is .2137710947951597 and Or. P Value of know_total_ is 0.8308
Boostrap for know_total_ started at 17:41:52  2 Dec 2021
Bootstrap for know_total_ finished at 17:42:25  2 Dec 2021
Original regress works for auto_eficacia2_
Original T is .52078190945314 and Or. P Value of auto_eficacia2_ is 0.6026
Boostrap for auto_eficacia2_ started at 17:42:25  2 Dec 2021
Bootstrap for auto_eficacia2_ finished at 17:42:58  2 Dec 2021
Original regress works for inseguridad_bin_
Original T is 1.235102890763721 and Or. P Value of inseguridad_bin_ is 0.2170
```

```
Boostrap for inseguridad_bin_ started at 17:42:58  2 Dec 2021
Bootstrap for inseguridad_bin_ finished at 17:43:31  2 Dec 2021
file C:\Users\Usurio\Dropbox\Trabajo\Raquel Bernal\Pilot II\Paper\Revision JEEA\Final
> Publication\November 2021\replication-data-code\output\table-9/Null T Int
    OLS.dta saved
file C:\Users\Usurio\Dropbox\Trabajo\Raquel Bernal\Pilot II\Paper\Revision JEEA\Final
> Publication\November 2021\replication-data-code\output\table-9/Or T Int
    OLS.dta saved
Program has finished. Two files were created:
File Null T Int OLS contains bootstrap matrix in varlist order (size 4 x 2500)
File Or T Int OLS contains Original T stat and Pvalue by varlist (obs 1 is T and obs 2
>  is Pval)
. }
.
. *) 2nd. RW P Value calculation
. {
. cd "$root/output/table-9"
C:\Users\Usurio\Dropbox\Trabajo\Raquel Bernal\Pilot II\Paper\Revision JEEA\Final Publi
> cation\November 2021\replication-data-code\output\table-9
.
. romwolf_matcalc FCI_conjunto_ know_total_ auto_eficacia2_ inseguridad_bin_,     ///
>         inmatfile("Or T Int OLS") infile("Null T Int OLS")
Maximum t among remaining candidates is 5.031744956970215 (variable 1)
Maximum t among remaining candidates is 1.235102891921997 (variable 4)
Maximum t among remaining candidates is .520781934261322 (variable 3)
Maximum t among remaining candidates is .2137710899114609 (variable 2)


For the variable FCI_conjunto_: Original p-value is 0. Romano Wolf p-value is 0.0000.
For the variable know_total_: Original p-value is .8307999968528748. Romano Wolf p-val
> ue is 0.8357.
For the variable auto_eficacia2_: Original p-value is .6025999784469604. Romano Wolf p
> -value is 0.8233.
For the variable inseguridad_bin_: Original p-value is .2169999927282333. Romano Wolf
> p-value is 0.4970.
.
. local n = 0
. foreach var of varlist FCI_conjunto_ know_total_ auto_eficacia2_ inseguridad_bin_{
  2.         local ++n
  3.
.         local p = e(rw_`var')
  4.         mat MAT_tabla[`n',6] = `p'
  5.         mat MAT_tabla_s[`n',5] = (`p'<0.1) + (`p'<0.05) + (`p'<0.01)
  6.
. }
.
. }
.
. }
.
. * E. Table with RW
. * ------------------------------------------------------------------------------
. {
. cd "$root/output/table-9"
C:\Users\Usurio\Dropbox\Trabajo\Raquel Bernal\Pilot II\Paper\Revision JEEA\Final Publi
> cation\November 2021\replication-data-code\output\table-9
.
. frmttable using "_Table_Impact_on_intermediate_outcomes_RW", replace
>             ///
>         statmat(MAT_tabla) annotate(MAT_tabla_s) asymbol(*,**,***)
>                    ///
>         doubles(ci) landscape sdec(3,3) sub(1) dbldiv(,)
>                            ///
>         ti("Table X. Program impacts on intermediate outcomes")
>                    ///
>         rt("FCI Home Environment Quality"\""\
>                                    ///
>         "Parental Knowledge (Raw Score)"\""\
>                                    ///
>         "Maternal Self-Efficacy (Raw Score)"\""\
>                                    ///
>         "ELCSA Food Insecurity Status")
```

```
>                                              ///
>          ct("{\b VARIABLE}","{\b Beta (95% CI)}","{\b P Value}","{\b RW P Value}")
>     ///
>          note("Note: ***p<0.01; **p<0.05; *p<0.1; 95% confidence interval in parenthe
> sis for two-tailed tests." ///
>          "Standard errors clustered by town; D = beta /SD (Controls). P values are co
> mputed using Romano-Wolf (2005, 2016) step-down procedure. We consider 3 hy
> potheses for children outcomes." ///
>          "Covariates included: gender, household wealth index, maternal PPVT score, t
> eenage mother, town's population rang, inverviewer and department FE, and B
> L weight-for-age and height-for-age Z-scores, childcare attendance.")

                                                                Table X. Program impacts on in
> termediate outcomes
                                     --------------------------------------------------
> --------------------------------------
                                      {\b VARIABLE}                        {\b Beta (95
> % CI)}  {\b P Value}  {\b RW P Value}
                                     --------------------------------------------------
> --------------------------------------
                                      FCI Home Environment Quality              0.340
>          0.000***       0.000***
                                                                             (0.207,0.4
> 72)
                                      Parental Knowledge (Raw Score)           -0.016
>           0.831           0.836
                                                                             (-0.160,0.
> 128)
                                      Maternal Self-Efficacy (Raw Score)        0.039
>           0.604           0.823
                                                                             (-0.108,0.
> 186)
                                      ELCSA Food Insecurity Status             -0.089
>           0.220           0.497
                                                                             (-0.231,0.
> 052)
                                     --------------------------------------------------
> --------------------------------------
                                   Note: ***p<0.01; **p<0.05; *p<0.1; 95% confidence int
> erval in parenthesis for two-tailed tests.
Standard errors clustered by town; D = beta /SD (Controls). P values are computed usin
> g Romano-Wolf (2005, 2016) step-down procedure. We consider 3 hypotheses fo
> r children outcomes.
Covariates included: gender, household wealth index, maternal PPVT score, teenage moth
> er, town's population rang, inverviewer and department FE, and BL weight-fo
> r-age and height-for-age Z-scores, childcare attendance.

.
. }
.
.
. }

. log close
      name:  <unnamed>
       log:  C:/Users/Usurio/Dropbox/Trabajo/Raquel Bernal/Pilot II/Paper/Revision JEE
> A/Final Publication/November 2021/replication-data-code/output/table-9/_Tab
> le_Impact_on_intermediate_outcomes.log
  log type:  text
 closed on:   2 Dec 2021, 17:43:32
--------------------------------------------------------------------------------------
> ------------------------------------------------------------------------
```
