# Supplementary material for: Early Stimulation and Nutrition: The Impacts of a Scalable Intervention
Source: J Eur Econ Assoc. 2022 Jan 28;20(4):1395–432. doi: 10.1093/jeea/jvac005 (PMC9372035; doi:10.1093/jeea/jvac005)
Supplement: jvac005_Attanasio_etal_Replication-Data-Code [file jvac005_attanasio_etal_replication-data-code.zip › replication-data-code/output/table-9/Intermediate OLS.doc]

VARIABLE   n1=626, n0=705	Beta (95% CI)	P Value	D	
FCI_conjunto_	0.340	0.000	0.340	
	(0.207,0.472)	***		
know_total_	-0.016	0.831	-0.016	
	(-0.160,0.128)			
auto_eficacia2_	0.039	0.604	0.039	
	(-0.108,0.186)			
inseguridad_bin_	-0.089	0.220	-0.089	
	(-0.231,0.052)			
Standard Errors Clustered by Fake Municipality ID (bl).
D=(ß/SD controls), where SD controls is standard deviation for control group within estimation sample.
Covariates Included: , Gender: Male, Fake Department ID, Previous attendance to a child care center (bl), Municipality's population category (bl), Household wealth index above the median (bl), Teenage mother (bl), Mother's PPVT (bl), Interviewer section 2 (fu)
