# Supplementary material for: Early Stimulation and Nutrition: The Impacts of a Scalable Intervention
Source: J Eur Econ Assoc. 2022 Jan 28;20(4):1395–432. doi: 10.1093/jeea/jvac005 (PMC9372035; doi:10.1093/jeea/jvac005)
Supplement: jvac005_Attanasio_etal_Replication-Data-Code [file jvac005_attanasio_etal_replication-data-code.zip › replication-data-code/output/table-9/itt_intermediate.doc]

VARIABLE	Beta (95% CI)	P Value	RW P Value	
FCI Home Environment Quality	0.340	0.000+++	0.000+++	
	(0.207,0.472)			
Parental Knowledge (Raw Score)	-0.016	0.831	0.828	
	(-0.160,0.128)			
Maternal Self-Efficacy (Raw Score)	0.039	0.604	0.828	
	(-0.108,0.186)			
ELCSA Food Insecurity Status	-0.089	0.220	0.496	
	(-0.231,0.052)			
