# Supplementary material for: Early Stimulation and Nutrition: The Impacts of a Scalable Intervention
Source: J Eur Econ Assoc. 2022 Jan 28;20(4):1395–432. doi: 10.1093/jeea/jvac005 (PMC9372035; doi:10.1093/jeea/jvac005)
Supplement: jvac005_Attanasio_etal_Replication-Data-Code [file jvac005_attanasio_etal_replication-data-code.zip › replication-data-code/output/table-d1/Signal_to_noise.doc]

Measurement	Signal	
Bayley-III factor		
Bayley: Cognitive	0.498	
Bayley: Receptive language	0.627	
Bayley: Expressive language	0.464	
Bayley: Fine motor	0.416	
Bayley: Gross motor	0.327	
Parental investment		
FCI: Number of toy sources	0.161	
FCI: Number of types of play materials	0.697	
FCI: Number of types of play activities in last 3 days	0.704	
FCI: Number of books, magazines and newspapers	0.332	
