# Supplementary material for: Early Stimulation and Nutrition: The Impacts of a Scalable Intervention
Source: J Eur Econ Assoc. 2022 Jan 28;20(4):1395–432. doi: 10.1093/jeea/jvac005 (PMC9372035; doi:10.1093/jeea/jvac005)
Supplement: jvac005_Attanasio_etal_Replication-Data-Code [file jvac005_attanasio_etal_replication-data-code.zip › replication-data-code/output/table-e1/caract.doc]

VARIABLE	Treatment	Control	Treatment - Control		
Total Observaciones = 340	171	169	Differencia	p-value	
Age (years) n1=171, n0=169 	41.789	41.408	0.381	0.790	
	(10.041)	(10.367)	(1.428)		
Education (years) n1=171, n0=169 	13.263	13.000	0.263	0.378	
	(1.661)	(1.967)	(0.299)		
Experience (years) n1=171, n0=167 	11.706	11.884	-0.178	0.856	
	(7.974)	(8.519)	(0.979)		
Number of children n1=171, n0=169 	2.713	2.550	0.163	0.307	
	(1.357)	(1.500)	(0.160)		
MC's household size n1=171, n0=169 	3.936	3.947	-0.011	0.949	
	(1.480)	(1.432)	(0.175)		
Number of children (0-12 months) attending n1=171, n0=169 	4.865	5.118	-0.253	0.504	
	(2.058)	(2.291)	(0.379)		
Number of pregnant women attending n1=171, n0=169 	1.860	1.959	-0.099	0.588	
	(1.343)	(1.445)	(0.183)		
Number of group sessions held last month n1=169, n0=167 	5.456	5.090	0.366	0.608	
	(4.500)	(3.395)	(0.713)		
Number of home visits held last month n1=170, n0=166 	12.047	13.512	-1.465	0.209	
	(6.661)	(7.127)	(1.167)		
Hours devoted to planning activities n1=168, n0=169 	4.952	6.858	-1.906	0.013**	
	(3.021)	(6.928)	(0.768)		
PPVT z-score n1=171, n0=169 	0.163	-0.165	0.328	0.062*	
	(1.028)	(0.946)	(0.175)		
Knowledge of child dev. (raw) n1=171, n0=169 	7.287	7.112	0.174	0.383	
	(1.723)	(1.399)	(0.200)		
Single, divorced, or widowed (%) n1=171, n0=169 	0.240	0.213	0.027	0.591 	
	(0.428)	(0.411)	[0.290]		
*** Significance at 1%, ** Significance at 5%, * Significance at 10%
() Standard errors in brackets
[] Chi2 Statistic, clustered by Fake Municipality ID (bl)
