# Supplementary material for: Early Stimulation and Nutrition: The Impacts of a Scalable Intervention
Source: J Eur Econ Assoc. 2022 Jan 28;20(4):1395–432. doi: 10.1093/jeea/jvac005 (PMC9372035; doi:10.1093/jeea/jvac005)
Supplement: jvac005_Attanasio_etal_Replication-Data-Code [file jvac005_attanasio_etal_replication-data-code.zip › replication-data-code/output/table-f1/Attrition_analysis.doc]

	(1)	(2)	(3)	
VARIABLES	Dependent variable -> Lost at FU		Attrition - Cov*ITT	
				
Treatment assignment	0.0383*	0.0401*	-0.0283	
	(0.0213)	(0.0208)	(0.0462)	
Age (months) (bl)		0.0159*	0.0139	
		(0.0088)	(0.0092)	
Age sqrt (months) (bl)		-0.0010	-0.0012	
		(0.0008)	(0.0008)	
Gender: Male		-0.0176		
		(0.0127)		
First born		0.0394**	0.0277	
		(0.0165)	(0.0253)	
Household wealth index above the median (bl)		-0.0279*	-0.0028	
		(0.0148)	(0.0191)	
Mother's education (years) (bl)		-0.0011		
		(0.0026)		
Father present (bl)		-0.0450**	-0.0585**	
		(0.0208)	(0.0269)	
Household size (bl)		-0.0094		
		(0.0066)		
Mother's PPVT (bl)		-0.0018		
		(0.0011)		
T * edad			0.0090**	
			(0.0039)	
T * first_born			0.0400	
			(0.0337)	
T * indw_alto			-0.0524*	
			(0.0284)	
T * padre_presente			0.0290	
			(0.0411)	
Constant	0.0675***	0.1408***	0.0718**	
	(0.0116)	(0.0469)	(0.0344)	
				
Observations	1,456	1,456	1,456	
R-squared	0.0047	0.0334	0.0334	
F-test	3.229	2.908	3.281	
Prob > F	0.0759	0.00351	0.00123	
Robust standard errors in parentheses
*** p<0.01, ** p<0.05, * p<0.1
