# Supplementary material for: Early Stimulation and Nutrition: The Impacts of a Scalable Intervention
Source: J Eur Econ Assoc. 2022 Jan 28;20(4):1395–432. doi: 10.1093/jeea/jvac005 (PMC9372035; doi:10.1093/jeea/jvac005)
Supplement: jvac005_Attanasio_etal_Replication-Data-Code [file jvac005_attanasio_etal_replication-data-code.zip › replication-data-code/output/table-f2/DropoutMFCCDecision.doc]

	(1)	(2)	(3)	(4)	(5)	(6)	
VARIABLES	Dropout from FAMI	Dropout from FAMI	y1	y1	y1	y1	
							
Treatment Assignment = 1, Treatment	-0.100*	-0.123**	-0.100*	-0.119**	-0.100*	-0.123**	
	(0.056)	(0.051)	(0.056)	(0.051)	(0.056)	(0.051)	
Gender: Male = 1, Male		0.008		0.010		0.010	
		(0.028)		(0.027)		(0.027)	
Previous attendance to a child care center (bl) = 1, Yes		0.027		0.031		0.035	
		(0.056)		(0.058)		(0.059)	
Municipality's population category (bl) = 1, Over 10.000		-0.105*		-0.104*		-0.106*	
		(0.055)		(0.054)		(0.055)	
Household wealth index above the median (bl) = 1, Yes		0.068**		0.069**		0.071**	
		(0.034)		(0.033)		(0.033)	
Teenage mother (bl) = 1, Yes		-0.016		-0.014		-0.017	
		(0.033)		(0.032)		(0.033)	
Mother's PPVT (bl)		0.012***		0.012***		0.011***	
		(0.002)		(0.002)		(0.002)	
Fake Department ID = 2		-0.052		-0.056		-0.044	
		(0.225)		(0.224)		(0.213)	
Fake Department ID = 3		0.032		0.032		0.026	
		(0.060)		(0.063)		(0.064)	
							
Observations	989	989	989	989	989	989	
Robust standard errors in parentheses
*** p<0.01, ** p<0.05, * p<0.1
