# Supplementary material for: Early Stimulation and Nutrition: The Impacts of a Scalable Intervention
Source: J Eur Econ Assoc. 2022 Jan 28;20(4):1395–432. doi: 10.1093/jeea/jvac005 (PMC9372035; doi:10.1093/jeea/jvac005)
Supplement: jvac005_Attanasio_etal_Replication-Data-Code [file jvac005_attanasio_etal_replication-data-code.zip › replication-data-code/output/table-f3/DropoutMFDecision.doc]

	(1)	(2)	(3)	(4)	(5)	(6)	
VARIABLES	Dropout from FAMI	Dropout from FAMI	y1	y1	y1	y1	
							
Treatment Assignment = 1, Treatment	-0.029	-0.051	-0.029	-0.053	-0.029	-0.054	
	(0.048)	(0.046)	(0.048)	(0.047)	(0.048)	(0.048)	
Gender: Male = 1, Male		-0.023		-0.020		-0.021	
		(0.021)		(0.021)		(0.021)	
Previous attendance to a child care center (bl) = 1, Yes		0.015		0.025		0.023	
		(0.042)		(0.047)		(0.050)	
Municipality's population category (bl) = 1, Over 10.000		-0.039		-0.044		-0.041	
		(0.049)		(0.049)		(0.053)	
Household wealth index above the median (bl) = 1, Yes		0.020		0.023		0.022	
		(0.025)		(0.025)		(0.025)	
Teenage mother (bl) = 1, Yes		-0.032		-0.032		-0.034	
		(0.025)		(0.026)		(0.026)	
Mother's PPVT (bl)		0.008***		0.007***		0.007***	
		(0.003)		(0.002)		(0.002)	
Fake Department ID = 2		-0.126***					
		(0.035)					
Fake Department ID = 3		-0.006		-0.013		-0.014	
		(0.040)		(0.045)		(0.045)	
Fake Department ID = 2, omitted				-		-	
							
							
Observations	770	770	770	752	770	752	
Robust standard errors in parentheses
*** p<0.01, ** p<0.05, * p<0.1
