# Supplementary material for: Early Stimulation and Nutrition: The Impacts of a Scalable Intervention
Source: J Eur Econ Assoc. 2022 Jan 28;20(4):1395–432. doi: 10.1093/jeea/jvac005 (PMC9372035; doi:10.1093/jeea/jvac005)
Supplement: jvac005_Attanasio_etal_Replication-Data-Code [file jvac005_attanasio_etal_replication-data-code.zip › replication-data-code/output/table-f4/intermediate_app - Mediana.doc]

VARIABLE	22 o mÃ¡s Contactos	Menor a 22 contactos	22 o mÃ¡s Contactos - Menor a 22 contactos		
Total Observaciones = 701	366	335	Differencia	p-value	
No. of adult books, magazines and newspapers (fu) n1=365, n0=335 	2.542	2.615	-0.072	0.811	
	(3.071)	(3.102)	(0.303)		
No. of toy sources (fu) n1=366, n0=335 	1.331	1.382	-0.051	0.540	
	(0.941)	(0.930)	(0.084)		
No. of varieties of play materials (fu) n1=366, n0=335 	1.331	1.516	-0.186	0.135	
	(1.368)	(1.445)	(0.124)		
No. of varieties of play activities over past 3 days (fu) n1=366, n0=335 	2.448	2.627	-0.179	0.248	
	(1.514)	(1.601)	(0.155)		
No. of parental care activities over the past 3 days (fu) n1=366, n0=335 	4.645	4.863	-0.218	0.000***	
	(1.130)	(0.915)	(0.062)		
Parental investment (fu) n1=365, n0=335 	-0.073	0.047	-0.120	0.159	
	(0.976)	(0.993)	(0.085)		
Social support DUKE UNC-11 total (raw score) (bl) n1=362, n0=331 	40.749	41.770	-1.022	0.277	
	(8.394)	(8.105)	(0.941)		
Mother's self-efficacy above the median (bl) (%) n1=366, n0=334 	0.388	0.410	-0.022	0.724 	
	(0.488)	(0.493)	[0.125]		
Mothers with depression symptoms (bl) (%) n1=363, n0=332 	0.140	0.169	-0.028	0.511 	
	(0.348)	(0.375)	[0.432]		
Use of verbal or physical abuse in the household (bl) (%) n1=366, n0=335 	0.011	0.039	-0.028	0.065* 	
	(0.104)	(0.193)	[3.396]		
*** Significance at 1%, ** Significance at 5%, * Significance at 10%
() Standard errors in brackets
[] Chi2 Statistic, clustered by Fake Municipality ID (bl)
