# Supplementary material for: Early Stimulation and Nutrition: The Impacts of a Scalable Intervention
Source: J Eur Econ Assoc. 2022 Jan 28;20(4):1395–432. doi: 10.1093/jeea/jvac005 (PMC9372035; doi:10.1093/jeea/jvac005)
Supplement: jvac005_Attanasio_etal_Replication-Data-Code [file jvac005_attanasio_etal_replication-data-code.zip › replication-data-code/output/table-f4/nutrition_app - Mediana.doc]

VARIABLE	22 o mÃ¡s Contactos	Menor a 22 contactos	22 o mÃ¡s Contactos - Menor a 22 contactos		
Total Observaciones = 701	366	335	Differencia	p-value	
Weight-for-age z-score (fu) n1=351, n0=310 	0.181	0.338	-0.157	0.264	
	(1.419)	(1.351)	(0.141)		
Length/height-for-age z-score (fu) n1=345, n0=308 	0.001	-0.027	0.028	0.865	
	(1.662)	(1.725)	(0.162)		
BMI-for-age z-score (fu) n1=338, n0=298 	0.327	0.386	-0.059	0.680	
	(1.640)	(1.620)	(0.142)		
Weight-for-length/height z-score (fu) n1=331, n0=294 	0.413	0.320	0.093	0.504	
	(1.563)	(1.621)	(0.139)		
ELCSA Food insecurity (fu) (%) n1=366, n0=335 	0.473	0.537	-0.065	0.455 	
	(0.500)	(0.499)	[0.559]		
*** Significance at 1%, ** Significance at 5%, * Significance at 10%
() Standard errors in brackets
[] Chi2 Statistic, clustered by Fake Municipality ID (bl)
