# Supplementary material for: Early Stimulation and Nutrition: The Impacts of a Scalable Intervention
Source: J Eur Econ Assoc. 2022 Jan 28;20(4):1395–432. doi: 10.1093/jeea/jvac005 (PMC9372035; doi:10.1093/jeea/jvac005)
Supplement: jvac005_Attanasio_etal_Replication-Data-Code [file jvac005_attanasio_etal_replication-data-code.zip › replication-data-code/output/table-f4/sociodemographic_app - Mediana.doc]

VARIABLE	22 o mÃ¡s Contactos	Menor a 22 contactos	22 o mÃ¡s Contactos - Menor a 22 contactos		
Total Observaciones = 701	366	335	Differencia	p-value	
Age (months) (bl) n1=366, n0=335 	5.434	6.066	-0.631	0.008***	
	(3.312)	(3.474)	(0.236)		
Gender: Male (%) n1=366, n0=335 	0.530	0.504	0.026	0.498 	
	(0.500)	(0.501)	[0.458]		
Child's birth weight (gr) n1=358, n0=326 	3,141	3,245	-103	0.037**	
	(543)	(599)	(50)		
Birth order n1=366, n0=335 	1.112	1.003	0.109	0.293	
	(1.349)	(1.346)	(0.104)		
First born (%) n1=366, n0=335 	0.434	0.499	-0.064	0.139 	
	(0.496)	(0.501)	[2.192]		
Mother's education (years) (bl) n1=366, n0=335 	8.581	9.148	-0.567	0.090*	
	(3.481)	(3.332)	(0.334)		
Father present (bl) (%) n1=366, n0=335 	0.738	0.654	0.084	0.023** 	
	(0.440)	(0.476)	[5.198]		
Number of siblings (bl) n1=366, n0=335 	1.060	0.997	0.063	0.536	
	(1.246)	(1.328)	(0.102)		
Mother is married/cohabiting (bl) (%) n1=366, n0=335 	0.713	0.710	0.003	0.959 	
	(0.453)	(0.454)	[0.003]		
Mother is single (bl) (%) n1=366, n0=335 	0.216	0.275	-0.059	0.136 	
	(0.412)	(0.447)	[2.225]		
Mother is divorced (bl) (%) n1=366, n0=335 	0.014	0.009	0.005	0.558 	
	(0.116)	(0.094)	[0.343]		
Teenage mother (bl) (%) n1=366, n0=335 	0.230	0.281	-0.051	0.131 	
	(0.421)	(0.450)	[2.276]		
Mother's age (years) (bl) n1=366, n0=335 	26.768	25.493	1.275	0.032**	
	(6.998)	(6.588)	(0.595)		
Household wealth index (bl) n1=366, n0=334 	0.018	0.105	-0.087	0.363	
	(0.954)	(0.959)	(0.096)		
*** Significance at 1%, ** Significance at 5%, * Significance at 10%
() Standard errors in brackets
[] Chi2 Statistic, clustered by Fake Municipality ID (bl)
