# Supplementary material for: Early Stimulation and Nutrition: The Impacts of a Scalable Intervention
Source: J Eur Econ Assoc. 2022 Jan 28;20(4):1395–432. doi: 10.1093/jeea/jvac005 (PMC9372035; doi:10.1093/jeea/jvac005)
Supplement: jvac005_Attanasio_etal_Replication-Data-Code [file jvac005_attanasio_etal_replication-data-code.zip › replication-data-code/output/table-g1/_Table_Impact_on_children_s_outcomes_MV_RW.doc]

Table X. Impact on children's outcomes
VARIABLE	Impact (95% CI)	P Value	RW P Value	
Bayley-III Factor	0.175	0.009***	0.032**	
	(0.044,0.305)			
ASQ:SE Total Score	-0.075	0.387	0.579	
	(-0.246,0.095)			
Height for age Z-Score	0.075	0.194	0.393	
	(-0.038,0.187)			
