# Supplementary material for: Early Stimulation and Nutrition: The Impacts of a Scalable Intervention
Source: J Eur Econ Assoc. 2022 Jan 28;20(4):1395–432. doi: 10.1093/jeea/jvac005 (PMC9372035; doi:10.1093/jeea/jvac005)
Supplement: jvac005_Attanasio_etal_Replication-Data-Code [file jvac005_attanasio_etal_replication-data-code.zip › replication-data-code/ReadMe.docx]

ReadMe

Data Files and Do Files to replicate results in:

Attanasio, O., Baker-Henningham, H., Bernal, B., Meghir, C., Pineda, D., and Rubio-Codina, M. “Early Stimulation and Nutrition: The Impacts of a Scalable Intervention”, *Journal of the European Economic Association*

In the replication material, we provide all data set and programs used to run the final estimations. Due to confidentiality agreements, we provide fake IDs for the municipalities, departments, and subjects of the study.

1. **Data File:**
2. baseline-characteristics.dta

This is a child-level data set with nutritional and sociodemographic information at baseline.

1. baseline-fami-characteristics.dta

This is a FAMI mother-level data set with sociodemographic and practices information at baseline.

1. compliance-analysis.dta

This is a child-level data set with information on the probability of dropping out of the program or changing to another ECD program and other sociodemographic characteristics at baseline. We use this data set to estimate tables F2 and F3.

1. duration-analysis.dta

This is a child-level data set with information with the attendance to the programs for the treatment arm. It also provides sociodemographic, intermediate outcomes, and nutritional information at baseline. We use this data set to estimate table F4.

1. estimation-data.dta

This is a child-level data set with the information to estimate our main results. It contains outcomes information at follow-up, baseline characteristics, and basic measures of compliance with the program.

1. **Ado Files:**
2. clchi2_modified.ado

This ado file estimates chi2 statistic for dichotomous outcomes on clustered data. The original ado is provided by Jeph Herrin, we modified it to better adjust the output to our tables.

1. equivalence_rw.ado

This ado generates mean differences tables with Romano Wolf adjustment for multiple hypothesis testing.

1. equivalence.ado

This ado generates mean differences tables.

1. fadeout2.ado

This ado generates impact estimates using a hackman model for selection into the follow-up.

1. het_ols.ado

This ado generates tables with heterogeneous effects of the impact estimates.

1. romwolf twostep.do

This ado produces Romano Wolf adjustment for multiple hypothesis testing of the impact estimates.

1. table_bmj.ado

This ado generates basic tables of the impact estimates of the program.

1. **Do Files:**
2. 0-master.do

Each do file is named after the Table in the paper that the results relate to. Running a do file will produce a the results for that table or figure. 0-master.do will run the entire set of tables and figures in one program.
